# Validation of a host blood transcriptomic biomarker for pulmonary tuberculosis in people living with HIV: a prospective diagnostic and prognostic accuracy study

**DOI:** 10.1016/S2214-109X(21)00045-0

**Published:** 2021-04-13

**Authors:** Simon C Mendelsohn, Andrew Fiore-Gartland, Adam Penn-Nicholson, Humphrey Mulenga, Stanley Kimbung Mbandi, Bhavesh Borate, Katie Hadley, Chris Hikuam, Munyaradzi Musvosvi, Nicole Bilek, Mzwandile Erasmus, Lungisa Jaxa, Rodney Raphela, Onke Nombida, Masooda Kaskar, Tom Sumner, Richard G White, Craig Innes, William Brumskine, Andriëtte Hiemstra, Stephanus T Malherbe, Razia Hassan-Moosa, Michèle Tameris, Gerhard Walzl, Kogieleum Naidoo, Gavin Churchyard, Thomas J Scriba, Mark Hatherill, CORTIS-HR Study Team, Charmaine Abrahams, Hadn Africa, Petri Ahlers, Denis Arendsen, Tebogo Badimo, Kagiso Baepanye, Kesenogile Edna Baepanye, Bianca Bande, Nomfuneko Cynthia Batyi, Roslyn Beukes, Laudicia Tshenolo Bontsi, Obakeng Peter Booi, Mari Cathrin Botha, Samentra Braaf, Sivuyile Buhlungu, Alida Carstens, Kgomotso Violet Chauke, Thilagavathy Chinappa, Eva Chung, Michelle Chung, Ken Clarke, Yolundi Cloete, Lorraine Coetzee, Marelize Collignon, Alessandro Companie, Cara-mia Corris, Mooketsi Theophillius Cwaile, Thobelani Cwele, Ilse Davids, Isabella Johanna Davies, Emilia De Klerk, Marwou de Kock, Audrey Lebohang Dhlamini, Bongani Diamond, Maria Didloff, Celaphiwe Dlamini, Palesa Dolo, Candice Eyre, Tebogo Feni, Juanita Ferreira, Christal Ferus, Michelle Fisher, Marika Flinn, Bernadine Fransman, Welseh Phindile Galane, Hennie Geldenhuys, Diann Gempies, Thelma Goliath, Dhineshree Govender, Yolande Gregg, Goodness Gumede, Zanele Gwamada, Senzo Halti, Rieyaat Hassiem, Roxane Herling, Yulandi Herselman, Ellis Hughes, Henry Issel, Blanchard Mbay Iyemosolo, Zandile Jali, Bonita Janse Van Rensburg, Ruwiyda Jansen, James Michael Jeleni, Olebogeng Jonkane, Fabio Julies, Fazlin Kafaar, Christian Mabika Kasongo, Christian Mabika Kasongo, Sophie Keffers, Boitumelo Sophy Kekana, Sebaetseng Jeanette Kekana, Xoliswa Kelepu, Lungile Khanyile, Gomotsegang Virginia Khobedi, Gloria Khomba, Lucky Sipho Khoza, Marietjie King, Gloria Keitumetse Kolobe, Sandra Kruger, Jaftha Kruger, Ndlela Israel Kunene, Sunelza Lakay, Aneesa Lakhi, Nondumiso Langa, Hildah Ledwaba, Lerato Julia Lekagane, Sheiley Christina Lekotloane, Thelma Leopeng, Ilze Jeanette Louw, Angelique Kany Kany Luabeya, Sarah Teboso Lusale, Perfect Tiisetso Maatjie, Immaculate Mabasa, Tshegofatso Dorah Mabe, Kamogelo Fortunate Mabena, Nkosinathi Charles Mabuza, Simbarashe Mabwe, Johanna Thapelo Madikwe, Octavia Mahkosazana Madikwe, Rapontwana Letlhogonolo Maebana, Malobisa Sylvester Magwasha, Molly Majola, Mantai Makhetha, Lebohang Makhethe, Vernon Malay, Vutlhari-I-Vunhenha Fairlord Manzini, Jabu Maphanga, Nonhle Maphanga, Juanita Market, Isholedi Samuel Maroele, Omphile Petunia Masibi, July Rocky Mathabanzini, Tendamudzimu Ivan Mathode, Ellen Ditaba Matsane, Lungile Mbata, Lungile Mbata, Faheema Meyer, Nyasha Karen Mhandire, Thembisiwe Miga, Nosisa Charity Thandeka Mkhize, Caroline Mkhokho, Neo Hilda Mkwalase, Nondzakazi Mnqonywa, Karabo Moche, Brenda Matshidiso Modisaotsile, Patricia Pakiso Mokgetsengoane, Selemeng Matseliso Carol Mokone, Kegomoditswe Magdeline Molatlhegi, Thuso Andrew Molefe, Joseph Panie Moloko, Kabelo Molosi, Motlatsi Evelyn Molotsi, Tebogo Edwin Montwedi, Boikanyo Dinah Monyemangene, Hellen Mokopi Mooketsi, Miriam Moses, Boitumelo Mosito, Tshplpfelo Mapula Mosito, Ireen Lesebang Mosweu, Primrose Mothaga, Banyana Olga Motlagomang, Angelique Mouton, Onesisa Mpofu, Funeka Nomvula Mthembu, Mpho Mtlali, Nhlamulo Ndlovu, Nompumelelo Ngcobo, Julia Noble, Bantubonke Bertrum Ntamo, Gloria Ntanjana, Tedrius Ntshauba, Fajwa Opperman, Nesri Padayatchi, Thandiwe Papalagae, Christel Petersen, Themba Phakathi, Mapule Ozma Phatshwane, Patiswa Plaatjie, Abe Pretorius, Victor Kgothatso Rameetse, Dirhona Ramjit, Frances Ratangee, Maigan Ratangee, Pearl Nomsa Sanyaka, Alicia Sato, Elisma Schoeman, Constance Schreuder, Letlhogonolo Seabela, Kelebogile Magdeline Segaetsho, Ni Ni Sein, Raesibe Agnes Pearl Selepe, Melissa Neo Senne, Melissa Neo Senne, Alison September, Cashwin September, Moeti Serake, Justin Shenje, Thandiwe Shezi, Sifiso Cornelius Shezi, Phindile Sing, Chandrapharbha Singh, Zona Sithetho, Dorothy Solomons, Kim Stanley, Marcia Steyn, Bongiwe Stofile, Sonia Stryers, Liticia Swanepoel, Anne Swarts, Mando Mmakhora Thaba, Lethabo Collen Theko, Philile Thembela, Mugwena Thompo, Asma Toefy, Khayalethu Toto, Dimakatso Sylvia Tsagae, Ayanda Tsamane, Vincent Tshikovhi, Lebogang Isaac Tswaile, Petrus Tyambetyu, Susanne Tönsing, Habibullah Valley, Linda van der Merwe, Elma van Rooyen, Ashley Veldsman, Helen Veldtsman, Kelvin Vollenhoven, Londiwe Zaca, Elaine Zimri, Mbali Zulu

**Affiliations:** a South African Tuberculosis Vaccine Initiative, Institute of Infectious Disease and Molecular Medicine, Division of Immunology, Department of Pathology, University of Cape Town, Cape Town, South Africa; b Vaccine and Infectious Disease Division, Fred Hutchinson Cancer Research Center, Seattle, WA, USA; c TB Modelling Group, TB Centre, Centre for Mathematical Modelling of Infectious Diseases, Department of Infectious Disease Epidemiology, London School of Hygiene & Tropical Medicine, London, UK; d The Aurum Institute, Johannesburg, South Africa; e DST/NRF Centre of Excellence for Biomedical TB Research, Division of Molecular Biology and Human Genetics, Department of Biomedical Sciences, Stellenbosch University, Cape Town, South Africa; f SAMRC Centre for TB Research, Division of Molecular Biology and Human Genetics, Department of Biomedical Sciences, Stellenbosch University, Cape Town, South Africa; g Centre for the AIDS Programme of Research in South Africa, Durban, South Africa; h MRC-CAPRISA HIV-TB Pathogenesis and Treatment Research Unit, Doris Duke Medical Research Institute, University of KwaZulu-Natal, Durban, South Africa; i School of Public Health, University of Witwatersrand, Johannesburg, South Africa

## Abstract

**Background:**

A rapid, blood-based triage test that allows targeted investigation for tuberculosis at the point of care could shorten the time to tuberculosis treatment and reduce mortality. We aimed to test the performance of a host blood transcriptomic signature (RISK11) in diagnosing tuberculosis and predicting progression to active pulmonary disease (prognosis) in people with HIV in a community setting.

**Methods:**

In this prospective diagnostic and prognostic accuracy study, adults (aged 18–59 years) with HIV were recruited from five communities in South Africa. Individuals with a history of tuberculosis or household exposure to multidrug-resistant tuberculosis within the past 3 years, comorbid risk factors for tuberculosis, or any condition that would interfere with the study were excluded. RISK11 status was assessed at baseline by real-time PCR; participants and study staff were masked to the result. Participants underwent active surveillance for microbiologically confirmed tuberculosis by providing spontaneously expectorated sputum samples at baseline, if symptomatic during 15 months of follow-up, and at 15 months (the end of the study). The coprimary outcomes were the prevalence and cumulative incidence of tuberculosis disease confirmed by a positive Xpert MTB/RIF, Xpert Ultra, or Mycobacteria Growth Indicator Tube culture, or a combination of such, on at least two separate sputum samples collected within any 30-day period.

**Findings:**

Between March 22, 2017, and May 15, 2018, 963 participants were assessed for eligibility and 861 were enrolled. Among 820 participants with valid RISK11 results, eight (1%) had prevalent tuberculosis at baseline: seven (2·5%; 95% CI 1·2–5·0) of 285 RISK11-positive participants and one (0·2%; 0·0–1·1) of 535 RISK11-negative participants. The relative risk (RR) of prevalent tuberculosis was 13·1 times (95% CI 2·1–81·6) greater in RISK11-positive participants than in RISK11-negative participants. RISK11 had a diagnostic area under the receiver operating characteristic curve (AUC) of 88·2% (95% CI 77·6–96·7), and a sensitivity of 87·5% (58·3–100·0) and specificity of 65·8% (62·5–69·0) at a predefined score threshold (60%). Of those with RISK11 results, eight had primary endpoint incident tuberculosis during 15 months of follow-up. Tuberculosis incidence was 2·5 per 100 person-years (95% CI 0·7–4·4) in the RISK11-positive group and 0·2 per 100 person-years (0·0–0·5) in the RISK11-negative group. The probability of primary endpoint incident tuberculosis was greater in the RISK11-positive group than in the RISK11-negative group (cumulative incidence ratio 16·0 [95% CI 2·0–129·5]). RISK11 had a prognostic AUC of 80·0% (95% CI 70·6–86·9), and a sensitivity of 88·6% (43·5–98·7) and a specificity of 68·9% (65·3–72·3) for incident tuberculosis at the 60% threshold.

**Interpretation:**

RISK11 identified prevalent tuberculosis and predicted risk of progression to incident tuberculosis within 15 months in ambulant people living with HIV. RISK11's performance approached, but did not meet, WHO's target product profile benchmarks for screening and prognostic tests for tuberculosis.

**Funding:**

Bill & Melinda Gates Foundation and the South African Medical Research Council.

## Introduction

There is an urgent need for earlier tuberculosis diagnosis. By use of novel non-sputum approaches linked to more effective preventive and curative drugs, tuberculosis transmission and mortality could be reduced.^1^, ^2^ The current armamentarium of screening and diagnostic tests is insufficient to curb the tuberculosis pandemic. Symptom screening, the principal case-finding method worldwide, is a poor triage tool for tuberculosis in people living with HIV, with a sensitivity of 51% and a specificity of 70·7% for people on antiretroviral therapy (ART) and a sensitivity of 89·4% and a specificity of 28·1% for ART-naive individuals.^3^ The low specificity of symptom screening leads to many unnecessary and expensive confirmatory tests, and poor sensitivity in ART-treated people misses approximately half of active tuberculosis cases in people living with HIV. Several rapid and cheap non-sputum-based diagnostic tests have shown promise at the point of care; in a prospective study of individuals initiating ART with CD4 cell counts less than 350 cells per μL, C-reactive protein testing had 89·0% sensitivity and 72·1% specificity, and significantly reduced the number of confirmatory Xpert MTB/RIF tests.^4^, ^5^ The Determine TB lipoarabinomannan (LAM) Ag assay had a sensitivity of only 31% among unselected outpatients with HIV in a Cochrane review.^6^

**Research in context**

**Evidence before this study**

We searched MEDLINE, Scopus, and EBSCOHost databases for all studies published in English between database inception and May 31, 2020, using the search terms “diagnostic OR prognostic”, “blood”, “transcriptomic”, “biomarker”, “tuberculosis”, and “HIV”. We identified 17 studies reporting 27 host blood transcriptomic biomarkers that differentiate people living with HIV and tuberculosis from people living with HIV, those with latent *Mycobacterium tuberculosis* infection, or those with other respiratory diseases. Ten studies used the same RNA microarray dataset of people living with HIV in Malawi and South Africa for either training or validation, potentially limiting generalisability. 16 of the 17 studies used case-control or cross-sectional designs, eliminating diagnostic uncertainty and introducing inherent spectrum bias, which would potentially inflate diagnostic accuracy and limit real-world generalisability. Only one prospective diagnostic accuracy study tested performance in all enrolled participants, including those with microbiologically confirmed or clinically suspected tuberculosis, representative of the target population. There were no prospective cohort studies evaluating the prognostic performance of a host blood transcriptomic biomarker in people living with HIV, and no diagnostic accuracy studies in ambulant, predominantly asymptomatic community volunteers living with HIV. Published host blood transcriptomic signatures of tuberculosis are predominantly comprised of interferon (IFN)-stimulated genes known to be induced by viral infection, resulting in elevated signature scores in individuals with detectable HIV viral loads. Signatures developed in people without HIV might perform poorly in people with HIV. However, a prospective comparison of 27 transcriptomic signatures in symptomatic adults reported that the diagnostic accuracy for tuberculosis of the four best performing signatures was not affected by HIV infection. This finding was limited by a small sample size. No studies have evaluated clinical factors affecting transcriptomic signature scores in people with HIV.

**Added value of this study**

To our knowledge, this study is the first to prospectively use a real-time PCR host blood transcriptomic signature (RISK11) to diagnose prevalent tuberculosis and predict progression to incident tuberculosis in a real-world community cohort of ambulant people living with HIV. We found a 2·1% prevalence of previously undiagnosed, predominantly subclinical tuberculosis microbiologically confirmed on one or more sputum samples. Among people with HIV without prevalent tuberculosis at screening, most of whom were on antiretroviral therapy, the rate of progression to incident tuberculosis (microbiologically confirmed on one or more sputum samples) was 2·3 per 100 person-years. RISK11 diagnosed prevalent tuberculosis and predicted progression to incident tuberculosis with a performance approaching, but not meeting, the minimal WHO target product profile for tuberculosis triage and prognostic tests. The IFN-γ release assay was not a good predictor of progression to tuberculosis, which underscores its limited utility in tuberculosis-endemic settings.

**Implications of all the available evidence**

Traditional symptom screening would miss most undiagnosed prevalent tuberculosis in people with HIV in this community setting. The IFN-γ release assay would result in considerable overtreatment with preventive therapy. Host blood transcriptomic biomarkers have promise for community-based tuberculosis screening of people with HIV to guide targeted confirmatory diagnostic testing, enhanced surveillance, and preventive therapy. This study supports further development of host blood transcriptomic signatures into point-of-care testing devices for use in community-based screening of people with HIV.

The only tests currently recommended by WHO to diagnose *Mycobacterium tuberculosis* infection are tuberculin skin testing and the interferon-γ (IFN-γ) release assay.^7^ One systematic review and meta-analysis showed that the incidence of progression to active tuberculosis in people with HIV and a positive tuberculin skin test or IFN-γ release assay was greater than 11-times higher than in those with a negative test.^8^ However, the IFN-γ release assay is expensive, laborious, and requires skilled technicians in a centralised laboratory. Tuberculin skin testing is cheap and can be done at the point of care, but still requires the individual being tested to return for the result to be read within 48 h and has a low positive predictive value.^9^

A rapid, blood-based triage test that allows targeted investigation for tuberculosis at the point of care could shorten the time to tuberculosis treatment. Over the past decade, several host blood transcriptomic biomarkers have emerged with potential for diagnosing prevalent tuberculosis^10^ and predicting progression^11^ to disease. However, 16 of 17 host blood transcriptomic biomarker discovery or validation studies in people living with HIV have used a cross-sectional or case-control design, and no studies have evaluated prospective, community-based screening in an unselected cohort of ambulant people with HIV, or evaluated performance for predicting progression to incident tuberculosis in people with HIV.

We previously developed and validated a highly specific 16-gene biomarker to identify *M tuberculosis*-infected, HIV-uninfected adults with high risk of progression to active tuberculosis disease.^12^ In a patient-level pooled meta-analysis, this 16-gene signature was among eight transcriptomic signatures that achieved the minimum accuracy benchmark in WHO's target product profile for incipient tuberculosis tests.^11^ This PCR-based, transcriptomic, host-response blood signature was refined from a 16-gene to an 11-gene signature named RISK11, with equivalent diagnostic and prognostic performance.^13^ At the time this study was conceived, RISK11 was the only published parsimonious quantitative RT-PCR transcriptomic signature shown to predict progression to incident tuberculosis. Among adults without HIV recruited from the same communities as this study, RISK11 was able to detect microbiologically confirmed pulmonary tuberculosis at enrolment (area under the receiver operating characteristic curve [AUC] 77%) and predict progression to incident tuberculosis within 12 months (AUC 80%).^14^ However, results from a cross-sectional study suggest that performance of RISK11 at discriminating prevalent tuberculosis disease from latent *M tuberculosis* infection in people with HIV might be reduced compared with performance in people without HIV.^15^

To our knowledge, we report the first prospective study of RISK11 to diagnose prevalent tuberculosis and predict progression to incident tuberculosis in people with HIV in a community setting. We aimed to establish whether RISK11 testing meets the minimum WHO target product profile for a triage test for diagnosing prevalent tuberculosis (90% sensitivity and 70% specificity)^16^ or an incipient tuberculosis test for predicting progression to active pulmonary tuberculosis (75% sensitivity and 75% specificity).^17^ Exploratory aims were to compare the performance of RISK11 in diagnosing pulmonary tuberculosis with the performance of symptom-based screening and the LAM assay, and to compare the prognostic performance of RISK11 with that of the IFN-γ release assay.

## Methods

### Study design and participants

In this prospective diagnostic and prognostic accuracy study, we enrolled adults with HIV from five communities in South Africa that were selected because of their high tuberculosis burden. Participants self-identified as HIV-positive; however, confirmatory HIV testing was done at screening. Community-based, consecutive recruitment of participants without clinical suspicion of tuberculosis was by word-of-mouth, house-to-house visits, and liaison with non-governmental organisations. Recruitment did not specifically target household contacts, individuals seeking health care, or other groups at increased risk of tuberculosis. Eligible participants were aged 18–59 years (the adult age range with the highest risk of tuberculosis in South Africa^18^), and did not have known tuberculosis disease or household exposure to a person with multidrug-resistant tuberculosis within the past 3 years. Individuals with a history of tuberculosis within the previous 3 years were excluded to limit false-positive Xpert MTB/RIF and Xpert Ultra results due to the presence of dead mycobacteria or DNA. Individuals with comorbid risk factors for tuberculosis were excluded to avoid confounding test performance in people living with HIV. Participants were excluded if they were pregnant or lactating, or had any medical, surgical, or other condition that would interfere with the study.

This study is reported in accordance with the Standards for Reporting Diagnostic Accuracy Studies initiative guidelines.^19^ The study protocol was approved by the Institutional Human Ethics Committees of each participating site. All participants provided written informed consent in their language of choice. The study protocol can be found in the appendix (pp 16–42).

### Masking

Participants and the study staff responsible for tuberculosis screening were masked to RISK11 status and IFN-γ release assay results, but were not masked to tuberculosis microbiology results. Immunology laboratory staff were not masked to RISK11 status or IFN-γ release assay results, but were masked to tuberculosis disease status. Before the database lock, the statistical analysis team were masked to RISK11 and IFN-γ release assay results. RISK11 and IFN-γ release assay results from the South African Tuberculosis Vaccine Initiative immunology laboratory (Cape Town, South Africa), and tuberculosis microbiology results from the Bio Analytical Research Corporation South Africa (Johannesburg, South Africa) were maintained in different files, which were not integrated until the database had been cleaned and locked.

### Procedures

Screening procedures included taking a medical history, physical examination, and a HIV rapid antibody test. Enrolment procedures included tuberculosis screening, urine collection for the lateral flow Determine TB LAM Ag assay (Alere, Waltham, MA, USA; now owned by Abbott), and phlebotomy for CD4 cell count, HIV plasma viral load in all ART-naive participants (post-hoc also in a random subset of ART-treated participants), RISK11 (using PAXgene RNA tubes [PreAnalytiX, Hombrechtikon, Switzerland]), and the IFN-γ release assay (QuantiFERON TB Gold-Plus, Qiagen, Hilden, Germany; appendix pp 5–6). ART-naive participants were referred for ART and isoniazid preventive therapy per country guidelines. Participants attended up to seven study visits, comprising three telephone calls or field visits at months 1, 2, and 9, and four site visits at months 3, 6, 12, and 15 (the end of study visit).

Two spontaneously expectorated sputum samples, one for liquid mycobacterial culture (Mycobacteria Growth Indicator Tube [MGIT], BACTEC, Beckton Dickinson, Franklin Lakes, NJ, USA) and one for Xpert MTB/RIF (at baseline; Cepheid, Sunnyvale, CA, USA) or Xpert Ultra (at the end of the study; Cepheid, Sunnyvale, CA, USA), were collected from all participants at enrolment and at month 15. Sputum induction was not done as it is not the current standard of care in South Africa. Participants who were sputum-unproductive were assumed sputum-negative. Participants underwent additional tuberculosis investigation (two sputum samples; one for Xpert MTB/RIF and one for liquid mycobacterial culture) if they reported tuberculosis symptoms at study visits during 15 months of follow-up. Tuberculosis symptoms were identified by use of a modified WHO screen, and comprised at least one of: persistent unexplained cough, night sweats, fever, weight loss for 2 weeks or more, or any haemoptysis. If only one sputum result was positive, two additional sputum samples were collected. All participants diagnosed with microbiologically confirmed tuberculosis at baseline or during follow-up were withdrawn from the study and referred for curative treatment. Participants who were diagnosed with clinical tuberculosis or treated off-study, but did not meet the study endpoint definition, were also withdrawn.

### Outcomes

The coprimary outcomes were the prevalence and cumulative incidence of tuberculosis disease confirmed by a positive Xpert MTB/RIF, Xpert Ultra, or MGIT culture, or a combination of such, on at least two separate sputum samples collected within any 30-day period. The secondary endpoint was tuberculosis disease microbiologically confirmed on at least one sputum sample. All Xpert Ultra trace positive results (ie, the lowest bacillary burden for *M tuberculosis* detection) were excluded from the analysis because of the risk of false positives.

### Statistical analysis

We estimated the expected number of prevalent and incident tuberculosis cases in HIV-infected participants on the basis of data from previous and ongoing studies in South Africa. The target sample size was calculated by use of a stochastic model (appendix pp 50–51). Statistical analyses were done in RStudio, version 1.2.5001. Diagnostic performance for prevalent tuberculosis was evaluated in all participants who completed baseline endpoint evaluation. Evaluation of prognostic performance for incident tuberculosis excluded participants with prevalent tuberculosis at baseline and those who did not attend a follow-up visit. Participants with only one sputum sample positive for *M tuberculosis* at enrolment did not meet the primary two-sample endpoint and therefore were included in analyses of the primary incident endpoints, censored at their final study visit. Participants who discontinued follow-up before 15 months, including participants diagnosed with clinical tuberculosis not meeting study endpoint definitions, were included in the analysis as negative controls, but censored at their final visit or last negative sputum sample collection. Participants with a RISK11 score of 60% or higher were classified a priori as RISK11-positive; those with a RISK11 score less than 60% were classified as RISK11-negative. A 60% threshold was selected because it balanced sensitivity and specificity for prediction of incident tuberculosis in case-control studies.^20^ Participants without RISK11 results due to failed collection of the PAXgene sample and participants with an indeterminate RISK11 result due to failure of RT-PCR were excluded from the analysis.

The primary analysis evaluated relative risk (RR) for tuberculosis at baseline and the cumulative incidence ratio (CIR) during 15 months of follow-up in RISK11-positive participants compared with RISK11-negative participants. Cumulative incidence (probability) was estimated in each group by use of a time-dependent non-parametric method (Nelson–Aalen estimator of cumulative hazard; appendix p 6). The 95% CIs and p values were calculated by use of a Wald-based approach. The 95% CIs on tuberculosis prevalence were calculated by use of the binomial Wilson method and the 95% CIs on RR were calculated with a likelihood score-based approach. p values for RR were calculated by use of the χ^2^-squared test.

Binary receiver operating characteristic (ROC) curve analysis was done to calculate the diagnostic AUC. Diagnostic performance metrics, including sensitivity, specificity, positive predictive value, and negative predictive value, were calculated by use of standard formulas on the basis of binary endpoint indicators at enrolment. Prognostic performance metrics were calculated by use of non-parametric methods for time-dependent ROC curve analysis (appendix p 6), which allowed all participants (including those who discontinued early) to contribute to the analysis. The 95% CIs on diagnostic and prognostic performance estimates were calculated by use of a percentile bootstrap with 10 000 samples. In a post-hoc analysis, prognostic performance was qualitatively compared between 12 months and 15 months of follow-up for the secondary endpoint by use of identical methods.

Post-hoc group comparisons were done by use of Pearson's χ^2^ test (for categorical data) or the Wilcoxon rank sum test (for continuous data) and corrected for multiple comparisons by use of the Benjamini–Hochberg procedure.^21^ Post-hoc correlation analysis (Spearman's rank correlation) of RISK11 score and HIV viral load was done in all participants without tuberculosis and with a detectable baseline viral load (>100 copies per mL). Post-hoc analysis of correlation between RISK11 score and the interferon-γ release assay was also done by use of the secondary endpoint prevalent and incident cases, and non-tuberculosis controls (Spearman's rank correlation).

Post-hoc univariable linear regression models were built by use of RISK11 signature score as the dependent variable among all individuals that did not develop tuberculosis to assess the effect of participant characteristics on RISK11 score; collinear predictors (eg, body-mass index) were excluded. Sex, age, and covariables that significantly (p<0·05) predicted RISK11 signature score in the univariable linear models were added to a multivariable linear regression model of RISK11 score to account for confounding and interaction.

Two post-hoc qualitative sensitivity analyses were done by use of methods identical to the primary analysis. The first sensitivity analysis assessed how inclusion of participants with sputum-negative, clinically diagnosed tuberculosis would have affected RISK11 prognostic performance. The second sensitivity analysis excluded participants that were receiving isoniazid preventive therapy at study enrolment or started isoniazid preventive therapy during study follow-up from the diagnostic and prognostic performance analysis. The prespecified statistical analysis plan is detailed in the appendix (pp 43–86).

### Role of the funding source

Subject-specific experts at the Bill & Melinda Gates Foundation contributed to scientific discussions relating to protocol development and study design. The funders of the study had no role in data collection, data analysis, data interpretation, or writing of the report.

## Results

**Figure 1:**
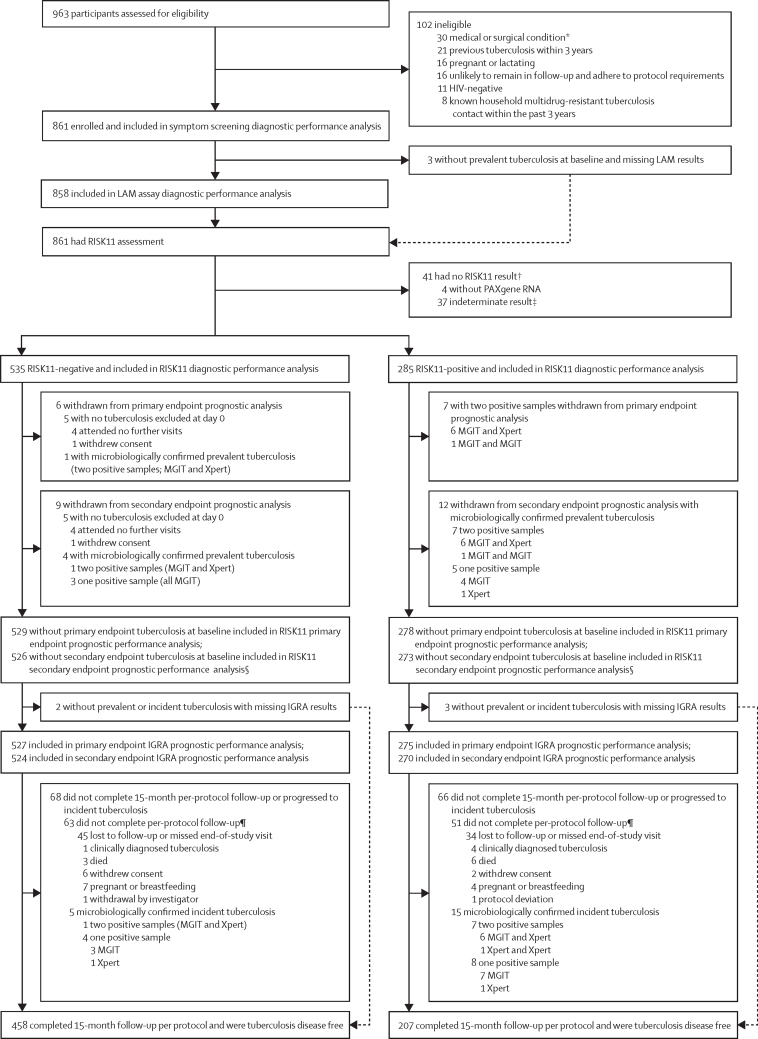
Trial profile IGRA=interferon-γ release assay. LAM=lipoarabinomannan. MGIT=Mycobacteria Growth Indicator Tube. *Any medical, surgical, or other condition, including, but not limited to, known diabetes (requiring oral or injectable therapy), liver disease, or alcohol misuse disorder, that in the opinion of the investigator is likely to interfere with RISK11 performance; safety or efficacy of antiretroviral or isoniazid preventive therapy; or adherence to protocol requirements. †One enrolled participant without a PAXgene RNA sample and one with an indeterminate RISK11 result at baseline had primary endpoint prevalent tuberculosis. One participant with an indeterminate RISK11 result at baseline progressed to primary endpoint incident tuberculosis during follow-up. ‡Probably due to inadequate quality of the RNA sample. §Eight participants with one sputum sample-positive prevalent tuberculosis (three in the RISK11-negative group and five in the RISK11-positive group) were included in the primary endpoint (two or more positive samples) prognostic performance analysis, but excluded from the secondary endpoint (one or more positive samples) prognostic performance analysis. ¶Participants who did not complete follow-up per-protocol were included in the RISK11 and IGRA prognostic performance analysis but censored as non-tuberculosis controls at their last study visit.

Between March 22, 2017, and May 15, 2018, 963 participants were consented and 861 were enrolled (figure 1). Common reasons for exclusion were comorbid conditions and tuberculosis disease within the previous 3 years (figure 1). Table 1 shows baseline characteristics of the study cohort. At baseline, ten of 861 enrolled participants were diagnosed with primary endpoint tuberculosis, with an overall prevalence of 1·2% (binomial Wilson 95% CI 0·6–2·1), and 18 were diagnosed with secondary endpoint tuberculosis, with an overall prevalence of 2·1% (1·3–3·3; table 1). The prevalence of tuberculosis ranged from 0·0–6·7% across the five sites (appendix p 9). Seven (70%) of ten participants with primary endpoint prevalent tuberculosis and 15 (83%) of 18 participants with secondary endpoint prevalent tuberculosis reported no tuberculosis symptoms on screening. During a median follow-up of 15 months (IQR 15–15), nine participants progressed to primary endpoint incident tuberculosis, with an overall incidence of 1·0 per 100 person-years (Nelson–Aalen 95% CI 0·3–1·6), and 21 participants progressed to secondary endpoint incident tuberculosis, with an overall incidence of 2·3 per 100 person-years (1·3–3·2; table 1).

**Table 1:** Baseline characteristics of the study cohort and tuberculosis endpoints by RISK11 status

		Enrolled cohort (n=861)*	**RISK11-positive (n=285)**	**RISK11-negative (n=535)**	**RISK11-positive *vs* RISK11-negative p value**†
**Baseline characteristics at study enrolment**					
Sex					
	Female	621 (72%)	198 (69%)	396 (74%)	0·17
	Male	240 (28%)	87 (31%)	139 (26%)	..
Median age, years		35 (29–42)	33 (28–41)	35 (29–42)	0·023
Ethnicity					
	Black African	724 (84%)	224 (79%)	465 (87%)	0·0019
	Mixed ancestry	137 (16%)	61 (21%)	70 (13%)	..
Median body-mass index, kg/m^2^		24·2 (20·6–31·2)	23·0 (19·8–29·0)	25·3 (21·2–31·8)	<0·0001
History of cigarette smoking‡		334 (39%)	121 (42%)	196 (37%)	0·10
Previous tuberculosis		212 (25%)	70 (25%)	131 (24%)	0·98
Tuberculosis household contacts		160 (19%)	50 (18%)	105 (20%)	0·47
Interferon-γ release assay result					
	Not available	7 (1%)	3 (1%)	2 (0%)	0·10
	Negative	461 (54%)	166 (58%)	279 (52%)	..
	Positive	393 (46%)	116 (41%)	254 (47%)	..
On isoniazid preventive therapy at enrolment		47 (5%)	9 (3%)	37 (7%)	0·026
Started isoniazid preventive therapy during study		370/814 (45%)	104/276 (38%)	249/498 (50%)	0·0010
Antiretroviral therapy at enrolment					
	Naive	193 (22%)	107 (38%)	77 (14%)	<0·0001
	<6 months	115 (13%)	39 (14%)	68 (13%)	..
	6–12 months	66 (8%)	23 (8%)	42 (8%)	..
	>12 months	487 (57%)	116 (41%)	348 (65%)	..
Started antiretroviral therapy during study		142/193 (74%)	82/107 (77%)	56/77 (73%)	0·55
Median CD4 cell count, cells per μL		529·0 (349·5–724·5)	370·0 (234·0–562·2)	606·0 (431·0–802·8)	<0·0001
Positive for tuberculosis symptoms		51 (6%)	24 (8%)	23 (4%)	0·016
**Tuberculosis endpoints**					
Prevalent tuberculosis, n (%; 95% CI)					
	Primary endpoint (two or more positive samples)	10 (1·2%; 0·6–2·1)	7 (2·5%; 1·2–5·0)	1 (0·2%; 0·0–1·1)	NA§
	Secondary endpoint (one or more positive samples)	18 (2·1%; 1·3–3·3)	12 (4·2%; 2·4–7·2)	4 (0·7%; 0·3–1·9)	NA§
Incident tuberculosis, n (rate per 100 person-years; 95% CI)					
	Primary endpoint (two or more positive samples)	9 (1·0; 0·3–1·6)	7 (2·5; 0·7–4·4)	1 (0·2; 0·0–0·5)	NA§
	Secondary endpoint (one or more positive samples)	21 (2·3; 1·3–3·2)	15 (5·2; 2·6–7·6)	5 (0·9; 0·1–1·6)	NA§

Data are n (%), median (IQR), or n/N (%), unless otherwise specified. Baseline characteristics and tuberculosis endpoints by site can be found in the appendix (p 9). NA=not applicable.

* Includes the participants with indeterminate (n=37) or missing (n=4) RISK11 scores.

† p values from the Wilcoxon Rank Sum test (for continuous data) or Pearson's χ^2^ test (for categorical data).

‡ Self-defined current or past cigarette smoking (ie, anyone who regarded themselves as a current or former cigarette smoker).

§ See table 2 and table 3 for RISK11 diagnostic and prognostic performance.

Of the enrolled participants, 820 (95%) had a valid RISK11 result, of whom 535 (65%) were RISK11-negative and 285 (35%) were RISK11-positive (table 1). Participants with indeterminate RISK11 results were excluded from the analysis. One enrolled participant without a PAXgene RNA sample available and one with an indeterminate RISK11 result at baseline had primary endpoint prevalent tuberculosis. One participant with an indeterminate RISK11 result at baseline progressed to primary endpoint incident tuberculosis during follow-up. The proportion of RISK11-positive participants ranged from 22% to 45% across the five sites (appendix p 9). 665 (81%) of the 820 participants with RISK11 results completed 15 months of follow-up per protocol and remained free of tuberculosis disease; 119 (15%) participants with RISK11 results did not complete the study, because of withdrawal, death, or loss to follow-up. Early study discontinuation (incomplete per-protocol follow-up) was higher in the RISK11-positive group (51 [18%] of 278) than in the RISK11-negative group (63 [12%] of 529; Pearson's χ^2^ p=0·031; figure 1; appendix p 10).

There were no differences in sex, smoking history, previous tuberculosis, household contact with tuberculosis, or IFN-γ release assay status between RISK11-positive and RISK11-negative participants (χ^2^ p>0·05; table 1). A significantly greater proportion of RISK11-positive participants than RISK11-negative participants were of mixed ancestry (table 1). Median body-mass index was significantly lower in RISK11-positive participants than in RISK11-negative participants, and a higher proportion of RISK11-positive than RISK11-negative participants reported one or more tuberculosis symptoms at enrolment (table 1). Compared with RISK11-negative participants, a higher proportion of RISK11-positive participants were ART-naive and, correspondingly, had lower median CD4 cell counts (table 1). Fewer participants in the RISK11-positive group than in the RISK11-negative group were receiving isoniazid preventive therapy at enrolment or commenced isoniazid preventive therapy during the study (table 1). History of isoniazid preventive therapy before study enrolment was not recorded.

**Figure 2:**
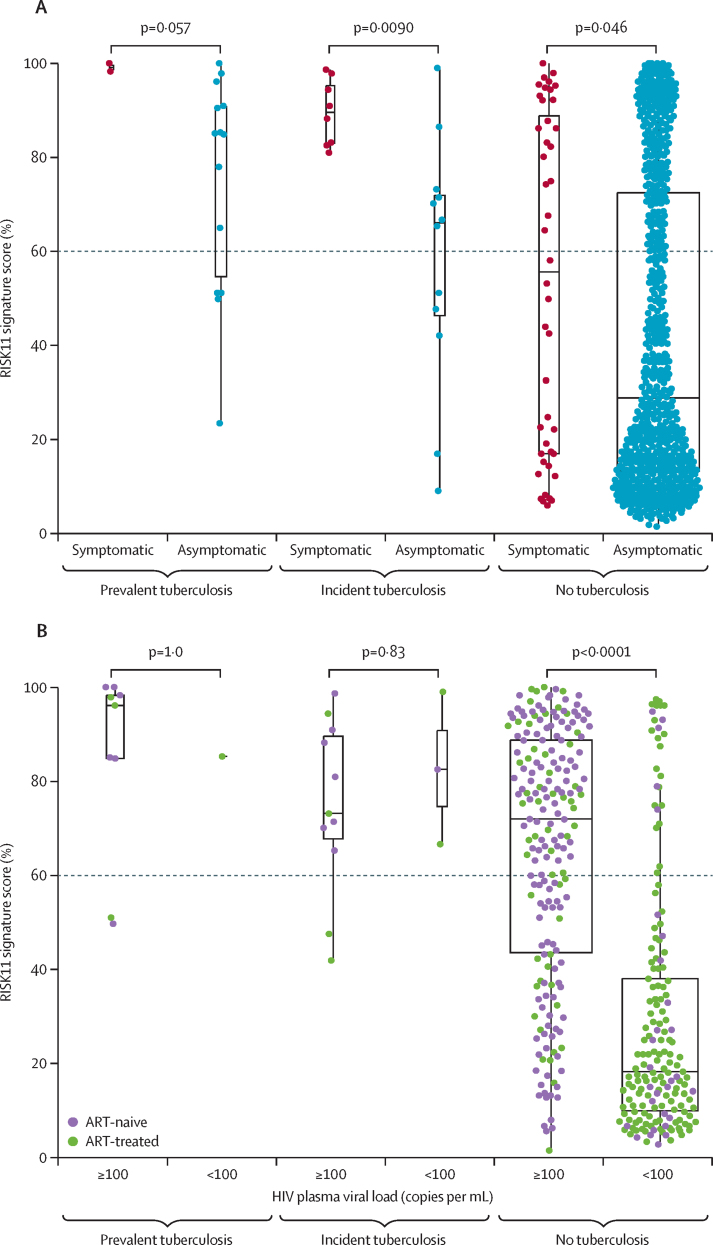
RISK11 signature score distribution (A) RISK11 signature scores by symptom status. Box-and-whisker plots depicting RISK11 signature scores measured at enrolment (each dot represents a participant) in participants with symptomatic, clinical tuberculosis, asymptomatic, subclinical tuberculosis, or no tuberculosis. (B) RISK11 signature scores measured at enrolment by HIV plasma viral load (copies per mL). Prevalent and incident tuberculosis comprised all microbiologically confirmed secondary endpoint cases. Symptoms were recorded at the time of diagnosis for participants with prevalent and incident tuberculosis, and at enrolment for participants without tuberculosis. p values for comparison of median RISK11 signature scores between groups in box-and-whisker plots were calculated with the Mann-Whitney *U* test and corrected for multiple comparisons by use of the Benjamini-Hochberg Procedure.^21^ Boxes depict the IQR, the midline represents the median, and the whiskers indicate the IQR ± (1·5 × IQR). The dashed line depicts the a priori RISK11 score threshold (60%).

Among participants without tuberculosis, the median RISK11 score was higher in participants with at least one tuberculosis symptom (median 55·5% [IQR 16·9–88·8]) than in those without symptoms (28·8% [13·1–72·4]; Wilcoxon rank-sum p=0·046; figure 2A). A similar trend was also observed in participants with asymptomatic (subclinical) and symptomatic tuberculosis; the two participants with symptomatic prevalent tuberculosis had high RISK11 scores, both greater than 90% (figure 2A). Among participants without tuberculosis, the median RISK11 score was also higher for participants with HIV plasma viral loads of 100 copies per mL or more (median 72·0% [IQR 43·5–88·7]) than for participants with viral loads less than 100 copies per mL (18·2% [10·0–38·0]; p<0·0001; figure 2B). Among 198 participants without tuberculosis and a HIV viral load of 100 copies per mL or more, there was a weak but significant correlation between HIV plasma viral load and RISK11 signature score (Spearman *r*=0·29; p<0·0001; appendix p 15).

**Figure 3:**
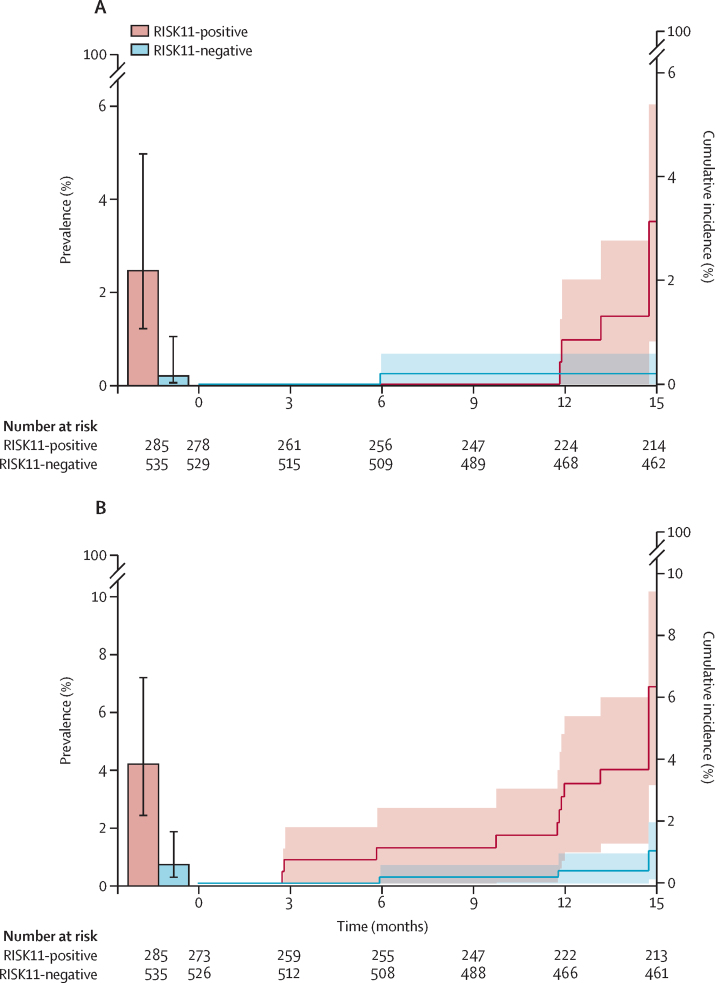
Prevalence and cumulative incidence of primary and secondary endpoint tuberculosis Prevalence and cumulative incidence of primary (A) and secondary (B) endpoint tuberculosis in RISK11-positive and RISK11-negative participants at study enrolment and over 15 months of follow-up. Error bars and shaded areas represent the 95% CIs.

**Figure 4:**
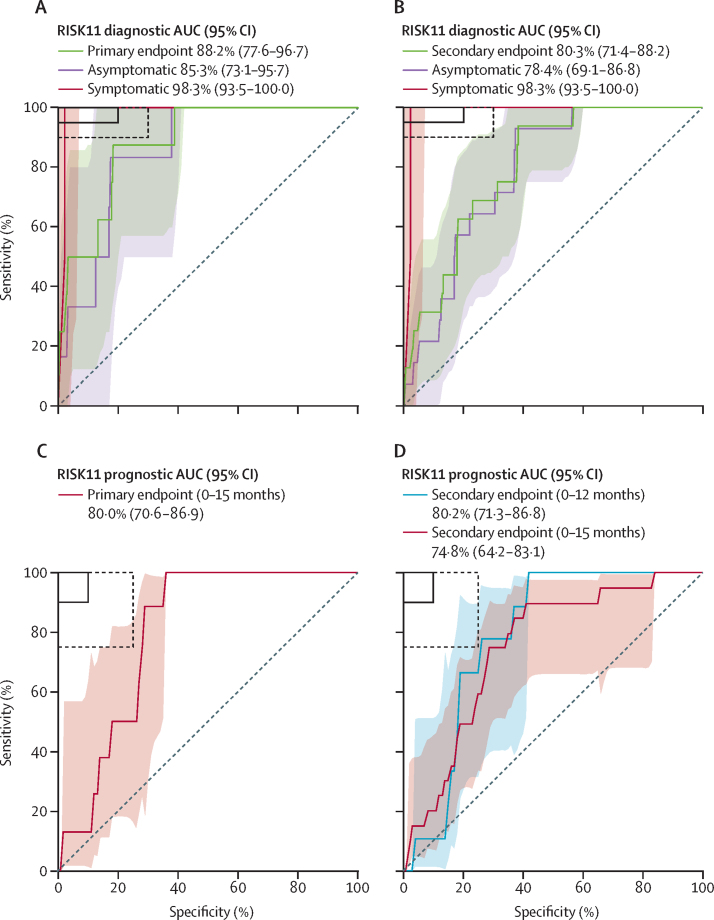
RISK11 diagnostic and prognostic performance for primary and secondary endpoint tuberculosis Receiver operating characteristic curve depicting RISK11 diagnostic performance for the primary (A) and secondary (B) endpoint. The graph shows participants with symptomatic clinical prevalent tuberculosis versus symptomatic controls, and participants with asymptomatic subclinical prevalent tuberculosis versus asymptomatic controls. The shaded areas represent the 95% CIs. The solid box depicts the optimal criteria (95% sensitivity and 80% specificity) and the dashed box depicts the minimal criteria (90% sensitivity and 70% specificity) set out in WHO's target product profile for a triage test.^16^ Receiver operating characteristic curve depicting RISK11 prognostic performance for incident tuberculosis for the primary (C) and secondary (D) tuberculosis endpoints. The shaded areas represent the 95% CIs. The solid box depicts the optimal criteria (90% sensitivity and 90% specificity) and the dashed box depicts the minimal criteria (75% sensitivity and 75% specificity) set out in WHO's target product profile for an incipient tuberculosis test.^17^ AUC=area under the receiver operating characteristic curve.

There were eight participants with primary endpoint prevalent tuberculosis and RISK11 results (seven RISK11-positive and one RISK11-negative), with a prevalence of 2·5% in RISK11-positive participants and 0·2% in RISK11-negative participants (table 1; figure 3A). The RR of primary endpoint prevalent tuberculosis was 13·1 times (likelihood score-based 95% CI 2·1–81·6; χ^2^ p=0·0016) higher in RISK11-positive participants than in RISK11-negative participants (table 2). The RISK11 diagnostic AUC was 88·2% (bootstrap 95% CI 77·6–96·7) for the primary endpoint (table 2; figure 4A). Sensitivity was 87·5% (95% CI 58·3–100·0) and specificity was 65·8% (62·5–69·0; table 2) at the predefined RISK11 score threshold of 60%. The diagnostic performance of RISK11 for the secondary endpoint was similar to that for the primary endpoint (table 2; Figure 3, Figure 4).

**Table 2:** Performance of RISK11, symptom screening, and the LAM assay as triage or diagnostic tests for tuberculosis at baseline

		RISK11 (60)*(n=820)	**Symptom screening (n=861)**	**LAM lateral flow assay (n=858)**
Not tested or inadequate sample		41†	0	3‡
Positive test		285 (35%)	51 (6%)§	14 (2%)¶
Primary endpoint (two or more positive samples)				
	Risk ratio (95% CI; p value)	13·1 (2·1–81·6; p=0·0016)	6·8 (1·9–23·1; p=0·0012)	6·7 (1·1–34·5; p=0·036)
	AUC	88·2% (77·6–96·7)	NA	NA
	Sensitivity	87·5% (58·3–100·0)	30·0% (0·0–62·5)	10·0% (0·0–33·3)
	Specificity	65·8% (62·5–69·0)	94·4% (92·8–95·9)	98·5% (97·6–99·3)
	Positive predictive value‖	2·5% (0·7–4·4)	5·9% (0·0–13·2)	7·1% (0·0–25·0)
	Negative predictive value‖	99·8% (99·4–100·0)	99·1% (98·4–99·8)	98·9% (98·2–99·5)
Secondary endpoint (one or more positive samples)				
	Risk ratio (95% CI; p value)	5·6 (1·9–16·4; p=0·0006)	3·2 (1·0–9·6; p=0·051)	3·5 (0·6–17·1; p=0·18)
	AUC	80·3% (71·4–88·2)	NA	NA
	Sensitivity	75·0% (50·0–94·4)	16·7% (0·0–36·4)	5·6% (0·0–18·8)
	Specificity	66·0% (62·7–69·2)	94·3% (92·8–95·8)	98·5% (97·6–99·3)
	Positive predictive value‖	4·2% (2·0–6·7)	5·9% (0·0–13·2)	7·1% (0·0–25·0)
	Negative predictive value‖	99·3% (98·5–99·8)	98·1% (97·2–99·0)	98·0% (97·0–98·9)

Data are n (%) or % (95% CI), unless otherwise specified. AUC=area under the receiver operating characteristic curve. LAM=lipoarabinomannan. NA=not applicable.

* A priori (60%) RISK11 score threshold.

† Four participants did not have PAXgene RNA samples available and 37 had an indeterminate RISK11 result, probably because of inadequate quality of the RNA sample, and were excluded from the analysis. Two of these participants had prevalent primary endpoint tuberculosis and one had incident primary endpoint tuberculosis.

‡ All three participants missing LAM assay results did not have tuberculosis at baseline.

§ Includes two participants with prevalent primary endpoint tuberculosis who were excluded from RISK11 analysis because of missing results.

¶ Includes one participant with prevalent primary endpoint tuberculosis who was excluded from RISK11 analysis because of missing results.

‖ Computed by use of prevalence in the study population.

Among the 861 enrolled participants, exploratory analysis of tuberculosis symptom screening yielded a sensitivity of 30·0% (95% CI 0·0–62·5) and a specificity of 94·4% (92·8–95·9) for the primary tuberculosis endpoint (table 2). There were 14 positive LAM assays at baseline among 858 enrolled participants (three participants without tuberculosis had missing LAM results), with only one positive assay in a participant with primary endpoint pulmonary tuberculosis (table 2).

Participants with prevalent tuberculosis and who discontinued follow-up at the enrolment visit were excluded from evaluation of prognostic performance (figure 1); 807 participants were followed up for a median of 15 months and evaluated for incident tuberculosis. Of those with RISK11 results, eight people (seven RISK11-positive and one RISK11-negative) had primary endpoint incident tuberculosis (table 1). Primary endpoint tuberculosis incidence in the RISK11-positive group was 2·5 per 100 person-years (Nelson–Aalen 95% CI 0·7–4·4) and in the RISK-negative group was 0·2 per 100 person-years (0·0–0·5; table 1). The probability of primary endpoint incident tuberculosis was 16·0 times (Wald-based 95% CI 2·0–129·5; Wald-based p=0·0092) greater among RISK11-positive participants than among RISK11-negative participants (table 3; figure 3A).

**Table 3:** Prognostic performance of RISK11 and the interferon-γ release assay for incident tuberculosis over 15 months

	RISK11 (60)*	**Interferon-γ release assay**
**Primary endpoint (two or more positive samples)**		
Participants included in analysis	807	802†
Cumulative incidence ratio (95% CI; p value)	16·0 (2·0–129·5; p=0·0092)	2·0 (0·5–8·4;p=0·33)
AUC	80·0% (70·6–86·9)	70·8% (55·0–82·7)
Sensitivity	88·6% (43·5–98·7)	62·1% (25·9–88·5)
Specificity	68·9% (65·3–72·3)	56·2% (52·5–59·9)
Positive predictive value‡	3·2% (1·5–6·6)	1·6% (0·7–3·8)
Negative predictive value‡	99·8% (98·6–100·0)	99·2% (97·6–99·8)
**Secondary endpoint (one or more positive samples)**		
Participants included in analysis	799	794†
Cumulative incidence ratio (95% CI; p value)	6·1 (2·2–16·5; p=0·0004)	2·2 (0·9–5·6; p=0·081)
AUC	74·8% (64·2–83·1)	66·0% (53·8–76·4)
Sensitivity	74·9% (51·3–89·4)	64·8% (42·1–82·4)
Specificity	69·0% (65·5–72·4)	56·5% (52·7–60·2)
Positive predictive value‡	6·5% (4·0–10·6)	4·2% (2·4–7·0)
Negative predictive value‡	99·0% (97·5–99·6)	98·2% (96·3–99·2)

Data are % (95% CI), unless otherwise specified. Performance of RISK11 and the interferon-γ release assay for incident secondary endpoint tuberculosis during 12 months of follow-up can be found in the appendix (p 11). AUC=area under the receiver operating characteristic curve.

* A priori (60%) RISK11 score threshold.

† Five participants without results from the interferon-γ release assay, all tuberculosis-negative, were excluded from the prognostic performance analysis.

‡ Computed by use of incidence in the study population.

We next assessed RISK11 prognostic performance. RISK11 discriminated between primary endpoint incident tuberculosis cases and controls with an AUC of 80·0% (bootstrap 95% CI 70·6–86·9; table 3; figure 4C). At the 60% threshold, sensitivity for incident tuberculosis was 88·6% (95% CI 43·5–98·7) and specificity was 68·9% (65·3–72·3; table 3). By comparison, prognostic sensitivity of the IFN-γ release assay for primary endpoint tuberculosis was 62·1% (95% CI 25·9–88·5) and specificity was 56·2% (52·5–59·9; table 3). No correlation was observed between IFN-γ release assay response and RISK11 score in participants without tuberculosis (Spearman *r*=–0·07; p=0·044; appendix p 15). There was no difference in the risk of incident tuberculosis between IFN-γ release assay-positive and IFN-γ release assay-negative participants (table 3; appendix p 15).

Prognostic performances of RISK11 and the IFN-γ release assay for the secondary endpoint were similar to those for the primary endpoint (table 3; Figure 3, Figure 4; appendix p 15). There were few incident tuberculosis cases before 12 months, precluding analysis of prognostic performance for earlier time windows. Prognostic performance for the secondary endpoint during 12 months was not different to performance during 15 months (appendix p 11; figure 4D). Five participants with negative sputum microbiology on study who were clinically diagnosed with tuberculosis at local clinics and started on empirical tuberculosis therapy were censored from the prognostic performance analysis at the time of last study visit or last negative sputum sample collection; censoring did not qualitatively affect RISK11 prognostic performance (appendix p 12).

To understand factors affecting the RISK11 signature, we used linear regression of RISK11 scores among participants without prevalent tuberculosis (appendix p 13). After correcting for confounders and interaction terms, a multivariable model suggested that older age, male sex, a HIV plasma viral load less than 100 copies per mL, and higher CD4 T-cell counts were independently associated with lower RISK11 scores (appendix p 13). The higher prevalence of isoniazid preventive therapy use among the RISK11-negative group was predominantly due to the association between ART and the provision of isoniazid preventive therapy (data not shown), and ART use was associated with lower RISK11 scores (appendix p 13). In a sensitivity analysis, RISK11's diagnostic and prognostic performance among participants who did not receive isoniazid preventive therapy was similar to performance for the whole cohort (appendix p 14).

## Discussion

We evaluated the diagnostic and prognostic performance of RISK11 in a prospective community screening cohort. This cohort included ART-naive and ART-treated people living with HIV who were not seeking health care and were otherwise healthy with no other important risk factors for tuberculosis, such as a history of tuberculosis or comorbidities such as diabetes. RISK11 identified individuals with pulmonary tuberculosis at screening, and predicted progression to tuberculosis in those without tuberculosis at screening, with performance approaching, but not meeting, the minimum benchmark in WHO's target product profile for tuberculosis triage and prognostic tests.

All participants were intensively screened for tuberculosis at baseline and again at the end of the study with Xpert and mycobacterial culture on spontaneously expectorated sputum samples; 2·1% of participants had previously undiagnosed, microbiologically confirmed pulmonary tuberculosis at baseline. Further, more than 70% of participants with prevalent tuberculosis were asymptomatic, contributing to the low sensitivity of tuberculosis symptom screening. This finding is consistent with the 36–80% prevalence of subclinical tuberculosis found in a review of prevalence surveys,^22^ but higher than the 41% prevalence of asymptomatic tuberculosis reported in a prevalence survey in people living with HIV in South Africa.^23^ LAM assay testing was not useful for intensive case-finding in this community setting, with a sensitivity of 10·0%, consistent with previous evidence.^6^ However, underdiagnosis of extrapulmonary tuberculosis due to a sputum-based tuberculosis endpoint might potentially underestimate the performance of the LAM assay. Additionally, the FujiLAM assay has shown markedly improved performance in outpatient settings compared with the Alere/Abbott TB LAM Ag test.^24^ Asymptomatic participants with tuberculosis in our study would probably have been missed by symptom screening strategies, leading to delayed presentation, increased morbidity, and potential transmission to close contacts.^25^ This finding supports the development of better tests to screen for tuberculosis and find these so-called missing millions. However, the clinical significance of asymptomatic microbiologically confirmed tuberculosis is not clear and some of these individuals might have contained the disease, or even self-cured without treatment.^26^

Over the 15-month follow-up, nine participants (1·0 case per 100 person-years) progressed to microbiologically confirmed, primary endpoint incident pulmonary tuberculosis and 21 participants (2·3 cases per 100 person-years) progressed to secondary endpoint disease. Latent *M tuberculosis* infection, defined by a positive IFN-γ release assay or tuberculin skin test, includes a spectrum that spans immunological control and even clearance of infection through to incipient asymptomatic disease.^27^ We observed no correlation between IFN-γ release assay response and RISK11 score, which is not surprising given that the IFN-γ release assay measures *M tuberculosis*-specific T-cell responses and RISK11 measures a completely different immune response—namely, expression of IFN-stimulated genes predominantly in innate leukocytes.^12^ We found that, at the manufacturer-recommended threshold for latent infection (≥0·35 IU/mL), the IFN-γ release assay was not able to differentiate between people living with HIV who had been exposed to *M tuberculosis* but were able to control infection, from those who will progress to disease over 15 months, which might result in either unnecessary treatment or missed opportunities to prevent progression to disease. By contrast, the probability of primary endpoint incident tuberculosis was 16·0-times higher in RISK11-positive participants than in RISK11-negative participants during 15 months of follow-up. The 15-month prognostic window is encouraging given the heterogeneous nature of progression to tuberculosis disease and findings from a meta-analysis of transcriptomic signatures, which were only able to predict risk of tuberculosis disease in people without HIV up to 6 months before diagnosis.^11^ RISK11 performed as well in people with HIV as in an intensified case-finding study^14^ of people without HIV. This result is surprising because HIV, and in particular high HIV load, is known to induce the expression of IFN-stimulated genes and thus results in higher RISK11 signature scores, potentially confounding the test result.^15^ However, Turner and colleagues^10^ found no difference in the diagnostic accuracy of blood transcriptomic signatures by HIV status in a cohort of adults presenting with symptoms of tuberculosis. Higher median RISK11 scores in participants with HIV plasma viral loads of 100 copies per mL or more than in those with viral loads less than 100 copies per mL suggest that RISK11 specificity might be reduced in an exclusively ART-naive population, and repeat testing following ART initiation or alternative management pathways might be required. Transcriptomic signatures that are not considerably affected by HIV-associated induction of IFN-stimulated genes might perform better in this group.^28^ The finding of lower RISK11 scores in men and older people is in keeping with evidence that IFN-stimulated gene expression is linked to sex^29^ and age.^30^

Current South African guidelines advocate 12 months of universal isoniazid preventive therapy for people living with HIV who have not yet received tuberculosis preventive therapy, irrespective of tuberculin skin testing or IFN-γ release assay status.^31^ This study suggests that a transcriptomic signature of tuberculosis risk, such as RISK11, might be more specific in determining need for targeted preventive therapy for people living with HIV. Two-thirds of the 38 million people living with HIV worldwide are on ART^32^ and, with the advent of well tolerated and effective short-course tuberculosis preventive regimens,^33^, ^34^ annual or semi-annual transcriptomic community-based testing of people living with HIV might be useful to monitor risk of progression to tuberculosis and target those likely to benefit from repeat courses of preventive therapy.

Although RISK11, which comprises 48 primer-probe gene expression assays, is not sufficiently parsimonious to be implemented as a rapid point-of-care test, this study offers proof of concept that a biomarker-guided, community-based tuberculosis screening strategy might be feasible for people living with HIV. A further refined, more concise transcriptomic signature, such as RISK6,^35^ might be adapted into a point-of-care device to guide confirmatory investigation for tuberculosis disease and initiation of preventive therapy. However, it is not yet known whether such concise transcriptomic signatures, previously validated in carefully curated case-control studies using RNA sequencing or microarrays, will show similar performance in prospective, diagnostic accuracy studies using near-the-point-of-care or point-of-care technologies, such as isothermal PCR. A head-to-head analysis of eight concise transcriptomic signatures adapted to real-time PCR is also underway using RNA samples from this study and from people without HIV in the CORTIS-01 study.^14^ We also plan to evaluate the performance of proteomic and metabolomic biomarkers, such as C-reactive protein, in these cohorts. Despite RISK11's promising prognostic performance in this study, whether transcriptomic biomarker-targeted preventive therapy would be affordable and efficacious in preventing progression to clinical tuberculosis disease in people with HIV remains to be seen.

Our study has several limitations. First, reliance on sputum Xpert and liquid culture as the microbiological reference standard precludes evaluation of test performance for extrapulmonary tuberculosis, which is more common in people with HIV than in people without HIV. 14 participants had positive LAM assay results, of whom only one was sputum *M tuberculosis*-positive and treated with anti-tuberculosis therapy; extrapulmonary disease cannot be ruled out in the remainder. Second, spontaneous spot sputum sample collection might have missed early or paucibacillary disease, which might have been detected by induced sputum, bronchoalveolar lavage, or by radiological imaging. Third, alternative diagnoses were not systematically ascertained for symptomatic individuals without microbiologically confirmed tuberculosis. Fourth, although the regression analysis did not show a significant association between isoniazid preventive therapy and RISK11 score at enrolment, a greater proportion of RISK11-negative participants than RISK-positive participants received isoniazid preventive therapy during the study; therefore, the lower tuberculosis incidence in RISK11-negative participants might be in part due to isoniazid preventive therapy. However, we showed that RISK11's diagnostic and prognostic performance among participants who did not receive isoniazid preventive therapy was similar to RISK11's performance for the whole cohort. Fifth, more participants in the RISK11-positive group than in the RISK11-negative group discontinued the study early; participants in the RISK11-positive group had a lower median body-mass index, were more likely to be ART-naive and symptomatic, and had higher RISK11 scores than participants in the RISK11-negative group. We can surmise that participants in the RISK11-positive group might have had more advanced HIV and some might represent missed tuberculosis cases, potentially resulting in an underestimation of RISK11 performance. Finally, although people with HIV are at high risk for tuberculosis and the study sites in South Africa were selected because of their high tuberculosis burden, the small number of primary endpoint tuberculosis cases limited the precision of diagnostic and prognostic estimates. However, RISK11's diagnostic and prognostic performance was still significantly greater than its performance by chance. Case numbers were too low to study the effect of ART, isoniazid preventive therapy, CD4 cell count, HIV plasma viral load, IFN-γ release assay status, tuberculosis symptoms, and other clinical variables on RISK11 performance. Further studies are needed to evaluate performance in adults older than 60 years, individuals with comorbidities, such as diabetes and recent tuberculosis, or household contact with tuberculosis, and children. We were also not able to study prognostic performance for time windows shorter than 12 months. RISK11 positive predictive values for primary endpoint prevalent (2·5%) and incident disease (3·2%) were computed with the observed tuberculosis prevalence (1%) and incidence rates (0·9% annually), which, in this ambulatory community setting, were lower than that typically used in estimations (2%).^16^, ^17^, ^36^ Whether these results are generalisable to other geographical or clinical settings, such as people with HIV seeking health care or who are hospitalised with advanced HIV and low CD4 cell counts, remains to be seen.

Prospective field validation and implementation studies of rapid point-of-care real-time PCR devices that measure concise transcriptomic signatures are now needed to test the efficacy of biomarker-driven screening strategies to guide tuberculosis confirmatory testing and targeted short-course preventive therapy in HIV-affected communities with endemic tuberculosis.

## Data sharing

Deidentified RISK11 signature scores, PCR probe data, clinical metadata, and tuberculosis endpoint data will be made available with publication. The dataset has been deposited in Zivahub (https://doi.org/10.25375/uct.14176484; under embargo until July 1, 2021), an open access data repository hosted by the University of Cape Town's institutional data repository powered by Figshare for Institutions.

## Declaration of interests

AP-N, GW, GC, TJS, and MH report grants from the Bill & Melinda Gates Foundation during the conduct of the study. AP-N and GW report grants from the South African Medical Research Council during the conduct of the study. GW and TJS report grants from the South African National Research Foundation during the conduct of the study. AP-N and TJS have patents of the RISK11 and RISK6 signatures pending. GW has had a patent (tuberculosis diagnostic markers; PCT/IB2013/054377) issued and a patent (method for diagnosing tuberculosis; PCT/IB2017/052142) pending. All other authors declare no competing interests.
