# Supplementary material for: Validation of a host blood transcriptomic biomarker for pulmonary tuberculosis in people living with HIV: a prospective diagnostic and prognostic accuracy study
Source: Lancet Glob Health. 2021 Apr 13;9(6):e841–53. doi: 10.1016/S2214-109X(21)00045-0 (PMC8131200; doi:10.1016/S2214-109X(21)00045-0)
Supplement: Supplementary appendix [file mmc1.pdf]

THE LANCET
Global Health

Supplementary appendix

This appendix formed part of the original submission and has been peer reviewed. We post it as supplied by the authors.

Supplement to: Mendelsohn SC, Fiore-GartlandA, Penn-Nicholson A, et al. Validation of a host blood transcriptomic biomarker for pulmonary tuberculosis in people living with HIV: a prospective diagnostic and prognostic accuracy study. *Lancet Glob Health* 2021; published online April 13. https://doi.org/10.1016/S2214-109X(21)00045-0.

Appendix to:

Validation of a host blood transcriptomic biomarker for pulmonary tuberculosis in people living with HIV: a prospective diagnostic and prognostic accuracy study

Simon C. Mendelsohn, MBChB[1], Andrew Fiore-Gartland, PhD[2], Adam Penn-Nicholson, PhD[1], Humphrey Mulenga, MPH[1], Stanley Kimbung Mbandi, PhD[1], Bhavesh Borate, MS[2], Katie Hadley, PhD[1], Chris Hikuam, PhD[1], Munyaradzi Musvosvi, PhD[1], Nicole Bilek, PhD[1], Mzwandile Erasmus, BSc[1], Lungisa Jaxa, BTech[1], Rodney Raphela, BSc[1], Onke Nombida, GT[1], Masooda Kaskar, MBChB[1], Tom Sumner, PhD[3], Richard G. White, PhD[3], Craig Innes, MBChB[4], William Brumskine, MBChB[4], Andriëtte Hiemstra, MBChB[5], Stephanus T. Malherbe, MBChB[5], Razia Hassan-Moosa, MPH[6,7], Michèle Tameris, MBChB[1], Gerhard Walzl, MBChB[5], Kogieleum Naidoo, PhD[6,7], Gavin Churchyard, PhD[4,8], Thomas J. Scriba, DPhil[1*], Mark Hatherill, MD[1*] and the CORTIS-HR Study Team.

Contents

The CORTIS-HR Study Team Page 2

Supplementary Methods Page 5

Supplementary Tables Page 8

Table S1: RISK11 signature primer-probe assay panel Page 8

Table S2: Baseline characteristics and RISK11 status of study cohort by clinical site in all enrolled participants Page 9

Table S3: Baseline characteristics and RISK11 status of study cohort by censoring status Page 10

Table S4: Prognostic performance of RISK11 and interferon-γ release assay for incident microbiologically-confirmed tuberculosis through 12 months of follow-up Page 11

Table S5: Sensitivity analysis of RISK11 performance including participants with negative sputum microbiology who were clinically diagnosed with tuberculosis at local clinics and started on empiric tuberculosis therapy during the conduct of the study as tuberculosis cases Page 12

Table S6: Linear regression analysis examining factors associated with RISK11 score in participants without tuberculosis disease at baseline Page 13

Table S7: Sensitivity analysis of RISK11 performance excluding participants who received Isoniazid preventive therapy during the conduct of the study Page 14

Supplementary Figures Page 15

Appendix 1: Study Protocol: Validation of correlates of risk of TB disease in high risk populations (CORTIS-HR): A Companion Study of the CORTIS-01 Trial (Protocol version 1.0 dated 26th August 2016) Page 16

Appendix 2: Statistical Analysis Plan (Version 1.0 dated 11th December 2019) Page 43

Appendix 3: STARD 2015 Checklist: An updated list of essential items for reporting diagnostic accuracy studies Page 87

The CORTIS-HR Study Team

[1]South African Tuberculosis Vaccine Initiative, Institute of Infectious Disease and Molecular Medicine, Division of Immunology, Department of Pathology, University of Cape Town, South Africa.
Charmaine Abrahams
Hadn Africa
Denis Arendsen
Nomfuneko Cynthia Batyi
Samentra Braaf
Sivuyile Buhlungu
Alida Carstens
Yolundi Cloete
Lorraine Coetzee
Alessandro Companie
Ilse Davids
Marwou de Kock
Bongani Diamond
Palesa Dolo
Juanita Ferreira
Christal Ferus
Michelle Fisher
Hennie Geldenhuys
Diann Gempies
Yolande Gregg
Rieyaat Hassiem
Roxane Herling
Yulandi Herselman
Henry Issel
Ruwiyda Jansen
Fabio Julies
Fazlin Kafaar
Sophie Keffers
Xoliswa Kelepu
Gloria Khomba
Sandra Kruger
Sunelza Lakay
Thelma Leopeng
Angelique Kany Kany Luabeya
Simbarashe Mabwe
Lebohang Makhethe
Faheema Meyer
Miriam Moses
Boitumelo Mosito
Angelique Mouton
Julia Noble
Fajwa Opperman
Christel Petersen
Patiswa Plaatjie
Abe Pretorius
Frances Ratangee
Maigan Ratangee
Elisma Schoeman
Constance Schreuder
Alison September
Cashwin September
Justin Shenje
Marcia Steyn
Sonia Stryers
Liticia Swanepoel
Anne Swarts
Asma Toefy
Petrus Tyambetyu
Linda van der Merwe
Elma van Rooyen
Habibullah Valley
Ashley Veldsman
Helen Veldtsman
Kelvin Vollenhoven
Elaine Zimri

[2]Vaccine and Infectious Disease Division, Fred Hutchinson Cancer Research Center, Seattle, WA, USA.
Eva Chung
Michelle Chung
Alicia Sato
Ellis Hughes

[3]TB Modelling Group, TB Centre, Centre for Mathematical Modelling of Infectious Diseases, Department of Infectious Disease Epidemiology, London School of Hygiene & Tropical Medicine, London, United Kingdom.

[4]The Aurum Institute, Johannesburg, Gauteng, South Africa.
Aurum Klerksdorp Site
Tebogo Badimo
Kagiso Baepanye
Kesenogile Edna Baepanye
Ken Clarke
Marelize Collignon
Audrey Lebohang Dhlamini
Candice Eyre
Tebogo Feni
Welseh Phindile Galane
Thelma Goliath
Bonita Janse Van Rensburg
Olebogeng Jonkane
Boitumelo Sophy Kekana
Gomotsegang Virginia Khobedi
Marietjie King
Ndlela Israel Kunene
Aneesa Lakhi
Nondumiso Langa
Hildah Ledwaba
Immaculate Mabasa
Tshegofatso Dorah Mabe
Nkosinathi Charles Mabuza
Molly Majola
Mantai Makhetha
Vernon Malay
Juanita Market
Lungile Mbata
Karabo Moche
Joseph Panie Moloko
Kabelo Molosi
Primrose Mothaga
Nhlamulo Ndlovu
Bantubonke Bertrum Ntamo
Tedrius Ntshauba
Thandiwe Papalagae
Pearl Nomsa Sanyaka

Letlhogonolo Seabela
Raesibe Agnes Pearl Selepe
Melissa Neo Senne
Moeti Serake
Mugwena Thompo
Vincent Tshikovhi
Lebogang Isaac Tswaile

Aurum Rustenberg Site
Laudicia Tshenolo Bontsi
Obakeng Peter Booi
Mari Cathrin Botha
Kgomotso Violet Chauke
Mooketsi Theophillius Cwaile
Isabella Johanna Davies
Emilia De Klerk
Blanchard Mbay Iyemosolo
James Michael Jeleni
Christian Mabika Kasongo
Christian Mabika Kasongo
Sebaetseng Jeanette Kekana
Lucky Sipho Khoza
Gloria Keitumetse Kolobe
Lerato Julia Lekagane
Sheiley Christina Lekotloane
Ilze Jeanette Louw
Sarah Teboso Lusale
Perfect Tiisetso Maatjie
Kamogelo Fortunate Mabena
Johanna Thapelo Madikwe
Octavia Mahkosazana Madikwe
Rapontwana Letlhogonolo Maebana
Malobisa Sylvester Magwasha
Vutlhari-I-Vunhenha Fairlord Manzini
Isholedi Samuel Maroele
Omphile Petunia Masibi
July Rocky Mathabanzini
Tendamudzimu Ivan Mathode
Ellen Ditaba Matsane
Lungile Mbata
Nyasha Karen Mhandire
Thembisiwe Miga
Nosisa Charity Thandeka Mkhize
Caroline Mkhokho
Neo Hilda Mkwalase
Nondzakazi Mnqonywa
Brenda Matshidiso Modisaotsile
Patricia Pakiso Mokgetsengoane
Selemeng Matseliso Carol Mokone
Kegomoditswe Magdeline Molatlhegi
Thuso Andrew Molefe
Motlatsi Evelyn Molotsi
Tebogo Edwin Montwedi
Boikanyo Dinah Monyemangene
Hellen Mokopi Mooketsi
Tshplpfelo Mapula Mosito
Ireen Lesebang Mosweu
Banyana Olga Motlagomang
Funeka Nomvula Mthembu
Themba Phakathi
Mapule Ozma Phatshwane
Victor Kgothatso Rameetse
Kelebogile Magdeline Segaetsho
Ni Ni Sein
Melissa Neo Senne
Sifiso Cornelius Shezi
Zona Sithetho
Bongiwe Stofile
Mando Mmakhora Thaba
Lethabo Collen Theko
Dimakatso Sylvia Tsagae

[5]DST/NRF Centre of Excellence for Biomedical TB Research and SAMRC Centre for TB Research, Division of Molecular Biology and Human Genetics, Department of Biomedical Sciences, Faculty of Medicine and Health Sciences, Stellenbosch University, Cape Town, South Africa.
Petri Ahlers
Roslyn Beukes
Maria Didloff
Marika Flinn
Bernadine Fransman
Jaftha Kruger
Onesisa Mpofu
Mpho Mtlali
Dorothy Solomons
Kim Stanley
Susanne Tönsing
Khayalethu Toto
Ayanda Tsamane

[6]Centre for the AIDS Programme of Research in South Africa (CAPRISA), and [7]MRC-CAPRISA HIV-TB Pathogenesis and Treatment Research Unit, Doris Duke Medical Research Institute, University of KwaZulu-Natal, Durban, South Africa.
Bianca Bande
Thilagavathy Chinappa
Cara-mia Corris
Thobelani Cwele
Celaphiwe Dlamini
Dhineshree Govender
Goodness Gumede
Zanele Gwamada
Senzo Halti
Zandile Jali
Lungile Khanyile
Jabu Maphanga
Nonhle Maphanga
Nompumelelo Ngcobo
Gloria Ntanjana
Nesri Padayatchi
Dirhona Ramjit
Thandiwe Shezi
Chandrapharbha Singh
Phindile Sing
Philile Thembela
Londiwe Zaca
Mbali Zulu

[8]School of Public Health, University of Witwatersrand, Johannesburg, South Africa.

Supplementary Methods

Diagnostic testing

Sputum liquid mycobacterial culture (MGIT, BACTEC, Beckton Dickinson, Franklin Lakes, USA), Xpert MTB/RIF or Xpert Ultra (Cepheid, Sunnyvale, USA), lateral flow lipoarabinomannan assay (Determine TB-LAM, Alere, Waltham, USA), HIV plasma viral load, and CD4 cell count measurement were performed at accredited diagnostic laboratories: the Bio Analytical Research Corporation South Africa (BARC SA) and the South African National Health Laboratory Service (NHLS). IGRA (QuantiFERON TB Gold-Plus, Qiagen, Hilden, Germany) was performed on all samples at the South African Tuberculosis Vaccine Initiative (SATVI) Laboratory in Cape Town.

RISK11 assay

Venous blood was collected in PAXgene RNA tubes (PreAnalytiX, Hombrechtikon, Switzerland) from all potentially eligible participants at enrolment, frozen at -20°C, and shipped to the SATVI Laboratory in Cape Town. The 11-gene RISK11 host-response tuberculosis transcriptomic signature was measured as previously described.[1-3] In brief, PAXgene tubes were thawed in batches of 93 (plus 3 internal assay controls), allowing for standard 96-well plate format handling. RNA was extracted with the Maxwell SimplyRNA custom kit (Promega, Madison WI, USA) by an automated Freedom EVO 150 robotic platform (Tecan, Durham NC, USA) and cDNA synthesised using EpiScript reverse transcriptase (Lucigen, Middleton WI, USA) immediately post extraction. An aliquot of cDNA was pre-amplified with a pool of 48 TaqMan primer-probe assays (Thermo Fisher Scientific, Waltham MA, USA), comprising 38 transcripts of interest and 10 reference transcripts for normalization that constitute the 11-gene RISK11 signature (Table S1). We have previously shown that the pared-down 11-gene RISK11 signature has equivalent performance to the original 16-gene Zak signature[4] (47 transcripts of interest; 10 housekeeping transcripts).[1] Pre-amplified cDNA from the 93 samples, along with an internal positive control, a no-template (water) control, and a no-reverse-transcriptase control, and the 48 primer-probe assays, were loaded into a microfluidic 96.96 Gene Expression Integrated Fluidic Circuit (Fluidigm, San Francisco CA, USA) and qRT-PCR performed on the Fluidigm BioMark HD instrument.

Quality control (including reproducibility against an internal positive control sample), analysis of gene expression cycle threshold data, and computation of the RISK11 score was performed using a locked down R script (available on Bitbucket at https://bitbucket.org/satvi/cortis). Samples with failed reference primer-probe reactions, with marked deviation in internal positive control sample from historical runs, or with more than 30% failed primer-probe reactions for transcript of interest (see QC criteria and analysis script in Bitbucket instance) were classified as indeterminate. Participants with missing (test not performed) or indeterminate RISK11 scores were excluded from analysis.

Statistical analysis

Study design and sample size calculations are detailed in the study protocol (Appendix 1). All analyses were pre-specified in the statistical analysis plan (Appendix 2). Statistical analyses were performed in RStudio version 1.2.5001 (RStudio PBC, Boston MA, USA). An alpha of <0·05 was considered statistically significant in all analyses.

Endpoint adjudication

A case adjudication algorithm for identifying tuberculosis disease endpoints was pre-specified in the statistical analysis plan (Appendix 2). Briefly, two or more independent microbiologically-confirmed (Xpert MTB/RIF, Ultra, or MGIT culture) sputum samples for *Mycobacterium tuberculosis* (≥2 sample+) within a 30-day window was considered a primary endpoint. The date of sample collection of the first positive sputum was considered the endpoint date.

A single positive sputum sample within a 30-day window was considered a secondary tuberculosis endpoint (≥1 sample+). If there was a subsequent primary endpoint (episode 2) following a secondary endpoint (episode 1), the double-positive sputum sample (episode 2) would also be considered as the secondary endpoint episode. To obtain a more stable estimate of RISK11 prognostic performance metrics at month 15 (day 449), all endpoints and censoring events between 14·5 - 15 months were "rounded" to 15 months. Participants with follow-up beyond 15·5 months were censored at 15 months. There were no tuberculosis endpoints beyond 15·5 months.

All analyses were pre-specified in the statistical analysis plan (Appendix 2). Participants with a tuberculosis endpoint at the enrolment visit were classified as prevalent tuberculosis cases. Participants with a tuberculosis endpoint at a subsequent visit were defined as incident tuberculosis disease cases. Individuals who remained tuberculosis free until study discontinuation, or end of follow up at the month 15 end of study visit were classified as tuberculosis negative. Participants with only a single sputum sample positive for *Mycobacterium tuberculosis* were classified as a tuberculosis case using the secondary tuberculosis endpoint definition, and as tuberculosis negative using the primary endpoint definition.

Descriptive analysis

Descriptive analysis p-values were calculated from Wilcoxon Rank Sum test (continuous data) or Pearson's Chi-squared test (categorical data). P-values for comparison of median RISK11 signature scores between groups in box-and-whisker plots were calculated with the Wilcoxon Rank Sum test and corrected for multiple comparisons using the Benjamini-

Hochberg Procedure[5]. The Spearman's rank-order correlation coefficient (ρ) was used to measures the strength and direction of association between two non-parametric ranked variables. Locally estimated scatterplot smoothing (LOESS) curves with 95% confidence interval were overlaid on scatterplots of RISK11 signature score versus QuantiFERON-TB Gold Plus (QFT) interferon-γ (IFN- γ) response to represent local polynomial regression.

Tuberculosis prevalence
The risk of prevalent tuberculosis (prevalence, %) was estimated as the proportion of participants with tuberculosis at enrolment. The 95% confidence intervals on risk of tuberculosis (prevalence) was calculated using the Binomial Wilson method using the R *binom* package.

Diagnostic performance
The primary analysis of RISK11 performance estimated the relative risk of developing endpoint-defined tuberculosis disease. Participants with a RISK11 score ≥ 60% were classified *a-priori* as RISK11+ and < 60% as RISK11-. The relative risk of prevalent disease was computed as the risk of prevalent tuberculosis in RISK11+ divided by the risk of prevalent tuberculosis in RISK11-. The 95% confidence intervals on relative risk were calculated with a likelihood-score-based approach[6,7] using the R *riskscoreci* function in the *PropCIs* package, and p value using a Chi-squared test.

Binary receiver operating characteristic (ROC) curve analysis was performed using the R *pROC* package[8] to calculate diagnostic area under the ROC curve (AUC). Diagnostic metrics including; sensitivity, specificity, positive predictive value (PPV), and negative predictive value (NPV) were calculated using binary endpoint indicators and standard formulas. The 95% confidence intervals on diagnostic performance metrics for prevalent tuberculosis was calculated using non-stratified, non-parametric percentile bootstrap[9] with 10,000 iterations.

Identical analytical methods were applied to analysis of baseline urine Alere Determine TB-lipoarabinomannan (LAM) lateral flow assay and tuberculosis symptom screening diagnostic performance in the whole enrolled cohort (N=861 participants). Individuals with missing LAM (n=3) results were excluded from analysis of LAM diagnostic performance (N=858).

Prognostic performance
The incidence (per 100 person-years) and cumulative incidence (probability, %) of tuberculosis through 15 months was estimated among participants using a time-dependent right-censored non-parametric approach (estimate and variance derived from the Nelson-Aalen estimator[10] of cumulative hazard). Participants with prevalent tuberculosis at enrolment, or who only attended the initial enrolment visit (follow-up time = 0), were excluded from this estimate. The relative risk of incident disease (referred to as the cumulative incidence ratio) was computed as the cumulative incidence of tuberculosis in RISK11+ divided by the cumulative incidence of tuberculosis in RISK11-. The point-estimate for the cumulative incidence ratio or relative risk (RR) for incident tuberculosis through 15 months [$RR_{RISK11}(15)$] are presented with 95% confidence intervals and a p-value for the null-hypothesis H_0: $RR_{RISK11}(15) \leq 1$ using a Wald-based approach.

A time-dependent right-censored non-parametric, inverse-probability weighted approach to ROC analysis from survival data was performed using the R *survAM.estimate* function in the *survAccuracyMeasures* package[11] to calculate prognostic AUC, sensitivity, specificity, PPV, and NPV for incident tuberculosis through 15 months follow up, with 95% confidence intervals calculated using bootstrapping with 10,000 iterations. Estimates of PPV and NPV were computed based on the observed incidence of tuberculosis in the study population. Analyses of prognostic performance using censored follow-up time windows were performed using the same methods.

Identical analytical methods were applied to analysis of IGRA prognostic performance. Individuals with missing IGRA (n=5) results were excluded from analysis (N=802 for primary endpoint; N=794 for secondary endpoint).

Linear regression analysis
Univariable linear regression models were built using RISK11 signature score as the dependent variable in non-tuberculosis controls to assess the effect of participant characteristics on RISK11 score. Collinear predictors (such as BMI) were excluded. Sex, age, and covariables which significantly (p-value < 0·05) predicted RISK11 signature score in the univariable linear models were added to a multivariable linear regression model of RISK11 signature score. Performance of the model was limited due to missingness of variable observations (HIV plasma viral load and IGRA result).

Unblinded interim diagnostic analysis
A RISK11-unblinded interim analysis of RISK11 diagnostic performance was performed by the unblinded statistician at SCHARP after all participants completed tuberculosis evaluation for the enrolment visit. For the analysis, the statisticians were unblinded to RISK11 status and tuberculosis endpoint status at enrolment. Based on the projected number of incident endpoints and expected power, the study steering committee recommended that the study should continue and evaluate RISK11 prognostic performance.

References

1. Darboe F, Mbandi SK, Thompson EG, Fisher M, Rodo M, van Rooyen M, et al. Diagnostic performance of an optimized transcriptomic signature of risk of tuberculosis in cryopreserved peripheral blood mononuclear cells. Tuberculosis (Edinb). 2018;108:124-6. doi: 10.1016/j.tube.2017.11.001.
2. Darboe F, Mbandi SK, Naidoo K, Yende-Zuma N, Lewis L, Thompson EG, et al. Detection of Tuberculosis Recurrence, Diagnosis and Treatment Response by a Blood Transcriptomic Risk Signature in HIV-Infected Persons on Antiretroviral Therapy. Front Microbiol. 2019;10:1441. doi: 10.3389/fmicb.2019.01441.
3. Scriba TJ, Fiore-Gartland A, Penn-Nicholson A, Mulenga H, Kimbung Mbandi S, Borate B, et al. Biomarker-guided tuberculosis preventive therapy (CORTIS): a randomised controlled trial. The Lancet Infectious Diseases. 2021;21(3):354-65. doi: 10.1016/s1473-3099(20)30914-2.
4. Zak DE, Penn-Nicholson A, Scriba TJ, Thompson E, Suliman S, Amon LM, et al. A blood RNA signature for tuberculosis disease risk: a prospective cohort study. Lancet. 2016;387(10035):2312-22. doi: 10.1016/S0140-6736(15)01316-1.
5. Benjamini Y, Hochberg Y. Controlling the false discovery rate: a practical and powerful approach to multiple testing. Journal of the Royal statistical society: series B (Methodological). 1995;57(1):289-300.
6. Koopman P. Confidence intervals for the ratio of two binomial proportions. Biometrics. 1984:513-7.
7. Nam JM. Confidence limits for the ratio of two binomial proportions based on likelihood scores: non-iterative method. Biometrical Journal. 1995;37(3):375-9.
8. Robin X, Turck N, Hainard A, Tiberti N, Lisacek F, Sanchez JC, et al. pROC: an open-source package for R and S+ to analyze and compare ROC curves. BMC Bioinformatics. 2011;12:77. doi: 10.1186/1471-2105-12-77.
9. Carpenter J, Bithell J. Bootstrap confidence intervals: when, which, what? A practical guide for medical statisticians. Statistics in Medicine. 2000;19(9):1141-64. doi: 10.1002/(sici)1097-0258(20000515)19:9<1141::Aid-sim479>3.0.Co;2-f.
10. Aalen O. Nonparametric inference for a family of counting processes. The Annals of Statistics. 1978:701-26.
11. Zheng Y, Cai T, Brown M. Package 'survAccuracyMeasures'. 2014. http://mdbrown.github.io/survAccuracyMeasures/ (accessed May 22, 2020).

Supplementary Tables

Table S1: RISK11 signature primer-probe assay panel

Gene Symbol	TaqMan Assay ID	Reference Gene or Gene of Interest	Sequence Accession Number for Custom Assays	Context Sequence for Custom Assays
ACTR3	Hs01029159_g1	Reference Gene		
ADRBK1	Hs01056345_g1	Reference Gene		
CDC42	Hs03044122_g1	Reference Gene		
CSDE1	Hs00918650_m1	Reference Gene		
CYTIP	Hs00188734_m1	Reference Gene		
TMBIM6	Hs00162661_m1	Reference Gene		
TMBIM6	Hs01012081_m1	Reference Gene		
TMBIM6	Hs01012082_g1	Reference Gene		
TPM3	Hs01900726_g1	Reference Gene		
USF2	Hs01100994_g1	Reference Gene		
BATF2	Hs00912736_m1	Gene of Interest		
ETV7	Hs00903228_m1	Gene of Interest		
ETV7	Hs00903230_g1	Gene of Interest		
ETV7	ETV7-j2 (custom assay)	Gene of Interest	NM_001207035	ACTCCTGTTCAGAAATGGACTTCAG
FCGR1C	Hs00417598_m1	Gene of Interest		
GBP1	Hs00266717_m1	Gene of Interest		
GBP1	Hs00977005_m1	Gene of Interest		
GBP1	GBP1-j1 (custom assay)	Gene of Interest	NM_002053	AAGAAAGGTACCAGTCAAAAAGATG
GBP2	Hs00894837_m1	Gene of Interest		
GBP2	Hs00894840_mH	Gene of Interest		
GBP2	Hs00894842_g1	Gene of Interest		
GBP2	Hs00894846_g1	Gene of Interest		
GBP2	GBP2-j1 (custom assay)	Gene of Interest	NM_004120	AGAGGAAATTAGGGGCCCAGTTGGA
GBP5	Hs00369472_m1	Gene of Interest		
GBP5	GBP5-j4 (custom assay)	Gene of Interest	NM_052942	ACAGATGCAGGAACAGGCTGCACAG
SCARF1	Hs00186503_m1	Gene of Interest		
SCARF1	Hs01092482_g1	Gene of Interest		
SCARF1	Hs01092483_m1	Gene of Interest		
SCARF1	Hs01092485_g1	Gene of Interest		
SERPING1	Hs00163781_m1	Gene of Interest		
SERPING1	Hs00934328_g1	Gene of Interest		
SERPING1	Hs00934329_m1	Gene of Interest		
SERPING1	Hs00934330_m1	Gene of Interest		
SERPING1	Hs00935959_m1	Gene of Interest		
STAT1	Hs01013989_m1	Gene of Interest		
STAT1	Hs01013991_m1	Gene of Interest		
STAT1	Hs01013992_g1	Gene of Interest		
STAT1	Hs01013993_m1	Gene of Interest		
STAT1	Hs01013994_m1	Gene of Interest		
STAT1	Hs01013995_g1	Gene of Interest		
STAT1	Hs01013996_m1	Gene of Interest		
STAT1	Hs01013997_m1	Gene of Interest		
STAT1	Hs01013998_m1	Gene of Interest		
STAT1	Hs01014000_m1	Gene of Interest		
STAT1	Hs01014002_m1	Gene of Interest		
TAP1	Hs00388675_m1	Gene of Interest		
TAP1	Hs00897093_g1	Gene of Interest		
TRAFD1	Hs00938765_m1	Gene of Interest		

Table S2: Baseline characteristics and RISK11 status of study cohort by clinical site in all enrolled participants

		Overall N=861	Durban N=300	Klerksdorp N=160	Rustenburg N=161	Ravensmead N=90	Worcester N=150
**Baseline characteristics at study enrolment**							
Female sex, n (%)		621 (72·1)	217 (72·3)	102 (63·7)	113 (70·2)	66 (73·3)	123 (82·0)
Median age, years (IQR)		35 (29-42)	35 (29-42)	32 (27-38)	34 (29-40)	38 (30-46)	38 (31-44)
Ethnicity, n (%)							
Black African		724 (84·1)	299 (99·7)	159 (99·4)	160 (99·4)	3 (3·3)	103 (68·7)
Mixed Ancestry		137 (15·9)	1 (0·3)	1 (0·6)	1 (0·6)	87 (96·7)	47 (31·3)
Median BMI, kg/m^2 (IQR)		24·2 (20·6-31·2)	25·9 (21·7-32·3)	22·6 (19·3-29·7)	23·8 (20·3-29·8)	20·7 (18·8-27·2)	26·2 (21·9-32·2)
Smoking history, n (%)		334 (38·8)	92 (30·7)	61 (38·1)	43 (26·7)	69 (76·7)	69 (46·0)
Prior tuberculosis, n (%)		212 (24·6)	73 (24·3)	31 (19·4)	25 (15·5)	32 (35·6)	51 (34·0)
Tuberculosis household contacts, n (%)		160 (18·6)	77 (25·7)	21 (13·1)	23 (14·3)	30 (33·3)	9 (6·0)
IGRA result, n (%)							
Not available		7 (0·8)	4 (1·3)	0	3 (1·9)	0	0
Negative		461 (53·5)	167 (55·7)	69 (43·1)	107 (66·5)	42 (46·7)	76 (50·7)
Positive		393 (45·6)	129 (43·0)	91 (56·9)	51 (31·7)	48 (53·3)	74 (49·3)
Isoniazid preventive therapy (IPT), n (%)							
On IPT at enrolment		47 (5·5)	10 (3·3)	11 (6·9)	15 (9·3)	8 (8·9)	3 (2·0)
Started IPT after enrolment		370 (43·0)	262 (87·3)	18 (11·2)	24 (14·9)	38 (42·2)	28 (18·7)
Did not take IPT during study		444 (51·6)	28 (9·3)	131 (81·9)	122 (75·8)	44 (48·9)	119 (79·3)
Antiretroviral therapy at enrolment, n (%)							
Naïve		193 (22·4)	57 (19·0)	48 (30·0)	40 (24·8)	12 (13·3)	36 (24·0)
<6 months		115 (13·4)	27 (9·0)	33 (20·6)	32 (19·9)	16 (17·8)	7 (4·7)
6-12 months		66 (7·7)	23 (7·7)	17 (10·6)	12 (7·5)	9 (10·0)	5 (3·3)
>12 months		487 (56·6)	193 (64·3)	62 (38·8)	77 (47·8)	53 (58·9)	102 (68·0)
Started antiretroviral therapy during study, n (%)		142/193 (73·6)	50/57 (87·7)	37/48 (77·1)	27/40 (67·5)	5/12 (41·7)	23/36 (63·9)
Median CD4-positive cells, cells/mm^3 (IQR)		529 (349·5-724·5)	544 (344·5-713·5)	532·5 (361·8-735)	545 (361-748)	515 (335-735)	495·5 (348-697)
Tuberculosis symptoms positive at enrolment, n (%)		51 (5·9)	21 (7·0)	1 (0·6)	3 (1·9)	18 (20·0)	8 (5·3)
RISK11 status, n (%)							
Indeterminate/missing		41 (4·8)	10 (3·3)	4 (2·5)	17 (10·6)	5 (5·6)	5 (3·3)
Positive		285 (33·1)	83 (27·7)	60 (37·5)	36 (22·4)	38 (42·2)	68 (45·3)
Negative		535 (62·1)	207 (69·0)	96 (60·0)	108 (67·1)	47 (52·2)	77 (51·3)
Median RISK11 score (IQR)		32·7 (13·7-76·6)	23·4 (11·2-68·0)	30·2 (13·4-76·6)	27·2 (11·9-59·4)	40·5 (17·2-85·7)	55·4 (23·4-85·7)
**Tuberculosis endpoints**							
Prevalent tuberculosis, n (%; 95% CI)	Primary endpoint (≥2 sample+)	10 (1·2; 0·6-2·1)	3 (1·0; 0·3-2·9)	0	1 (0·6; 0·1-3·4)	0	6 (4·0; 1·8-8·5)
	Secondary endpoint (≥1 sample+)	18 (2·1; 1·3-3·3)	7 (2·3; 1·1-4·7)	0	1 (0·6; 0·1-3·4)	0	10 (6·7; 3·7-11·8)
Incident tuberculosis, n (rate per 100 person-years; 95% CI)	Primary endpoint (≥2 sample+)	9 (1·0; 0·3-1·6)	2 (0·6; 0-1·4)	0	0	2 (2·4; 0-5·6)	5 (3·1; 0·4-5·7)
	Secondary endpoint (≥1 sample+)	21 (2·3; 1·3-3·2)	6 (1·8; 0·4-3·1)	1 (0·5; 0-1·5)	2 (1·2; 0-2·8)	5 (5·7; 0·7-10·3)	7 (4·3; 1·1-7·4)

IQR, inter-quartile range. BMI, body mass index. IGRA, interferon-γ release assay. CI, confidence interval.

Table S3: Baseline characteristics and RISK11 status of study cohort by censoring status

	Participants included in prognostic analysis who completed study follow-up [1] N = 693	Participants included in prognostic analysis who discontinued study follow-up (censored at last visit)[2] N = 119	P-value[3]
**Baseline characteristics at study enrolment**			
Female sex, n (%)	505 (72·9)	85 (71·4)	0·74
Median age, years (IQR)	35 (29-42)	32 (25-40)	**0·005**
Ethnicity, n (%)			
Black African	591 (85·3)	93 (78·2)	
Mixed Ancestry	102 (14·7)	26 (21·8)	0·05
Median BMI, kg/m^2 (IQR)	24·8 (20·8-31·6)	22·4 (19·9-27·6)	**0·0004**
Smoking history, n (%)	264 (38·1)	48 (40·3)	0·64
Prior tuberculosis, n (%)	178 (25·7)	23 (19·3)	0·14
Tuberculosis household contacts, n (%)	134 (19·3)	21 (17·6)	0·66
IGRA result, n (%)			
Not available	3 (0·4)	2 (1·7)	
Negative	382 (55·1)	62 (52·1)	
Positive	308 (44·4)	55 (46·2)	0·25
On Isoniazid preventive therapy at enrolment, n (%)	39 (5·6)	7 (5·9)	0·91
Antiretroviral therapy at enrolment, n (%)			
Naïve	147 (21·2)	32 (26·9)	
<6 months	84 (12·1)	22 (18·5)	
6-12 months	54 (7·8)	11 (9·2)	
>12 months	408 (58·9)	54 (45·4)	**0·043**
Median CD4-positive cells, cells/mm^3 (IQR)	531 (358·5-728·5)	499 (324-696·5)	0·2
Tuberculosis symptoms positive, n (%)	33 (4·8)	12 (10·1)	**0·019**
RISK11 status, n (%)			
Positive	227 (32·8)	51 (42·9)	
Negative	466 (67·2)	68 (57·1)	**0·031**
Median RISK11 score (IQR)	30·3 (13-73·2)	36·5 (16·7-84)	**0·022**

IQR, inter-quartile range. BMI, body mass index. IGRA, interferon-γ release assay. CI, confidence interval. NA, not applicable.

[1]Includes incident tuberculosis cases.

[2]Excludes participants who discontinued follow up due to tuberculosis diagnosed during the conduct of the study.

[3]P-values from Wilcoxon Rank Sum test (continuous data) or Pearson's Chi-squared test (categorical data).

Table S4: Prognostic performance of RISK11 and interferon-γ release assay for incident microbiologically-confirmed tuberculosis through 12 months of follow-up

	Prognostic performance through 12 months (365 days)	
	RISK11(60)[1]	IGRA
**Secondary endpoint (≥1 sample+)**		
Cumulative incidence ratio (95% CI; p value)	8 (1·7-37·5; 0·0082)	1·7 (0·5-6·1; 0·39)
AUC (95% CI)	80·2 (71·3-86·8)	62·1 (41·5-79·1)
Sensitivity, % (95% CI)	80·1 (43·2-95·5)	59·3 (27·8-84·6)
Specificity, % (95% CI)	67·9 (64·3-71·2)	55·6 (51·9-59·3)
PPV[2], % (95% CI)	3·3 (1·7-6·5)	1·8 (0·8-4·0)
NPV[2], % (95% CI)	99·6 (98·4-99·9)	99·0 (97·4-99·6)

IGRA, interferon-γ release assay. CI, confidence interval. AUC, area under the receiver operating characteristic curve. PPV, positive predictive value. NPV, negative predictive value.
[1]A-priori (60%) RISK11 score threshold.
[2]Computed using the incidence rates in the study population as appropriate.

Table S5: Sensitivity analysis of RISK11 performance including participants with negative sputum microbiology who were clinically diagnosed with tuberculosis at local clinics and started on empiric tuberculosis therapy during the conduct of the study as tuberculosis cases

	Secondary endpoint (≥1 sample+)	Exploratory endpoint (≥1 sample+ AND clinically diagnosed cases)
**RISK11 prognostic performance through 15 months[1]**		
Participants included in analysis	799	799
Incident tuberculosis, n (rate per 100 person-years ; 95% CI)	20 (2·3; 1·3-3·3)	25 (2·9; 1·7-3·9)
Cumulative incidence ratio (95% CI; p value)	6·1 (2·2-16·5; 0·0004)	6·4 (2·6-15·8; <0·0001)
AUC (95% CI)	74·8 (64·2-83·1)	76·1 (67·0-83·3)
Sensitivity, % (95% CI)	74·9 (51·3-89·4)	76·1 (55·5-89·1)
Specificity, % (95% CI)	69·0 (65·5-72·4)	69·0 (65·5-72·4)
PPV[2], % (95% CI)	6·5 (4·0-10·6)	8·1 (5·2-12·4)
NPV[2], % (95% CI)	99·0 (97·5-99·6)	98·8 (97·3-99·4)

CI, confidence interval. AUC, area under the receiver operating characteristic curve. PPV, positive predictive value. NPV, negative predictive value.
[1]A-priori (60%) RISK11 score threshold.
[2]Computed using the incidence rates in the study population.

Table S6: Linear regression analysis examining factors associated with RISK11 score in participants without tuberculosis disease at baseline[1]

	Univariable analysis			Multivariable regression model[2]	
	**Degrees of freedom**	**β (95% CI)**	**p value**	**β (95% CI)**	**p value**
Sex					
Female		Reference		Reference	
Male	782	2·8 (-2·4,7·9)	0·29	-7·5 (-13·7,-1·4)	0·017
Age, per 10 years	782	-2·5 (-5·0,0)	0·053	-3·2 (-6·3,-0·1)	0·043
Ethnicity					
Black African		Reference		Reference	
Mixed Ancestry	782	8·3 (2·0,14·7)	0·01	0·9 (-6·3,8·2)	0·8
Weight, per 10kg	782	-2·3 (-3·6,-1·0)	0·0006	-1·4 (-3·1,0·3)	0·11
Prior tuberculosis					
No		Reference			
Yes	782	0·7 (-4·7,6·0)	0·81		
Tuberculosis household contacts					
No		Reference			
Yes	782	-1·2 (-6·9,4·6)	0·69		
Smoking history					
No		Reference			
Yes	782	1·8 (-2·9,6·5)	0·45		
IGRA status					
Negative		Reference		Reference	
Positive	777	-4·9 (-9·4,-0·3)	0·037	-2·5 (-8·0,3·1)	0·38
Isoniazid preventive therapy at enrolment					
Not on therapy		Reference			
On therapy	782	-9·3 (-18·9,0·4)	0·06		
Antiretroviral therapy at enrolment					
Naïve		Reference		Reference	
Experienced	782	-21·4 (-26·7,-16·1)	<0·0001	-1·6 (-8·1,4·8)	0·62
CD4-positive cell count (cells/mm^3), per 50 cells	780	-2·5 (-2·8,-2·1)	<0·0001	-1·5 (-2·0,-1·0)	<0·0001
HIV plasma viral load					
<100 copies/mL		Reference		Reference	
≥100 copies/mL	366	36·0 (30·5,41·5)	<0·0001	27·0 (20·1,34·0)	<0·0001
Tuberculosis symptoms at enrolment					
No		Reference		Reference	
Yes	782	12·1 (2·3,22·0)	0·016	7·1 (-5·7,19·9)	0·28
Season at enrolment					
Winter		Reference			
Spring		1·3 (-5·0,7·7)	0·68		
Summer		-3·4 (-9·5,2·7)	0·27		
Autumn	780	5·7 (-0·7,12·1)	0·082		

CI, confidence interval. BMI, body mass index. IGRA, interferon-γ release assay.

[1]All microbiologically-confirmed tuberculosis (≥1 sample+) cases were excluded from this analysis.

[2]Median residuals 0·04 (IQR -19·1,16·0); Residual standard error = 25·4 on 353 degrees of freedom (421 observations deleted due to missing weight, HIV plasma viral load, CD4 cell count, or IGRA result); R^2 = 0·39; Adjusted-R^2 = 0·38, p <0·0001

Table S7: Sensitivity analysis of RISK11 performance excluding participants who received Isoniazid preventive therapy during the conduct of the study

	All participants with RISK11 results	Excluding participants who received IPT at baseline	Excluding participants who received IPT at baseline or during follow-up
Test+ prevalence, n/N (%)	285/820 (34·8)	276/774 (35·7)	172/421 (40·9)
**RISK11 diagnostic performance at baseline (Primary endpoint; ≥2 sample+)[1]**			
Prevalent tuberculosis, n (%; 95% CI)	8 (1; 0·5-1·9)	8 (1; 0·5-2)	7 (1·7; 0·8-3·4)
Risk ratio (95% CI; p value)	13·1 (2·1-81·6; 0·0016)	12·6 (2·0-78·5; 0·0021)	NA[3]
AUC, % (95% CI)	88·2 (77·6-96·7)	87·9 (77·4-96·6)	91·1 (83·4-98·4)
Sensitivity, % (95% CI)	87·5 (58·3-100)	87·5 (60·0-100)	100 (NA[3])
Specificity, % (95% CI)	65·8 (62·5-69·0)	64·9 (61·5-68·2)	60·1 (55·4-64·8)
PPV[2], % (95% CI)	2·5 (0·7-4·4)	2·5 (0·8-4·5)	4·1 (1·3-7·3)
NPV[2], % (95% CI)	99·8 (99·4-100)	99·8 (99·4-100)	100 (NA[3])
**RISK11 diagnostic performance at baseline (Secondary endpoint; ≥1 sample+)[1]**			
Prevalent tuberculosis, n (%; 95% CI)	16 (2·0; 1·2-3·1)	16 (2·1; 1·3-3·3)	13 (3·1; 1·8-5·2)
Risk ratio (95% CI; p value)	5·6 (1·9-16·4; 0·0006)	5·4 (1·9-15·8; 0·0009)	8 (2·0-31·8; 0·0011)
AUC, % (95% CI)	80·3 (71·4-88·2)	79·8 (70·8-88·1)	83·4 (74·1-91·3)
Sensitivity, % (95% CI)	75·0 (50·0-94·4)	75·0 (50·0-94·4)	84·6 (62·5-100)
Specificity, % (95% CI)	66·0 (62·7-69·2)	65·2 (61·8-68·6)	60·5 (55·7-65·2)
PPV[2], % (95% CI)	4·2 (2·0-6·7)	4·3 (2·1-6·9)	6·4 (3·0-10·2)
NPV[2], % (95% CI)	99·3 (98·5-99·8)	99·2 (98·3-99·8)	99·2 (98·0-100)
**RISK11 prognostic performance through 15 months (Primary endpoint; ≥2 sample+)[1]**			
Participants included in analysis	807	761	409
Incident tuberculosis, n (rate per 100 person-years ; 95% CI)	8 (0·9; 0·3-1·6)	8 (1·0; 0·3-1·7)	5 (1·3; 0·2-2·4)
Cumulative incidence ratio (95% CI; p value)	16·0 (2·0-129·5; 0·0092)	15·3 (1·9-123·3)	NA[3]
AUC (95% CI)	80·0 (70·6-86·9)	79·2 (69·5-86·4)	74.3 (63.5-82.8)
Sensitivity, % (95% CI)	88·6 (43·5-98·7)	88·6 (44·1-98·7)	100 (NA[3])
Specificity, % (95% CI)	68·9 (65·3-72·3)	67·8 (64·1-71·3)	64.1 (58.6-69.2)
PPV[2], % (95% CI)	3·2 (1·5-6·6)	3·3 (1·6-6·7)	4.2 (1.8-9.8)
NPV[2], % (95% CI)	99·8 (98·6-100)	99·8 (98·6-100)	100 (NA[3])
**RISK11 prognostic performance through 15 months (Secondary endpoint; ≥1 sample+)[1]**			
Participants included in analysis	799	753	403
Incident tuberculosis, n (rate per 100 person-years ; 95% CI)	20 (2·3; 1·3-3·3)	20 (2·4; 1·4-3·4)	12 (2·9; 1·3-4·5)
Cumulative incidence ratio (95% CI; p value)	6·1 (2·2-16·5; 0·0004)	5·8 (2·1-15·6; 0·0006)	16·5 (2·2-126·4; 0·007)
AUC (95% CI)	74·8 (64·2-83·1)	74·2 (63·6-82·5)	73 (58·1-84·1)
Sensitivity, % (95% CI)	74·9 (51·3-89·4)	74·9 (51·2-89·4)	91·1 (55·3-98·8)
Specificity, % (95% CI)	69·0 (65·5-72·4)	67·9 (64·1-71·4)	64·6 (59·0-69·8)
PPV[2], % (95% CI)	6·5 (4·0-10·6)	6·7 (4·0-10·9)	8·7 (4·9-15·1)
NPV[2], % (95% CI)	99·0 (97·5-99·6)	98·9 (97·3-99·5)	99·5 (96·5-99·9)

IPT, Isoniazid preventive therapy. CI, confidence interval. AUC, area under the receiver operating characteristic curve. PPV, positive predictive value. NPV, negative predictive value.

[1]A-priori (60%) RISK11 score threshold.

[2]Computed using the prevalence and incidence rates in the study population as appropriate.

[3]There were no prevalent or incident tuberculosis cases amongst RISK11- participants who did not receive IPT.

Figure S1

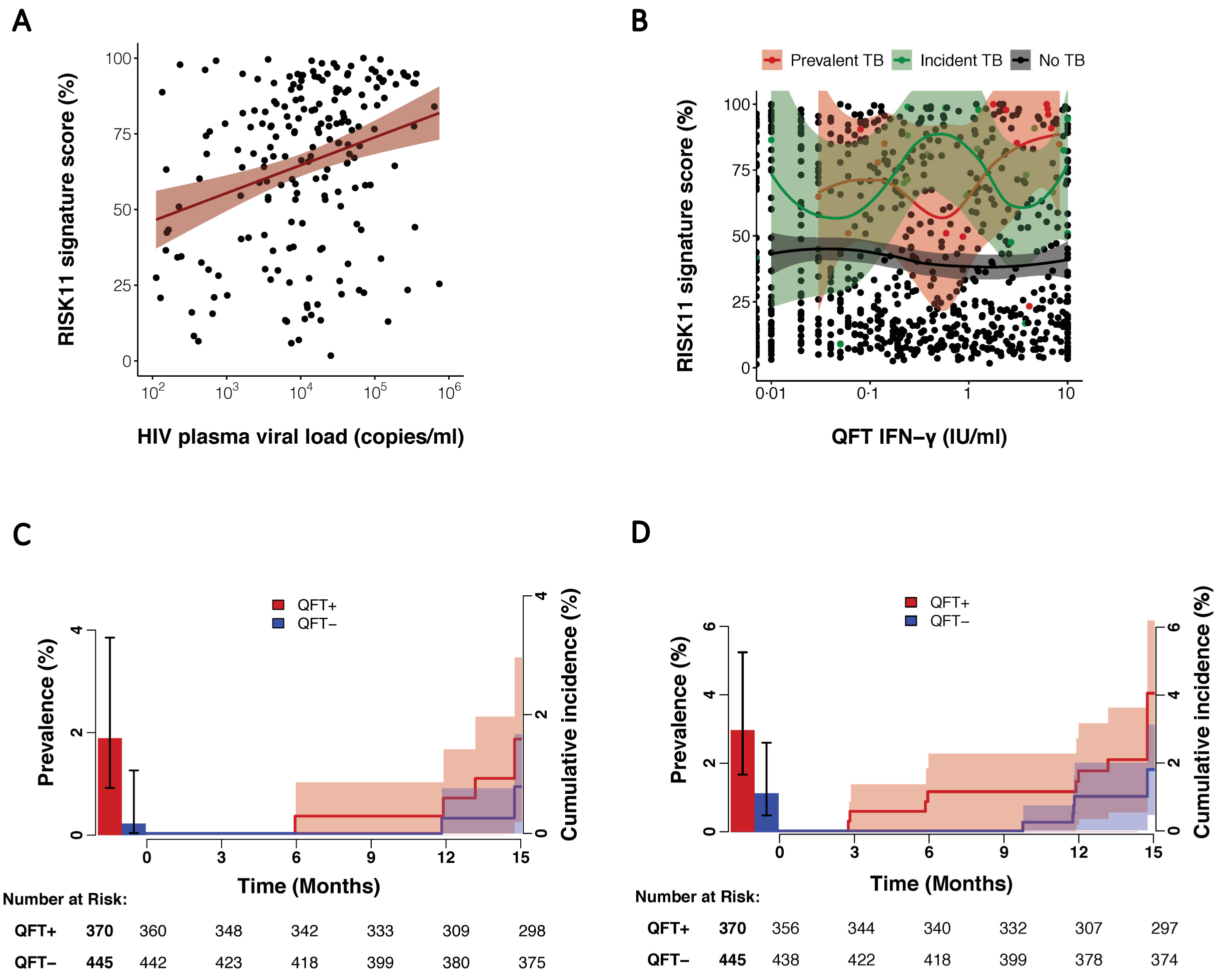

(A) Correlation between HIV plasma viral load and RISK11 signature scores. Correlation between HIV plasma viral load and RISK11 signature scores for participants with no tuberculosis diagnosis (N=198; Spearman ρ 0·29, p < 0·0001). Each dot represents a participant, the locally estimated scatterplot smoothing (LOESS) curve represents the local polynomial regression, and the shaded area represents the 95% confidence interval (CI) on the LOESS regression.

(B) Correlation between QFT interferon-γ (IFN-γ) response and RISK11 signature scores. Correlation between IFN-γ response and RISK11 signature scores for participants with prevalent tuberculosis (Prevalent TB; Spearman ρ 0·41, p = 0·12), participants who progressed to incident tuberculosis (Incident TB; Spearman ρ 0·08, p = 0·74), and participants with no tuberculosis diagnosis (No TB; Spearman ρ -0·07, p = 0·04). Each dot represents a participant, the LOESS curve represents the local polynomial regression, and the shaded area represents the 95% confidence interval (CI) on the LOESS regression.

(C-D) Prevalence and cumulative incidence of (C) primary (≥2 sample+), and (D) secondary (≥1 sample+) tuberculosis endpoint cases in QFT+ and QFT- participants, at study enrolment and through 15 months follow-up. Error bars depict the 95% confidence interval (CI). The shaded areas represent the 95% CI and the numbers below the graph represent the number of participants at risk at the indicated study time.

PROTOCOL TITLE

VALIDATION OF CORRELATES OF RISK OF TB DISEASE IN HIGH RISK POPULATIONS (CORTIS-HR)

A Companion Study of the CORTIS-01 Trial

PROTOCOL NUMBER:	CORTIS-HR
PROTOCOL DATE:	26th August 2016
PROTOCOL VERSION:	Version 1.0
SPONSORED BY:	University of Cape Town

Information contained in this protocol is confidential in nature, and may not be used, divulged, published, or otherwise disclosed to others except to the extent necessary to obtain approval of the Institutional Review Board or Research Ethics Board, or as required by law. Persons to whom this information is disclosed should be informed that this information is confidential and may not be further disclosed without the express permission of the Sponsor.

SITE PRINCIPAL INVESTIGATOR SIGNATURES OF AGREEMENT FOR PROTOCOL IMPLEMENTATION

SATVI Site PI	Date
Aurum Klerksdorp Site PI	Date
Aurum Rustenburg Site PI	Date
CAPRISA Site PI	Date
Stellenbosch University Site PI	Date

INVESTIGATOR APPROVAL STATEMENT

I have read the protocol and agree that it contains all necessary details for carrying out the study as described. I will conduct this protocol as outlined therein and will make a reasonable effort to complete the study within the time designated.

I agree to personally supervise the study.

I will ensure that the requirements related to obtaining informed consent are in accordance with ICH Guidelines for Good Clinical Practice (GCP) section 4.8 and local requirements.

I agree to promptly report to the Ethics Committee (EC) all changes in the research activity and all unanticipated problems involving risk to the participants. I will not make any changes to the conduct of the study without the EC and Sponsor approval, except when necessary to eliminate apparent immediate harm to participants.

I agree to maintain adequate and accurate records and make those records available in accordance with ICH guidelines for Good Clinical Practices (GCP) section 4.11 and local requirements.

I agree to ensure that all associates, colleagues and employees assisting in the conduct of the study are informed about their obligations in meeting the above commitments.

I understand that the study may be terminated or enrolment suspended at any time by the Sponsor, with or without cause, or by me if it becomes necessary to protect the best interest of the participants.

________________________________ ____________________

National Principal Investigator Signature Date

RESPONSIBILITIES

**Sponsor:**	University of Cape Town
**National Principal Investigator:**	Mark Hatherill
**Regulatory Functions:**	Triclinium Clinical Development
**Site Monitoring Functions:**	Triclinium Clinical Development
**Data Management Functions**	Triclinium Clinical Development
**Statistical Analysis:**	The Statistical Center for HIV/AIDS Research & Prevention (SCHARP)
**Clinical Laboratory:**	BARC, Johannesburg
**Analytical Laboratory:**	South African Tuberculosis Vaccine Initiative (SATVI)

PROTOCOL SYNOPSIS

TITLE	**Validation of Correlates of Risk of TB Disease in High Risk Populations (CORTIS-HR)**
BACKGROUND	Effective tuberculosis (TB) control requires that people who progress from latent *Mycobacterium tuberculosis* (MTB) infection (LTBI) to TB disease are identified and treated before they become symptomatic and infect others. A prognostic correlate of risk (COR), based on mRNA expression signatures, which prospectively discriminates between TB cases and healthy controls, has been constructed and validated in HIV uninfected persons.<br>Based on published microarray case-control datasets, this COR has 87% diagnostic sensitivity and 97% specificity for prevalent TB disease in HIV uninfected South African adults; and in two nested case-control studies, also among HIV uninfected persons, the COR has 70% prognostic sensitivity and 84% specificity for incident TB disease occurring within one year of sampling. Based on analysis of published microarray data, COR diagnostic performance for discriminating prevalent TB disease from latent TB infection in HIV infected persons appears to be reduced by approximately 10%, compared to HIV uninfected persons. ***Diagnostic and prognostic performance of the COR has not yet been tested in a prospective cohort of HIV infected persons***.<br><br>The diagnostic and prognostic performance of the blood RNA signature of risk for TB will be validated in HIV infected persons, in a companion study of the CORTIS-01 trial. HIV infected persons bear a disproportionate TB disease burden, despite antiretroviral therapy (ART) and isoniazid preventive therapy (IPT). Modeling indicates that prevention of TB disease in HIV infected persons, using a COR 'screen and treat' strategy to target preventive therapy for those at highest risk, would have major impact on the TB epidemic in Sub-Saharan Africa, with considerable indirect benefit to the HIV uninfected population.
AIMS	**Primary Aim:**<br>1. Test whether COR status differentiates HIV infected persons with cumulative prevalent or incident TB disease from those without TB disease.<br><br>**Secondary Aims:**<br>**1.** Estimate whether COR status differentiates HIV infected persons with prevalent TB disease from those without prevalent TB disease<br>2. Estimate whether COR status differentiates HIV infected persons at high risk for incident TB disease from those at low risk for incident TB disease<br>**3.** Compare prognostic performance of the COR for incident TB disease with Interferon-gamma release assay (IGRA) in HIV infected persons.<br><br>**Exploratory Aims:**<br>**1.** Assess and model the impact of a COR screen & treat strategy on reducing the rate of incident TB disease and TB mortality among HIV infected persons in South Africa.<br>2. Re-parameterize the COR assay for prevalent and incident TB disease in HIV infected persons.<br>3. Test the performance of additional validated COR signatures in distinguishing HIV infected persons with cumulative prevalent or incident TB disease from those without TB disease.<br>4. Compare prognostic performance of the COR for incident TB disease in HIV infected persons before and 3 months after starting IPT and/or ART.
STUDY SIZE	860 HIV infected adults

STUDY POPULATION	Adult volunteers living in TB hyperendemic communities of South Africa will be consented and screened. Persons aged 18 to less than 60 years, including those who are ineligible for the CORTIS-01 trial on the basis of HIV infection, will be approached to enroll in CORTIS-HR.<br><br>*Inclusion criteria (at time of screening):*<br>1. Written informed consent<br>2. Aged ≥18 and <60 years<br>3. HIV infection<br>4. Likely to remain in follow-up and adhere to protocol requirements<br><br>*Exclusion criteria (at time of screening):*<br>1. Pregnant or lactating<br>2. Diagnosed with TB disease within last 3 years<br>3. Household exposure to a TB patient with known multi-drug resistant (MDR-) TB disease within last 3 years<br>4. Any medical, surgical, or other condition, including but not limited to known diabetes mellitus (requiring oral or injectable therapy), liver disease, or alcoholism, that in the opinion of the Investigator is likely to interfere with COR performance; safety or efficacy of ART and/or IPT; or adherence to protocol requirements.
STUDY DESIGN	CORTIS-HR is an observational study of COR diagnostic and prognostic performance in HIV infected persons. Following sample collection for the COR assay at Visit 1 (Day 0), all participants will be screened for prevalent TB. All eligible participants without prevalent TB will be referred for at least 12 months (IGRA negative) or 36 months (IGRA indeterminate or positive) IPT. All HIV infected persons eligible for ART, as per SA national guidelines, will be referred in writing to the clinic for ART. Thereafter, all participants will be followed for 15 months for incident TB disease (3 telephone contacts and 4 site visits). Symptoms consistent with TB disease will be solicited at study visits and presence of one or more symptoms will trigger TB investigation. The performance of the COR will be evaluated by comparing the cumulative incidence of endpoint-defined TB disease over 15 months in COR+ versus COR- participants (RR_{COR}).<br><br>We will perform an interim analysis of COR prevalence and diagnostic performance for prevalent TB cases. The results will inform a Stop/Go decision for continuation of study follow-up beyond 3 months in all subjects to evaluate COR prognostic performance. In subjects who continue follow-up for incident TB disease, we will perform a sensitivity analysis of month 3 COR prognostic performance to evaluate the impact of IPT and/or ART on COR score and TB risk.
INVESTIGATIONS	Blood will be sampled at Visit 1 (Day 0) for QuantiFERON-Plus (QFT) and CD4 cell count; blood will be collected for HIV viral load (VL) only in those subjects not yet established on ART; whole blood RNA will be collected in PAXgene tubes for the COR assay; and urine will be collected for lipoarabinomannan (LAM) assay. Aliquots of plasma, serum and urine will be stored for later molecular, proteomic and metabolomic studies.<br><br>COR assay and serum and urine collection will be repeated in all subjects at month 3 to evaluate COR dynamics and the impact of IPT on the COR score. CD4 count and VL will be repeated at 3 months in those subjects starting ART. Risk of TB disease will be evaluated using the qualified BioMark HDFluidigm multiplex qRT-PCR COR assay at the SATVI Laboratory.<br><br>All participants will undergo TB symptom screening and will provide two sputum samples for paired Xpert MTB/RIF and MGIT culture at Visit 1 (Day 0). Thereafter, symptoms consistent with TB disease will be solicited at all study visits; and presence of one or more symptoms will trigger TB investigation (paired sputum Xpert MTB/RIF and MGIT culture). All participants will undergo TB investigation again at end of study and submit two sputum samples for paired sputum Xpert MTB/RIF and MGIT culture. Throughout follow-up,

	additional tests for clinical suspicion of TB disease, including, but not limited to chest radiography and sputum induction, may be performed if deemed indicated by the investigator. Investigator decisions to start TB treatment on clinical grounds will be made independently of study-specific endpoint determination.
STUDY DURATION	All participants will be followed for 15 months for incident TB disease (3 telephone contacts and 4 site visits).
SITES	5 or more study sites in South Africa.
STUDY ENDPOINTS	The primary endpoint will be defined as Xpert MTB/RIF and/or MGIT culture positive TB disease, confirmed on two separate sputum samples; or on samples from any other site in the case of extrapulmonary TB disease.
STATISTICAL CONSIDERATIONS	The primary analysis will evaluate $RR_{COR}(15)$, relative-risk for TB disease over 15 months of follow-up. The primary outcome measure is Relative Risk (RR, 95% CI) for TB disease, as per the TB case endpoint definition. Based on our underlying assumptions for COR prevalence, ART and IPT usage, and TB prevalence and incidence, it is expected that of approximately 215 (25%) newly diagnosed HIV infected participants yet to start ART, we will identify 31 (95% CI 22, 42) prevalent TB cases at screening; and an additional 5 (95% CI 2, 10) participants who will develop active TB disease over 15 months of follow-up. It is expected that of the approximately 645 (75%) HIV infected participants already on chronic ART, we will identify 12 (95% CI 6, 20) prevalent TB cases at screening; and an additional 9 (95% CI 4, 15) incident TB cases over 15 months of follow-up. Thus, it is expected that we will measure the COR in a total of 58 (95% CI 44, 73) cumulative endpoint TB cases (43 prevalent and 14 incident TB cases) and 802 (95% CI 787, 816) controls.<br><br>Based on these assumptions, it is estimated that the RR of a COR+ HIV infected participant for cumulative TB disease, relative to a COR- HIV infected participant, will be approximately 19 (95% CI 11-34). Corresponding estimates of precision for RR for prevalent cases only are 95% CI 10-37; and for incident cases only are 95% CI 6-65.
ETHICAL CONSIDERATIONS	*Risks:* HIV infected persons are at several-fold higher risk of TB disease compared to HIV uninfected persons, even if established on chronic ART, which reduces incident TB disease by between 45-65%. SA national guidelines now recommend initiation of ART for all HIV infected persons with CD4 count ≤500 cells/mm^3. IPT offers added protection, estimated to be in the range 30 – 50%, with isolated studies reporting up to 83% risk reduction. It is estimated that approximately 75% of CORTIS-HR participants will be receiving chronic ART at Visit 1 (Day 0), 10-20% of whom will be receiving chronic IPT.<br><br>*Measures to Ensure Safety*: WHO guidelines currently recommend that all HIV infected persons in high TB transmission regions with unknown or positive tuberculin skin tests (TST) receive ART and at least 36 months of IPT. South African national guidelines additionally recommend that TST negative HIV infected persons receiving ART should also receive at least 12 months of IPT. Therefore, all HIV infected persons not receiving, but eligible for ART, will be referred in writing to the state health services to access the appropriate care. Additionally, all eligible participants will be referred for at least 12 months (QFT negative) or 36 months (QFT indeterminate or positive) of IPT. All participants diagnosed with prevalent TB disease at Visit 1 (Day 0) will discontinue study follow-up and will be referred for curative treatment. Thereafter, active symptom-based surveillance and investigation for incident TB disease will allow early diagnosis and effective treatment; all participants will be investigated for TB at end of study. Symptomatic participants who test sputum Xpert MTB/RIF negative on two samples, or who are sputum unproductive, may undergo additional investigations if deemed necessary by the investigator. Symptomatic, Xpert MTB/RIF negative participants may, at the discretion of the investigator, undergo a course of broad-spectrum antibiotics as trial of therapy prior to further investigation. Participants with a clinical or radiological suspicion of TB disease not meeting the study endpoint definition will be referred for curative treatment if indicated in the judgment of the investigator.

LIST OF ABBREVIATIONS

Abbreviation	Text
AFB	Acid-fast bacilli
BMI	Body Mass Index (BMI)
cDNA	Copy DNA
CI	Confidence interval
COR	Correlate of Risk
CRA	Clinical research associate
CRF	Case report form
CRO	Clinical Research Organization
DAIDS	NIH Division of AIDS
eCRF	electronic CRFs
FDA	Food and Drug Administration
GCP	Good Clinical Practice
H_0	Null hypothesis
HIV	Human Immunodeficiency Virus
IEC	Independent ethics committee
IGRA	Interferon gamma release assay
INH	Isoniazid
IPT	INH preventive therapy
LTBI	Latent tuberculosis infection
MDR-TB	multi-drug resistant tuberculosis
MGIT	Mycobacteria Growth Indicator Tube
mRNA	Messenger RNA
MTA	Material Transfer Agreement
MTB	*Mycobacterium tuberculosis*
NHP	Non-human primate
NTP	National TB Programme
PI	Principal Investigator
QFT	QuantiFERON
RR	Relative risk
$RR_{COR}(15)$	Relative risk for TB disease over 15 months
SA	South Africa
SATVI	South African Tuberculosis Vaccine Initiative
TB	Tuberculosis
TCD	Triclinium Clinical Development
TST	Tuberculin Skin Test
WHO	World Health Organization

SCHEDULE OF EVENTS

All participants (n=860)

Description	Screening & Enrolment	Follow-up						End of Study
**Trial Visit**	**Visit 1**	**Contact 2**	**Contact 3**	**Visit 4**	**Visit 5**	**Contact 6**	**Visit 7**	**Visit 8**
**Day**	**D0**	**M1**	**M2**	**M3**	**M6**	**M9**	**M12**	**M15**
Informed consent[1]	x							
Age verification	x							
Medical history	x							
Height	x							
Weight	x			x	x		x	x
Urine pregnancy test (females)	x							
HIV counselling & testing	x							
Vital signs	x			x	x		x	x
Targeted physical examination	x			x	x		x	x
Verification of eligibility	x							
Phlebotomy[2]	x			x				
COR (PAXgene RNA)	x			x				
CD4 count (cells/mm^3)	x			x[7]				
HIV Viral load (copies/mL)	x[6]			x[7]				
IGRA (IU/mL)	x							
Serum (proteomics)	x			x				
Plasma (molecular assays)	x							
Urine (LAM and metabolomics)	x			x				
TB symptom screen	x	x	x	x	x	x	x	x
TB Investigations	xx[3]	xx[4]	xx[4]	xx[4]	xx[4]	xx[4]	xx[4]	xx[5]
Concomitant Medications	x	x	x	x	x	x	x	x

[1] May be conducted at prior field visit
[2] Phlebotomy for further investigations only if eligible
[3] One sputum sample for Xpert MTB/RIF; one sputum sample for MGIT culture; store aliquot of unprocessed sputum from each (all participants)
[4] If indicated by positive TB symptom screen, one sputum sample for Xpert MTB/RIF; one sputum sample for MGIT culture
[5] One sputum sample for Xpert MTB/RIF; one sputum sample for MGIT culture (all participants)
[6] HIV viral load only in subjects not yet established on ART
[7] CD4 count and viral load repeated at 3 months only in participants starting ART

BACKGROUND

Two billion people worldwide, including the majority of adults in TB endemic countries, are *Mycobacterium tuberculosis* (MTB) infected. These latently infected individuals, identified by a positive tuberculin skin test (TST) or interferon-gamma release assay (IGRA), have higher risk of developing TB disease than uninfected people. Unfortunately, TST and IGRA have poor specificity for incident TB disease in endemic populations, including HIV infected people.

We have previously developed a highly specific prognostic correlate of risk (COR) to identify healthy, HIV uninfected, South African adults at high risk of active TB disease[1]. This validated COR, based on mRNA expression signatures in blood, prospectively discriminates between TB cases and healthy controls among HIV uninfected persons [1]. Based on published microarray case-control datasets, the COR has 87% diagnostic sensitivity and 97% specificity for prevalent TB disease in HIV uninfected South African adults [2-4]; and in two nested case-control studies, also among HIV uninfected persons, the COR has 70% prognostic sensitivity and 84% specificity for incident TB disease occurring within one year of sampling [1-4].

Based on analysis of published microarray data, COR diagnostic performance for discriminating prevalent TB disease from latent TB infection in HIV infected persons appears to be reduced by approximately 10%, compared to HIV uninfected persons [5]. Our preliminary data support this estimate. In a small pilot study of 100 participants, diagnostic performance (area under the Receiver Operating Characteristic curve) of the qRT-PCR-based COR decreased by 13% when applied to HIV infected participants, compared to HIV uninfected participants. Reduced COR sensitivity and specificity in HIV infected persons might be related to chronic stimulation of anti-viral type-I interferon responses and/or immune dysfunction. However, performance of the COR, which was discovered in HIV uninfected persons, might be further improved by re-parameterizing the gene expression signature to account for HIV status. Smaller, parsimonious COR models suitable for point-of-care testing are also being evaluated in ongoing studies.

The Dual Epidemics of TB and HIV

TB disease constitutes a major morbidity and mortality burden for HIV infected patients, and a massive logistic and economic burden for healthcare systems in resource-constrained countries like South Africa [6]. Prevalence of HIV infection in adult South Africans is approximately 17% (9.2 million people). HIV infected persons remain at several-fold higher risk of TB disease compared to HIV uninfected persons, even if established on chronic ART, which reduces rate of incident TB disease by between 45-65%[7-9]. Approximately 79% of HIV infected TB patients in South Africa receive chronic ART. However, the World Health Organization (WHO) estimates there were 450,000 TB cases in South Africa in 2014, of which 270,000 were HIV co-infected; with 72,000 deaths due to TB occurring in HIV infected persons (*WHO Global Tuberculosis Report 2015*) [6]. In that year alone, 1.1 million HIV infected South Africans (12%) were screened for TB disease and 500,000 patients were provided with IPT. National TB/HIV services are increasingly integrated and might provide a platform for targeted TB triage, treatment, and prevention, on a wider scale.

Effect of ART on Risk of TB Disease

ART alone is an effective TB preventive therapy for HIV infected persons [7-9]. A recent meta-analysis reported 65% average risk reduction for incident TB disease, with no difference in effect by CD4 cell count strata [8]. Overall, risk for incident TB disease in HIV infected persons on chronic ART is not affected by baseline CD4 cell count [8]. However, some data are conflicting [7,9,10]. The exceptions to the rule are at the upper and lower range of CD4 cell count; and during the period soon after the start of ART [7,9,10]. For example, in a study of 74,000 HIV infected South Africans, ART decreased the rate of incident TB by 45% among those patients with CD4 cell counts less than 350 cells/mm^3, but was not associated with statistically significant protection among those with CD4 cell counts above 350 cells/mm^3 [9]. By contrast, in a study of 65,000 HIV infected persons from high income countries, ART reduced the rate of incident TB by 44% overall, but was not associated with statistically significant protection among those with CD4 cell counts below 50 cells/mm^3 [7]. In a third

study, TB disease incidence among HIV infected South Africans with CD4 cell counts less than 200 cells/mm^3 were 1.7-fold higher in the first 4 months of ART than during chronic ART [10].

Effect of IPT on Risk of TB Disease

Provision of chronic ART is thought to exert greater impact on risk for incident TB disease than IPT alone, but IPT does offer added protection, estimated to be in the range 30 – 50%, with isolated studies reporting up to 83% risk reduction [11-14]. In a study among HIV infected South Africans, addition of IPT to an ART regimen was associated with average 37% risk reduction among both TST positive and TST negative persons, with the greatest benefit within the first year of starting therapy [11]. Adjustment for CD4 cell count did not significantly change the risk for incident TB disease [11]. A recent Cochrane review reported a similar 32% risk reduction overall, with the greatest effect seen among TST positive persons [12]. By contrast, the Brazilian THRio trial, conducted among 2,000 HIV infected TST positive persons, reported 83% reduction in risk for TB disease, with the greatest effect seen among those with CD4 cell counts below 200 cells/mm^3 [14]. It is possible that the effect of IPT may be more modest in high, compared to low, TB burden countries.

Durability of Protection Offered by IPT

We might expect that IPT would not offer durable protection after conclusion of therapy, due in part to reactivation of dormant *Mycobacterium tuberculosis* (MTB) bacilli; and in part to exogenous reinfection. Mathematical modeling suggests that provision of IPT to HIV infected persons receiving ART results in sterilizing cure of LTBI in only 35% of patients [15]. In a trial in Botswana, receipt of chronic IPT for 36 months was shown to reduce the annual incidence of TB disease in HIV infected persons to 0.7%, compared to 1.3% with 6 months of IPT [16], and with waning, but persistent protection after conclusion of IPT only in TST positive participants [17]. It is clear that HIV infected persons need additional protection against 'breakthrough' reactivation TB disease occurring during IPT, as well as TB disease occurring after IPT has ended.

Current Guidelines

SA national guidelines now recommend initiation of ART for all HIV infected persons with CD4 count ≤500 cells/mm^3. WHO guidelines currently recommend that all HIV infected persons in high TB transmission regions with unknown or positive tuberculin skin tests (TST) receive ART and at least 36 months of IPT. South African national guidelines additionally recommend that TST negative HIV infected persons receiving ART should also receive at least 12 months of IPT. It is estimated that at the study sites, approximately 75% of eligible HIV infected persons are receiving ART; and that 10-20% of those receiving ART are currently receiving chronic IPT.

Summary of Factors Affecting TB Risk

Risk of prevalent TB among HIV infected South Africans receiving chronic ART, across all CD4 cell count strata, is estimated at 2%, with historically higher rates in the range 4-6% [18,19]. Twelve-month risk of incident TB is also estimated at 2%, with historically higher rates in the range 4-10% [20]. Corresponding risk of prevalent TB in HIV infected persons not receiving, or newly established on ART, is estimated at 15% [21,22]; and 12-month risk of incident TB is estimated at 4% [21,22]. Addition of IPT to a chronic ART regimen might further reduce these estimates by 30 – 50%. Although some data are conflicting, it is unlikely that baseline CD4 cell count will have major impact on TB incidence in persons receiving chronic ART and IPT [7,9,10], but it is possible that any added benefit of IPT is maximal in TST positive persons [12,14].

RATIONALE

A conservative model of the South African epidemic indicates that a COR targeted 'screen & treat' strategy, independent of parallel improvements in TB therapeutics, could reduce overall TB incidence by 27% and TB mortality by 35% within 5 years. Key to population-level impact of the strategy is that COR targeted TB preventive therapy is effective for both HIV uninfected and HIV infected persons, in any community in which the strategy is rolled out. Additional modelling suggests that if the strategy were targeted only at HIV infected adults, this population would benefit directly by 15% reduction in TB incidence; and the HIV uninfected population would benefit indirectly by 16%

reduction over the same period. Thus, prospective validation of COR performance in HIV infected persons, and thereafter, demonstration of efficacy of TB preventive therapy in HIV infected COR+ persons, is crucial to success of the 'screen and treat' strategy in the countries of Sub-Saharan Africa affected by the dual epidemics of TB and HIV.

RISKS

Risk of TB Disease: HIV infected persons are at several-fold higher risk of TB disease compared to HIV uninfected persons, even if established on chronic ART, which reduces incident TB disease by between 45-65%. SA national guidelines now recommend initiation of ART for all HIV infected persons with CD4 count ≤500 cells/mm^3. IPT offers added protection, estimated to be in the range 30 – 50%, with isolated studies reporting up to 83% risk reduction. We estimate that approximately 75% of CORTIS-HR participants will be receiving chronic ART at baseline, 10-20% of whom will be receiving chronic IPT.

Measures to Minimize Risk of TB Disease: WHO guidelines currently recommend that all HIV infected persons in high TB transmission regions with unknown or positive tuberculin skin tests (TST) receive ART and at least 36 months of IPT. South African national guidelines additionally recommend that TST negative HIV infected persons receiving ART should also receive at least 12 months of IPT. Therefore, all HIV infected persons not receiving, but eligible for ART, will be referred in writing to the state health services to access the appropriate care. Additionally, all eligible participants will be referred for 12 months (QFT negative) or 36 months (QFT indeterminate or positive) of IPT.

All participants diagnosed with prevalent TB disease at Visit 1 (Day 0) will discontinue study follow-up and will be referred for curative treatment. Thereafter, active symptom-based surveillance and investigation for incident TB disease will allow early diagnosis and effective treatment. All participants will also be investigated for TB at end of study. Symptomatic participants who test sputum Xpert MTB/RIF negative on two samples, or who are sputum unproductive, may undergo additional investigations if deemed necessary by the investigator for diagnosis of suspected pulmonary or extra-pulmonary TB. Symptomatic, Xpert MTB/RIF negative participants may, at the discretion of the investigator, undergo a course of broad-spectrum antibiotics as trial of therapy prior to further investigation. Participants with a clinical or radiological suspicion of TB disease not meeting the primary endpoint definition will be referred for curative treatment if indicated in the judgment of the investigator.

Other risks: Other potential risks to participants include risk of breach of confidentiality and disclosure of HIV and/or TB status, which may be associated with social stigma; minor discomfort and bruising associated with phlebotomy; and the time and inconvenience of attending study visits.

Measures to Minimize Other Risks: Identifiable personal information and source documentation will be locked in secure cabinets accessible only to study staff to maintain confidentiality. All study procedures will be performed according to ICH-GCP. Study data will be coded using a personal identifier and stored in a password secured database. All the study sites are experienced research sites with study staff trained in phlebotomy. Discomfort and bruising associated with blood sampling is deemed a minor risk, as is the small volume of blood (<50mL) to be sampled at Visit 1 (Day 0) and 3 months.

BENEFITS

Benefits of Active Case-finding and Active Surveillance for TB Disease: All participants will benefit from active case finding for undiagnosed prevalent TB disease at screening, by symptom screening and collection of sputum for investigation. Similarly, all participants will benefit from TB education and active surveillance for incident TB disease; by active symptom screening and symptom-triggered TB investigation during follow-up; and by repeat sputum screening for undiagnosed TB disease in all participants at end of study. Earlier diagnosis of previously undiagnosed and pre-

symptomatic or incipient TB disease will allow earlier, effective treatment, reduced morbidity, and reduced MTB transmission to susceptible contacts.

Benefits of HIV Diagnosis and Referral for Care: Participants who are newly diagnosed with HIV infection during screening will benefit from post-test counseling and referral for early ART and IPT, which would be expected to reduce AIDS-related morbidity and mortality, including that due to TB co-infection. Participants with known HIV infection who are receiving chronic ART, but not receiving IPT, will benefit from referral and improved linkage to care for TB preventive therapy.

Benefits of Participation in Research: Persons with other previously undiagnosed medical, surgical, or other conditions identified at screening, will benefit from early diagnosis, referral and rapid access to treatment systems. Similarly, participants who develop new conditions during follow-up will also benefit from early diagnosis, referral and linkage to care.

PRIMARY AIM

1. Test whether COR status differentiates HIV infected persons with cumulative prevalent or incident TB disease from those without TB disease.

SECONDARY AIMS

1. Estimate whether COR status differentiates HIV infected persons with prevalent TB disease from those without prevalent TB disease
2. Estimate whether COR status differentiates HIV infected persons at high risk for incident TB disease from those at low risk for incident TB disease
3. Compare prognostic performance of the COR for incident TB disease with Interferon-gamma release assay (IGRA) in HIV infected persons.

EXPLORATORY AIMS

1. Assess and model the impact of a COR screen & treat strategy on reducing the rate of incident TB disease and TB mortality among HIV infected persons in South Africa.
2. Re-parameterize the COR assay for prevalent and incident TB disease in HIV infected persons.
3. Test the performance of additional validated COR signatures in distinguishing HIV infected persons with cumulative prevalent or incident TB disease from those without TB disease.
4. Compare prognostic performance of the COR for incident TB disease in HIV infected persons before and 3 months after starting IPT and/or ART.

STUDY DESIGN

CORTIS-HR is an observational study of COR diagnostic and prognostic performance in HIV infected persons. Following sample collection for the COR assay at Visit 1 (Day 0), all participants will be screened for prevalent TB. All eligible participants without prevalent TB will be referred for at least 12 months (IGRA negative) or 36 months (IGRA indeterminate or positive) IPT. All HIV infected persons eligible for ART, as per SA national guidelines, will be referred in writing to the clinic for ART. Thereafter, all participants will be followed for 15 months for incident TB disease (3 telephone contacts and 4 site visits). Symptoms consistent with TB disease will be solicited at study visits and presence of one or more symptoms will trigger TB investigation. The performance of the COR will be evaluated by comparing the cumulative incidence of endpoint-defined TB disease over 15 months in COR+ versus COR- participants (RR_{COR}).

We will perform an interim analysis of COR prevalence and diagnostic performance for prevalent TB cases. The results will inform a Stop/Go decision for continuation of study follow-up beyond 3 months in all subjects to evaluate COR prognostic performance. In subjects who continue follow-up for incident TB disease, we will perform a sensitivity analysis of month 3 COR prognostic performance to evaluate the impact of IPT and/or ART on COR score and TB risk.

STUDY POPULATION
The study population will include 860 HIV infected adults residing in TB hyperendemic communities at five or more study sites in South Africa.

RECRUITMENT
Primarily, persons screened for the CORTIS-01 trial who are ineligible on the grounds of HIV infection will be approached by study staff to participate in CORTIS-HR. In addition, persons with documented HIV infection may be approached through local ART clinics and Voluntary Counseling & Testing (VCT) services affiliated with or run directly by the study sites. Although recruitment efforts will be focused on persons with documented HIV infection status, in order to maintain confidentiality and avoid risk of stigmatization, community volunteers with unknown HIV status would also be eligible for screening. Any persons screened for CORTIS-HR who are found ineligible due to being HIV uninfected may be approached to participate in the CORTIS-01 trial.

ELIGIBILITY CRITERIA
A participant will be eligible for enrolment in the trial if all inclusion criteria are met. A participant will not be eligible for trial enrolment if any of the exclusion criteria are met.

Inclusion criteria (at time of screening):

5. Written informed consent
6. Aged ≥18 and <60 years
7. HIV infection*
8. Likely to remain in follow-up and adhere to protocol requirements

Exclusion criteria (at time of screening):

5. Pregnant or lactating
6. Diagnosed with TB disease within last 3 years
7. Household exposure to a TB patient with known multi-drug resistant (MDR-) TB disease within last 3 years
8. Any medical, surgical, or other condition, including but not limited to known diabetes mellitus (requiring oral or injectable therapy), liver disease, or alcoholism, that in the opinion of the Investigator is likely to interfere with COR performance; safety or efficacy of ART and/or IPT; or adherence to protocol requirements.

**A documented positive HIV test result obtained for the purpose of CORTIS-01 trial screening will not need to be repeated if the test was performed 28 days or less prior to CORTIS-HR screening.*

CONCOMITANT MEDICATIONS
ART and IPT data will be recorded in all participants throughout the study. Details to be recorded, if known, include the specific medication trade name, the dose and unit, frequency and route of administration, as well as the start and stop dates of the therapy, and prescribing clinic..

PARTICIPANT IDENTIFIER
All participants who are screened for eligibility to participate in CORTIS-HR will be allocated a unique participant identifier. The number will consist of a study-specific prefix, a 1-digit site identifier followed by a 4-digit participant identifier which will be allocated sequentially in accordance with the order in which participants present for screening i.e. the first, second and third participants presenting for screening at Site 1 will be 10001, 10002 and 10003 etc. This number will be used as the participant's primary identifier throughout the study and will be used for all labelling purposes.

VISIT SCHEDULE
After successful screening and enrolment, each participant will undergo a total of seven (7) study contacts or visits, including three (3) study contacts (telephonic or field visits) and four (4) study clinic visits, through 15 months of follow-up.

VISIT 1 (SCREENING & ENROLMENT): DAY 0

Potential participants must provide written informed consent for participation in the study prior to and within 28 days of performing any screening assessments or procedures. If necessary, the participant may take the study information document away with them and return at a later stage for screening examinations. All screening assessments must be completed prior to enrolment.

The following information will be obtained and procedures and assessments performed for the purpose of screening to determine eligibility:

- Written informed consent for study participation
- Verification of age
- Medical history
- Review of concomitant medications (ART and IPT)
- Measurement of height and weight
- Urine pregnancy test (women of child-bearing potential only)
- Blood will be collected for HIV rapid test (with pre- and post-test counselling)*
- Vital signs
- Targeted physical examination
- Verification of eligibility

**Not required if documented positive HIV test obtained during CORTIS-01 screening and within 28 days of CORTIS-HR screening assessment. Any participants newly diagnosed with HIV infection at CORTIS-HR screening may complete post-test counselling and return to complete enrolment within 3 calendar days of screening assessment.*

The following information will be obtained and procedures and assessments performed once eligibility has been determined:

- Blood will be collected for:
    - COR (PAXgene)
    - CD4 count
    - HIV viral load (only in participants not yet established on ART)
    - IGRA (QFT)
    - Serum for proteomics
    - Plasma for viral molecular assays
- Urine will be collected for LAM assay and metabolomics
- TB symptom screen
- Two sputum samples will be collected, one for Xpert MTB/RIF and one for MGIT culture; two aliquots of unprocessed sputum will be stored for additional MTB diagnostic tests at the end of study (potentially including, but not limited to Xpert MTB/RIF, MGIT culture, and line probe assay).

Review eligibility to start ART and/or IPT and refer to health services if indicated

CONTACT 2: DAY 28 (+/- 3)

All participants will undergo the following assessments and procedures:

- Review of concomitant medications (ART and IPT)
- TB symptom screen
- If indicated by one or more TB symptoms, two sputum samples will be collected, one for Xpert MTB/RIF and one for MGIT culture (field or study clinic visit)

CONTACT 3: DAY 56 (+/- 3)

All participants will undergo the following assessments and procedures:

- Review of concomitant medications (ART and IPT)
- TB symptom screen
- If indicated by one or more TB symptoms, two sputum samples will be collected, one for Xpert MTB/RIF and one for MGIT culture (field or study clinic visit)

All participants will be given an appointment and contact details confirmed for the next study visit (Month 3)

VISIT 4: DAY 84 (+/- 3)
All participants will undergo the following assessments and procedures:

- Review of concomitant medications (ART and IPT)
- Measurement of weight
- Vital signs
- Targeted physical examination
- Blood will be collected for:
  - COR (Paxgene)
  - Serum for proteomics
  - And if recently started on ART
  - CD4 count (only in participants starting ART)
  - HIV viral load (only in participants starting ART)
- Urine will be collected for LAM assay and metabolomics
- Review of concomitant medications (including ART and IPT)
  - TB symptom screen
  - If indicated by one or more TB symptoms, two sputum samples will be collected, one for Xpert MTB/RIF and one for MGIT culture

All participants will be given an appointment and contact details confirmed for the next study visit (Month 6)

VISIT 5: DAY 180 (+/- 7)
All participants will undergo the following assessments and procedures:

- Review of concomitant medications (ART and IPT)
- Measurement of weight
- Vital signs
- Targeted physical examination
- TB symptom screen questionnaire
- If indicated by one or more TB symptoms, two sputum samples will be collected, one for Xpert MTB/RIF and one for MGIT culture

CONTACT 6: DAY 270 (+/- 7)
All participants will undergo the following assessments and procedures:

- Review of concomitant medications (ART and IPT)
- TB symptom screen
- If indicated by one or more TB symptoms, two sputum samples will be collected, one for Xpert MTB/RIF and one for MGIT culture (field or study clinic visit)

All participants will be given an appointment and contact details confirmed for the next study visit (Month 12)

VISIT 7: DAY 365 (+/- 7)
All participants will undergo the following assessments and procedures:

- Review of concomitant medications (ART and IPT)
- Measurement of weight
- Vital signs
- Targeted physical examination
- TB symptom screen
- If indicated by one or more TB symptoms, two sputum samples will be collected, one for Xpert MTB/RIF and one for MGIT culture

All participants will be given an appointment and contact details confirmed for the next study visit (Month 15)

VISIT 9 (END-OF-STUDY VISIT): DAY 449 (+/- 7)
All participants will return to the study site for a final end-of-study evaluation. Every attempt will be made to ensure that participants are not lost to follow-up prior to this visit and the study team will attempt to trace participants who fail to present for this visit.

All participants will undergo the following assessments and procedures:
Review of concomitant medications (ART and IPT)
Measurement of weight
Vital signs
Targeted physical examination
TB symptom screen
Two sputum samples will be collected, regardless of presence or absence of symptoms, one for Xpert MTB/RIF and one for MGIT culture.
Contact details will be confirmed so that sputum results can be provided, with written TB clinic referral if necessary

EARLY WITHDRAWAL FROM THE STUDY
Participants will be advised that they are free to withdraw from the study at any time, for any reason, without prejudice. Every reasonable effort should be made by the study staff to keep participants in the study. Participants must, however, be withdrawn from the study for any of the following reasons

- At the request of the participant (withdrawal of informed consent), irrespective of the reason
- At the discretion of the investigator if he or she believes that continuation in the study would be detrimental to the participant's well-being

For participants who are lost to follow-up, study personnel should make at least three documented attempts to contact the participant. Determination of loss to follow-up will be made at end of study, if and when a participant does not return to follow-up after a missed visit/s. Unless lost to follow-up, withdrawn participants will attend an early discontinuation visit (procedures and assessments conducted as for End of Study Visit). Reason for withdrawal from the study will be documented to ascertain the cause of early termination.

Participants who are diagnosed with TB and referred for curative treatment should attend an End of Study Visit at the time of their next scheduled study contact/visit to confirm that the participant has accessed the appropriate care, after which an End of Study Visit form should be completed.

STUDY ASSESSMENTS

SCREENING DATA
Screening data will be collected prior to enrolment.

AGE VERIFICATION
Age of potential participants at date of screening will be verified by identification document, passport, or driver's license, copy of which to be kept in the participant file and participant identity checked at each visit.

SCREENING MEDICAL HISTORY
Potential participants will provide a targeted medical history, with a focus on socio-demographic data (gender, ethnicity, education level, & household economic indicators), risk factors for TB (including TB contact and smoking history), current and past medical and surgical conditions (including recent febrile episodes), and concomitant medications (including ART and IPT).

WEIGHT AND HEIGHT
Height in centimetres (cm) and body weight (to the nearest 0.1 kg in indoor clothing, but without shoes) will be measured at screening. Weight only will be repeated at each subsequent study visit. Body Mass Index (BMI) will be calculated using the formula:
BMI = weight (kg) / height $(m)^2$.

URINE PREGNANCY TEST
To confirm eligibility at screening, a urine β-hCG test will be performed for all females of child-bearing potential.

HIV RAPID TEST
Following appropriate pre-test counselling, evaluation for HIV seropositivity will be performed by rapid test, and, if positive, will be confirmed by a second rapid test as per site protocol. Discordant rapid test results will be confirmed by laboratory HIV ELISA. Appropriate post-test counselling will be made available by the investigator, and participants will be referred for ongoing HIV management in the event of a positive test. A documented positive HIV test that has been performed for CORTIS-01 screening will not need to be repeated if performed within 28 days of CORTIS-HR screening.

VITAL SIGNS & PHYSICAL EXAMINATION
A targeted physical examination will be performed at enrolment, including recording of vital signs (temperature, pulse rate, blood pressure); general examination including lymph nodes and skin; detailed examination of the respiratory system; and other systems as indicated on the basis of medical history or other physical findings. At all other visits, vital signs and an abbreviated physical examination will be performed if directed by presence of symptoms or occurrence of adverse events. Physical examination data will be recorded in the source documentation at the trial site.

VERIFICATION OF ELIGIBILITY
The investigator will review the screening medical history and tests and confirm eligibility.

COR ASSAY
Whole blood RNA will be collected in PAXgene tubes from all eligible persons at enrolment and repeated at Month 3 (1 tube of 2.5mL blood, stored at room temperature for 2-18 hours, then frozen at -20°C) and shipped frozen to the South African Tuberculosis Vaccine Initiative (SATVI) Cape Town Laboratory on a weekly basis. PAXgene tubes will be processed in batches of 94 (plus 2 internal assay controls), allowing for handling by standard assay plate format. cDNA synthesis and pre-amplification will be automated, and up to 6 Fluidigm chips will be run weekly. In order to extract high quality RNA and complete the first amplification steps of the COR assay using whole blood collected in PAXgene tubes in a high-throughput, standardized, reproducible and cost effective manner, a fully automated procedure using the TECAN EVO Freedom robotic platform will perform RNA extraction, cDNA-synthesis and pre-amplification steps on up to 465 samples per week. Participants will be evaluated for risk of TB disease using the PSVM.1 model on the BioMark HD Fluidigm multiplex qRT-PCR machine. COR analysis will be conducted by a locked-down R script, which includes Quality Control filters. COR data will not be used to guide clinical care.

IGRA
A 4mL whole blood sample will be collected for IGRA on Day 0 in all enrolled participants (4mL total, incubated at 37°C for 16-24 hours, then spun and supernatants stored at -80°C). The Day 0 IGRA result will be made available to the health services to guide clinical management.

SERUM PROTEOMICS
A 5mL clotted blood sample will be collected on Day 0 in all enrolled participants and repeated at Month 3. Serum will be spun and stored at -80°C for future proteomic analysis. Serum may be stored for up to 10 years for proteomic analysis before being destroyed

PLASMA MOLECULAR ASSAYS
A 6mL EDTA blood sample will be collected on Day 0 in all enrolled participants. Plasma will be stored at -80°C for viral assays, including molecular tests for HIV and other viral pathogens. Plasma may be stored for up to 10 years for molecular assays before being destroyed.

CD4 CELL COUNT
A 4mL EDTA blood sample will be collected for CD4 cell count on Day 0 in all enrolled participants and repeated at Month 3 only in those participants referred for ART. CD4 cell count results at Day 0 and Month 3 will be made available to health service providers to guide clinical care.

HIV VIRAL LOAD
A 6mL EDTA blood sample will be collected for HIV viral load on Day 0 only in those participants not yet established on ART and repeated at Month 3 after ART has been started. HIV viral load test results at Day 0 and Month 3 will be made available to health service providers to guide clinical care.

URINE LIPOARABINOMANNAN (LAM) AND METABOLOMICS
Urine (20mL) will be collected for urinary LAM assay and two aliquots of urine will be stored for specific protein and metabolomic studies. Urine may be stored for up to 10 years for analysis before being destroyed. Results of urinary LAM assays will not be used to guide clinical care in study participants, who will be non-hospitalized community volunteers.

TB EVALUATIONS
Investigation for TB disease will be conducted in all participants at Visit 1 (Day 0); in symptomatic participants during follow-up; and in all participants at the end of study visit.

TB SYMPTOM SCREENING
All participants will be asked about any new household or other close contact with a recently diagnosed TB patient, and about symptoms consistent with TB disease, at all study contacts and visits from Day 0 through end of study. Symptoms will include loss of weight or persistent unexplained cough, chest pain, fever, or night sweats for longer than two weeks; or any haemoptysis.

TB INVESTIGATIONS
Two sputum samples will be collected from all participants at Visit 1 (Day 0), regardless of presence or absence of symptoms, for Xpert MTB/RIF testing and MGIT culture. An aliquot of unprocessed sputum from each sample will be stored for additional MTB diagnostic tests at the end of the trial, potentially including, but not limited to Xpert MTB/RIF, MGIT culture, and line probe assay. Thereafter, TB investigation will be symptom-triggered throughout follow-up. A participant with any one or more symptoms consistent with TB disease will be asked to provide two sputum samples, one for Xpert MTB/RIF and one for MGIT culture. An additional MGIT culture will be requested if only one sample is positive for either test. If a participant cannot produce a sputum sample spontaneously, this will be recorded in the source notes and no sample will be sent to the laboratory. Participants who are unproductive of sputum will be deemed Xpert MTB/RIF and MGIT culture negative. However, if clinical suspicion of TB disease persists in any participant, additional samples may be obtained, including induced sputum, or samples from other sites in the case of suspected extra-pulmonary disease. All participants will provide two sputum samples for Xpert MTB/RIF testing and MGIT culture at the end of study visit.

STATISTICAL CONSIDERATIONS

We have estimated the expected number of prevalent and incident TB cases in HIV infected participants in CORTIS-HR, based on data from previous and ongoing studies in South Africa. In order to estimate the expected number of TB cases and a probable range, we used stochastic simulations of the CORTIS-HR study under a range of assumptions. The simulation assumed a 10% HIV prevalence rate, ranging between 5% - 25%, which would allow 860 HIV infected participants to be enrolled from approximately 10,000 persons screened for the CORTIS trial and the CORTIS-HR study.

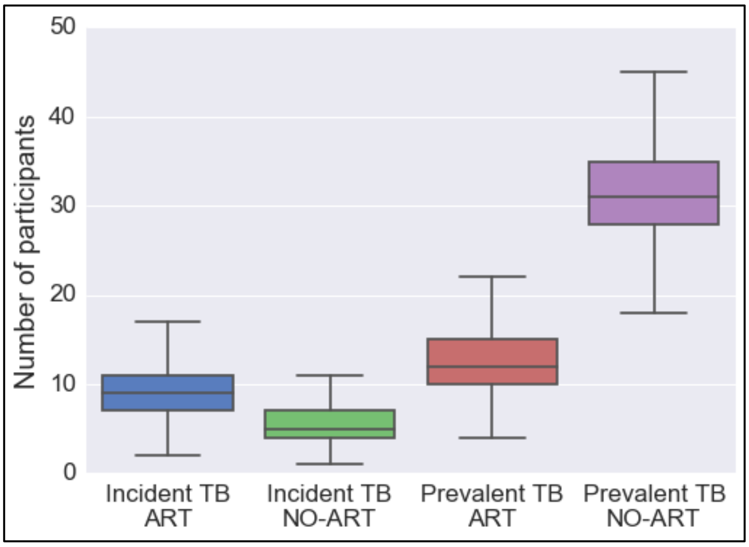

Figure 1: Expected number of incident and prevalent TB endpoints among HIV infected participants. Estimates are based on stochastic simulations.

We estimate that 75% of these participants will be receiving chronic ART and the remainder will begin ART. Based on studies conducted in South Africa, the rate of prevalent TB will be considerably higher among participants newly starting ART (15%) [21, 22], compared to those receiving chronic ART (2%) [18-22]. We also assume that 15% of participants on chronic ART will also be on IPT, thus further reducing the expected level of prevalent TB by 40%. Similarly, we expect the annual rate of incident TB will be higher among participants newly starting ART (4%) [21, 22], than among those already established on chronic ART (2%) [20]. In both these groups, we expect up to 70% to begin IPT upon enrollment, which will reduce expected levels of incident TB by 40%.

Based on these pre-specified parameter distributions, we expect that of the 215 newly diagnosed HIV infected participants yet to start ART, we will identify 31 prevalent TB cases (95% CI 22, 42) at screening; and an additional 5 (95% CI 2, 10) participants who will develop active TB disease over 15 months of follow-up. We expect that of the 645 HIV infected participants already on chronic ART, we will identify 12 prevalent TB cases (95% CI 6, 20) at screening; and an additional 9 (95% CI 4, 15) incident TB cases over 15 months of follow-up. Thus, we expect to measure the COR in a total of 58 (95% CI 44, 73) cumulative endpoint TB cases (43 prevalent and 14 incident TB cases) and 802 (95% CI 787, 816) controls.

We will evaluate the relative-risk of endpoint-defined TB over 15 months, $RR_{COR}(15)$, in COR+ versus COR- participants using a cumulative incidence based approach. The primary analysis will evaluate $RR_{COR}(15)$ on endpoints using the two-sample endpoint definition and according to the formula:

$$RR_{COR}(15) = \frac{H_A}{H_B}$$

where H_X is the cumulative incidence estimated for each group using the Product-Limit estimator of Nelson-Aalen (Aalen, O.O., “Non-parametric inference for a family of counting processes”, Annals of Statistics, 1978). A point-estimate for $RR_{COR}(15)$ will be presented with 90% confidence intervals and a p-value for the null-hypothesis H_0: $RR_{COR}(15) <= 2$. Descriptive plots will include Kaplan-Meier estimators with 95% confidence intervals for each group. In addition we will present time-dependent estimates of sensitivity, specificity, positive predictive value (PPV) and number needed to treat (NNT) using the methods of Heagerty et al. (2000, Biometrics, “Time-dependent ROC Curves for Censored Survival Data and a Diagnostic Marker”) as these will offer important insights into the performance and application of the COR in future strategies.

Our preliminary data suggests that the COR will perform similarly in HIV infected participants, with a possible 10% reduction in sensitivity to 77%, compared to HIV uninfected persons. Based on these characteristics, and the expected total number of cumulative TB disease endpoints (prevalent and incident), we estimate that the Relative Risk (RR) of a COR+ HIV infected participant for cumulative TB disease, relative to a COR- HIV infected participant, will be approximately 19 (95% CI 11-34). **See Figure 1.** Corresponding estimates of precision for RR for prevalent cases only are 95% CI 10-37; and for incident cases only are 95% CI 6-65.

We will perform an interim analysis of COR prevalence and diagnostic performance for prevalent TB cases only, in order to inform a Stop/Go decision for continuation of study follow-up beyond 3 months in all subjects to evaluate COR prognostic performance. In subjects who continue follow-up for incident TB disease, we will perform a sensitivity analysis of month 3 COR prognostic performance to evaluate the impact of IPT and/or ART on COR score and TB risk.

MODELLING

Data from the CORTIS-HR study on the performance of the COR in HIV-infected individuals will be used to refine our preliminary models and to predict the population-level impact of COR 'screen and treat' strategies in HIV infected populations. The model will be used to explore the impact under alternative scenarios of ART rollout and uptake of IPT; and alternative assumptions about the efficacy of COR-targeted 3HP regimens in HIV infected individuals. Models will make use of screening data (including age of trial participants, prevalence of undiagnosed TB), diagnostic performance of COR (for prevalent and incident TB), and effect of potential confounders including CD4 count, HIV viral load, ART and IPT on COR score, ideal threshold value, and performance.

DATA HANDLING AND QUALITY ASSURANCE

As part of the responsibilities assumed by participating in the study, the investigator agrees to maintain adequate case histories for all participants under this protocol. This includes the maintenance of both source documentation and accurate electronic CRFs (eCRFs) for all participants who consent to participation in the study. Study data will be collected using a 21 CFR Part 11 compliant electronic system. Information recorded in the eCRFs will be supported by corresponding source documentation. All paper and electronic source documents pertaining to this study will be maintained by the investigators. eCRFs are considered confidential documents and will be handled and accessed accordingly. The Data Centre will provide the necessary training on the use of the specific eCRF system utilised during the study to ensure that data are captured accurately and appropriately. Each completed eCRF will be reviewed, signed, and dated by the investigator in a timely manner.

MONITORING THE STUDY

Clinical sites will be monitored by a clinical research associate (CRA) to ensure compliance with the protocol, GCP and applicable regulations and guidelines. The CRA(s) will conduct site visits and will be responsible for ensuring that the study protocol is adhered to. The assigned CRA(s) will visit the investigator and clinical site at periodic intervals and maintain periodic communication. The CRA(s) will maintain current personal knowledge of the study through observation, review of trial records and source documentation, and discussion of the conduct of the study with the investigator and staff. While on site, the CRA(s) will review all study, regulatory and ethics documents, compare entries in the CRF system with the source documents, and review endpoint laboratory sample storage. The CRA(s) will ask for clarification and/or correction of any noted inconsistencies. Any necessary corrections will be made in such a way that the original entry, the date of the correction and the identity of the person making the correction is accessible. By signing the protocol, the Investigator agrees to meet with the CRA(s) during clinical site visits, to ensure that site staff are available to the CRA(s) as needed, to provide the CRA(s) access to all study documentation and agrees to assist the monitors in their activities, if requested.

DATABASE MANAGEMENT AND QUALITY CONTROL

Data management, including the development and management of a database, will be performed in accordance with regulatory requirements by the Triclinium Clinical Development (TCD) Data Centre. Triclinium will review the eCRF data for completeness and accuracy. A formal querying process will be followed whereby the data management group will request the site personnel to clarify any apparent erroneous entries or inconsistencies and will request additional information from the site as required. Medical history/current medical conditions will be coded using the Medical dictionary for regulatory activities (MedDRA, version 18.1 or higher) terminology. Concomitant medications will be coded using the MIMS classification.

After all data have been captured and reviewed in the eCRF, all queries have been resolved with the site and any protocol non-compliances that were identified during the data management processes have been confirmed by the site, the database will be declared to be complete and accurate. It will then be locked and the COR status of participants in the Observation Arm will be unblinded and made available for data analysis. Any changes to the database after that time may only be made by the data manager, in consultation with the sponsor and in accordance with documented database unlock and relock procedures. Data management procedures will be described in detail in the Data Management Plan which will be documented and approved prior to study start.

INSPECTION OF RECORDS

Investigators and institutions involved in the study will permit study-related monitoring, audits, IEC review, and regulatory inspection(s) by providing direct access to all study records. The confidentiality of records that can identify participants will be protected, respecting the privacy and confidentiality rules in accordance with regulatory requirements. The investigator must promptly

notify the sponsor of any audits scheduled by any regulatory authorities and promptly forward copies of any audit reports received to the sponsor.

RETENTION OF RECORDS

Essential documents should be retained for not less than 10 years after study completion. These documents should be retained for a longer period, however, if required by the applicable regulatory requirements or by an agreement with the sponsor. It is the responsibility of the sponsor to inform the investigator when these documents no longer need to be retained.

ETHICAL CONSIDERATIONS

REGULATORY AND ETHICAL COMPLIANCE

This study will be conducted according to the ethical principles set forth in the Declaration of Helsinki (Fortaleza, Brazil 2013),[21] ICH-GCP,[22] European Directive 2001/20/EC,[23] US Code of Federal Regulations Title 21,[24] South African Good Clinical Practice Guidelines,[25] and other local regulatory requirements.

INFORMED CONSENT PROCEDURES

Eligible participants may only be included in the study after providing written, IEC-approved informed consent in the language of their choice. Illiterate participants require an impartial witness to be present for the informed consent process, and to sign and date that all information in the informed consent form was shared with participant, and the participant must sign by means of a thumbprint. Informed consent must be obtained before conducting any study-specific procedures (i.e. any of the procedures described in the protocol). The timing and process of obtaining informed consent must be documented in the participant source documents. The investigator must provide a copy of the signed informed consent document to the participant. The original form must be maintained together with the participant's source documents at the site.

RESPONSIBILITIES OF THE INVESTIGATOR AND IEC

The protocol and informed consent form must be reviewed and approved by a properly constituted IEC before commencement of the study at any site. By signing this study protocol, the principal investigator agrees to conduct this study in accordance with all laws, regulations and guidelines of the pertinent regulatory authorities, including the 2013 Version of the Declaration of Helsinki. While delegation of certain aspects of the study to sub-investigators and study coordinators is appropriate, the principal investigator will remain personally accountable for overseeing the study and for ensuring compliance with the protocol and all applicable regulations and guidelines.

The PI must ensure that all persons who have been delegated study-related responsibilities are adequately qualified and informed about the protocol and their specific duties within the context of the study. The principal investigator is responsible for providing the sponsor with documentation of the qualifications, GCP training, and research experience of site staff as required by the sponsor and the relevant governing authorities. In addition to this, the principal investigator is responsible for maintaining a list of all personnel who have been trained and to whom study-related responsibilities have been delegated, including the specific study-related duties concerned. Proof of training on the protocol and protocol-specific procedures must be kept on file to allow verification of training for delegated duties.

PROTOCOL ADHERENCE

A protocol non-conformance is an unintended and/or unanticipated departure from the procedures and/or processes approved by the sponsor, the IEC, and agreed to by the investigator. Investigators must agree to apply due diligence to avoid protocol non-conformances and must document and explain any such events. Protocol non-conformances will also be documented by the CRA throughout the course of monitoring visits. The investigator will be notified of non-conformances in writing by the CRA. The IEC must be notified of major protocol non-conformances in accordance with the IEC standard operating procedures.

AMENDMENTS TO THE PROTOCOL

Any change or addition to the protocol can only be made by means of a written protocol amendment that is approved by the sponsor and the IEC. Notwithstanding the need for approval of formal protocol amendments, the investigator is expected to take any immediate action required for the safety of any participant included in this study, even if this action represents a deviation from the protocol. In such cases, the sponsor should be notified of this action immediately and the IEC at the study site should be informed within 10 working days of the action.

PARTICIPANT INJURY
The sponsor (University of Cape Town) will ensure that provisions are made for insurance or indemnity by a third party to cover the liability of the investigator and sponsor in relation to the study. In the event of any injury, suffering, deterioration in health or well-being resulting from a participant's participation in the study, the participant will receive appropriate compensation irrespective of their ability to prove fault on the part of the sponsor or anyone else connected with the study.

SAMPLE RETENTION
Biological samples may only be used for purposes related to this research. The samples will be stored until the study team has determined that specimens are no longer needed and the decision has been made that there are no samples to be re-assayed, up to a maximum period of 10 years. In addition, identifiable samples can be destroyed at any time at the request of the participant.

STUDY TERMINATION BY SPONSOR
The study may be terminated at the sponsor's discretion at any time for any reason. If the sponsor discovers conditions that warrant early termination of the study, the investigator will be notified by the sponsor or by its designee.

CLINICAL SITE CLOSURE
On termination of the study, all screening and ongoing study-related procedures conducted at the study site will be closed. The sponsor may terminate participation of the clinical site at any time. Examples of conditions that may warrant premature termination of a clinical site include, but are not limited to non-compliance with the protocol and/or applicable regulations and guidelines, or inadequate participant enrolment.

PUBLICATION OF THE STUDY PROTOCOL AND RESULTS
All information concerning the sponsor's operations, patent applications, formulae, and scientific data supplied by the sponsor to the investigator and not previously published, is considered confidential and remains the sole property of the sponsor. The complete participant CRFs also remain the property of the sponsor. The investigator agrees to use this information for purposes of the study execution only. The sponsor will post the key design elements of this protocol in a publicly accessible database such as clinicaltrials.gov. In addition, upon study completion the results of this study will be submitted for publication and/or posted in a publicly accessible database. Publications or other public presentations of the data resulting from this study will be planned and prepared by a Writing Committee chaired by the PI and including the study investigators.

REFERENCES

1. Zak DE, Penn-Nicholson A, Scriba TJ, et al. A blood RNA signature for tuberculosis disease risk: a prospective cohort study. *The Lancet* 2016.
2. Berry MP, Graham CM, McNab FW, et al. An interferon-inducible neutrophil-driven blood transcriptional signature in human tuberculosis. *Nature* 2010; **466**(7309): 973-7.
3. Bloom CI, Graham CM, Berry MP, et al. Transcriptional blood signatures distinguish pulmonary tuberculosis, pulmonary sarcoidosis, pneumonias and lung cancers. *PloS one* 2013; **8**(8): e70630.
4. Bloom CI, Graham CM, Berry MP, et al. Detectable changes in the blood transcriptome are present after two weeks of antituberculosis therapy. *PloS one* 2012; **7**(10): e46191.
5. Kaforou M, Wright VJ, Oni T, et al. Detection of tuberculosis in HIV-infected and -uninfected African adults using whole blood RNA expression signatures: a case-control study. *PLoS medicine* 2013; **10**(10): e1001538.
6. WHO. Global Tuberculosis Control Report. 2015.
7. del Amo J, Moreno S, Bucher HC, et al. Impact of antiretroviral therapy on tuberculosis incidence among HIV-positive patients in high-income countries. *Clinical infectious diseases : an official publication of the Infectious Diseases Society of America* 2012; **54**(9): 1364-72.
8. Suthar AB, Lawn SD, del Amo J, et al. Antiretroviral therapy for prevention of tuberculosis in adults with HIV: a systematic review and meta-analysis. *PLoS medicine* 2012; **9**(7): e1001270.
9. Bachmann MO, Timmerman V, Fairall LR. Effect of antiretroviral treatment on the risk of tuberculosis during South Africa's programme expansion. *AIDS* 2015; **29**(17): 2261-8.
10. Lawn SD, Myer L, Edwards D, Bekker LG, Wood R. Short-term and long-term risk of tuberculosis associated with CD4 cell recovery during antiretroviral therapy in South Africa. *AIDS* 2009; **23**(13): 1717-25.
11. Rangaka MX, Wilkinson RJ, Boulle A, et al. Isoniazid plus antiretroviral therapy to prevent tuberculosis: a randomised double-blind, placebo-controlled trial. *Lancet* 2014; **384**(9944): 682-90.
12. Akolo C, Adetifa I, Shepperd S, Volmink J. Treatment of latent tuberculosis infection in HIV infected persons. *Cochrane Database Syst Rev* 2010; (1): CD000171.
13. Golub JE, Astemborski J, Ahmed M, et al. Long-term effectiveness of diagnosing and treating latent tuberculosis infection in a cohort of HIV-infected and at-risk injection drug users. *Journal of acquired immune deficiency syndromes* 2008; **49**(5): 532-7.
14. Golub JE, Cohn S, Saraceni V, et al. Long-term protection from isoniazid preventive therapy for tuberculosis in HIV-infected patients in a medium-burden tuberculosis setting: the TB/HIV in Rio (THRio) study. *Clinical infectious diseases : an official publication of the Infectious Diseases Society of America* 2015; **60**(4): 639-45.
15. Sumner T, Houben RM, Rangaka MX, et al. Post-Treatment Effect of Isoniazid Preventive Therapy on Tuberculosis Incidence in Hiv-Infected Individuals on Antiretroviral Therapy. *AIDS* 2016.
16. Samandari T, Agizew TB, Nyirenda S, et al. 6-month versus 36-month isoniazid preventive treatment for tuberculosis in adults with HIV infection in Botswana: a randomised, double-blind, placebo-controlled trial. *Lancet* 2011; **377**(9777): 1588-98.
17. Samandari T, Agizew TB, Nyirenda S, et al. Tuberculosis incidence after 36 months' isoniazid prophylaxis in HIV-infected adults in Botswana: a posttrial observational analysis. *AIDS* 2015; **29**(3): 351-9.
18. Kranzer K, Lawn SD, Meyer-Rath G, et al. Feasibility, yield, and cost of active tuberculosis case finding linked to a mobile HIV service in Cape Town, South Africa: a cross-sectional study. *PLoS medicine* 2012; **9**(8): e1001281.
19. Kufa T, Mngomezulu V, Charalambous S, et al. Undiagnosed tuberculosis among HIV clinic attendees: association with antiretroviral therapy and implications for intensified case finding, isoniazid preventive therapy, and infection control. *J Acquir Immune Defic Syndr* 2012; **60**(2): e22-8.
20. Kufa T, Mabuto T, Muchiri E, et al. Incidence of HIV-associated tuberculosis among individuals taking combination antiretroviral therapy: a systematic review and meta-analysis. *PLoS One* 2014; **9**(11): e111209.

21. Lawn SD, Kranzer K, Edwards DJ, McNally M, Bekker LG, Wood R. Tuberculosis during the first year of antiretroviral therapy in a South African cohort using an intensive pretreatment screening strategy. *AIDS* 2010; **24**(9): 1323-8.
22. Naidoo K, Karim QA, Bhushan A, et al. High rates of tuberculosis in patients accessing HAART in rural South Africa. *J Acquir Immune Defic Syndr* 2014; **65**(4): 438-46.
23. Mahomed H, Hughes EJ, Hawkridge T, et al. Comparison of mantoux skin test with three generations of a whole blood IFN-gamma assay for tuberculosis infection. *Int J Tuberc Lung Dis* 2006; **10**(3): 310-6.
24. Mahomed H, Hawkridge T, Verver S, et al. Predictive factors for latent tuberculosis infection among adolescents in a high-burden area in South Africa. *Int J Tuberc Lung Dis* 2011; **15**(3): 331-6.
25. Mahomed H, Hawkridge T, Verver S, et al. The tuberculin skin test versus QuantiFERON TB Gold(R) in predicting tuberculosis disease in an adolescent cohort study in South Africa. *PLoS One* 2011; **6**(3): e17984.
26. Mahomed H, Ehrlich R, Hawkridge T, et al. Screening for TB in high school adolescents in a high burden setting in South Africa. *Tuberculosis (Edinb)* 2013; **93**(3): 357-62.
27. Mahomed H, Ehrlich R, Hawkridge T, et al. TB incidence in an adolescent cohort in South Africa. *PloS one* 2013; **8**(3): e59652.

Statistical Analysis Plan:

11-gene Correlates of Risk (COR) Diagnostic and Predictive Performance Analysis in HIV-Infected Adults

Protocol Title: Validation of Correlates of Risk of TB Disease in High Risk Populations (CORTIS-HR)

A companion study of the CORTIS-01 Trial

Protocol Number: CORTIS-HR

Authors: Simon C Mendelsohn[1], MBChB

Andrew Fiore-Gartland[2], Ph.D.

Bhavesh Borate[2], M.S.

[1]South African Tuberculosis Vaccine Initiative, Department of Pathology and Institute of Infectious Diseases and Molecular Medicine, University of Cape Town

[2]Statistical Center for HIV/AIDS Research and Prevention, Vaccine and Infectious Disease Division, Fred Hutchinson Cancer Research Center

Date: 11 December 2019

Version: v 1.0

This document describes the primary statistical analysis plan to be performed on the data obtained from the CORTIS-HR study and is to be read in conjunction with the approved study protocol version 1.0 dated 26th August 2016.

**Document:**	Statistical Analysis Plan: 11-gene Correlates of Risk (COR) Diagnostic and Predictive Performance Analysis in HIV-Infected Adults. Final version 1.0 (Dated 11 December 2019)
**Protocol Title:**	Validation of Correlates of Risk of TB Disease in High Risk Populations (CORTIS-HR)<br>*A companion study of the CORTIS-01 Trial*
**Protocol Number:**	CORTIS-HR
**Authorised by:**	Prof Mark Hatherill<br>Director, South African Tuberculosis Vaccine Initiative, University of Cape Town

Sign: ________________________________

Date: ________________________________

Dr Andrew Fiore-Gartland

Vaccine and Infectious Disease Division, Fred Hutchinson Cancer Research Center

Sign: ________________________________

Date: ________________________________

Table of Contents

1 List of Abbreviations ___ ***5***

2 Study Overview ___ ***6***

Aims ___ 6

2.1 Study Design ___ **7**

2.2 Study Population ___ **7**

3 Statistical Considerations ___ ***8***

3.1 Statistical software ___ **8**

3.2 Responsibility ___ **8**

3.3 Blinding ___ **8**

3.4 Database lock and storage ___ **8**

3.5 Projected enrolment, case accrual and power ___ **8**

3.6 COR predictive performance analysis ___ **10**

3.7 TB disease endpoint adjudication algorithm ___ **10**

Raw endpoint data ___ 11

Study endpoints, censoring and cohort definitions ___ 12

3.8 Statistical methods ___ **14**

3.8.1 Methods for primary, secondary, and exploratory aims ___ 14

3.8.1 Classification of participants by endpoint/censoring status ___ 16

3.9 Tables and figures ___ **17**

3.9.1 Descriptive analyses ___ 17

3.9.2 COR and QFT performance analyses ___ 17

4 Tables ___ ***19***

4.1 Participant disposition (consort diagram) ___ **19**

4.2 Endpoint accrual ___ **20**

4.3 Enrolment and endpoint accrual by COR, ART and IPT status ___ **21**

4.4 Enrolled participant demographics and clinical characteristics, by site ___ **22**

4.5 Enrolled participant demographics and clinical characteristics By case status with crude odds ratio ___ **26**

4.6 Primary and secondary outcomes tables (BINARY ANALYSIS) ___ **31**

4.6.1 Cumulative test performance for primary end-point (two-sample) prevalent and incident TB cases during follow-up (Primary aim) ___ 31

4.6.2 Cumulative test performance for combined primary and secondary end-point (one- or two-sample) prevalent and incident TB cases during follow-up (Primary aim) 31
4.6.3 Screening/triage test performance for primary end-point (two-sample) prevalent TB cases at enrolment (Secondary aim 1) 31
4.6.4 Screening/triage test performance for combined primary and secondary end-point (one- or two-sample) prevalent TB cases at enrolment (Secondary aim 1) 31
4.6.5 Predictive test performance for primary end-point (two-sample) incident TB cases during follow-up 31
4.6.6 Predictive test performance for combined primary and secondary end-point (one- or two-sample) incident TB cases during follow-up 31

4.7 Performance of the COR (RISK11) and QFT for identification of TB disease over a 15-month period, stratified by the time interval to disease (Time-dependent analysis) 34

4.7.1 Cumulative test performance for primary end-point (two-sample) prevalent and incident TB cases during follow-up (Primary aim) 34
4.7.2 Cumulative test performance for combined primary and secondary end-point (one- or two-sample) prevalent and incident TB cases during follow-up (Primary aim) 34
4.7.3 Predictive test performance for primary end-point (two-sample) incident TB cases during follow-up (Secondary aims 2 and 3) 34
4.7.4 Predictive test performance for combined primary and secondary end-point (one- or two-sample) incident TB cases during follow-up (Secondary aims 2 and 3) 34

5 Figures 35

5.1 Types of Figures 35

5.1.1 Test performance: ROC curves (sensitivity versus 100-specificity) 35
5.1.2 Test accuracy: sensitivity/specificity versus test score threshold plots (x-axis: score threshold, y-axis: sensitivity or specificity) 35
5.1.3 Test score distribution: violin/box-and-scatter plots (Prevalent and incident TB cases versus TB negative controls) 35
5.1.4 Time dependent analysis: AUC versus time. CIR versus time. Sensitivity, specificity, NPV and PPV (at different thresholds) versus time, over 15-month follow-up 35

5.2 Endpoints for figures 35

5.3 Tests 35

5.3.1 COR (RISK11) 35
5.3.2 QuantiFERON 35

6 Appendix 36

6.1 WHO diagnostic, triage, and predictive TB test performance target product profile 36

6.2 Concomitant medication coding 37

7 References 43

1 LIST OF ABBREVIATIONS

AFB	Acid-fast bacilli
BMI	Body mass index
cDNA	Complementary DNA
CI	Confidence interval
CIR	Cumulative incidence ratio
COR	Correlate of risk
CRA	Clinical research associate
CRF	Case report form
eCRF	Electronic CRFs
GCP	Good clinical practice
H_0	Null hypothesis
HIV	Human immunodeficiency virus
IEC	Independent ethics committee
IGRA	Interferon gamma release assay
INH	Isoniazid
IPT	Isoniazid preventive therapy
LAM	Lipoarabinomannan
LTBI	Latent tuberculosis infection
MDR-TB	Multi-drug resistant tuberculosis
MGIT	Mycobacteria growth indicator tube
mRNA	Messenger RNA
Mtb	*Mycobacterium tuberculosis*
OR	Odds ratio
NNS	Number needed to screen (to detect one case)
NPV	Negative predictive value
PI	Principal investigator
PPV	Positive predictive value
QFT	QuantiFERON
RR	Relative risk
$RR_{COR}(15)$	Relative risk for TB disease over 15 months
SA	South Africa
SATVI	South African Tuberculosis Vaccine Initiative
TB	Tuberculosis
TCD	Triclinium Clinical Development
TST	Tuberculin skin test
WHO	World Health Organization

2 STUDY OVERVIEW

There is a need for earlier TB case identification, using novel non-sputum based diagnostics, linked to more effective preventive and curative strategies (World Health Organization, 2015). A blood-based triage test that allows targeted investigation for active and sub-clinical TB disease, including asymptomatic individuals at highest risk of progression from latency to disease, could shorten the time to TB treatment, or even prevent disease before symptoms emerge. The tuberculin skin test (TST) and interferon gamma release assay (IGRA) have poor specificity for incident TB disease in endemic populations, including HIV infected people (Auguste, *BMC Infect Dis*, 2017).

We have previously developed a highly specific predictive correlate of risk (COR) to identify healthy, HIV uninfected, South African adults at high risk of active TB disease (Zak, *Lancet*, 2016). This validated COR, based on mRNA expression signatures in blood, prospectively discriminates between TB cases and healthy controls among HIV uninfected persons. Based on published microarray case-control datasets, the COR has 87% diagnostic sensitivity and 97% specificity for prevalent TB disease in HIV uninfected South African adults (Zak, *Lancet*, 2016); and in two nested case-control studies, also among HIV uninfected persons, the COR has 70% predictive sensitivity and 84% specificity for incident TB disease occurring within one year of sampling (Penn-Nicholson, *S Afr Med J*, 2016). This PCR-based mRNA COR signature has been refined to 11-genes with equivalent diagnostic performance (Darboe, *Tuberculosis*, 2018).

COR diagnostic performance for discriminating prevalent TB disease from latent TB infection in HIV infected persons appears to be reduced by approximately 10%, compared to HIV uninfected persons (Darboe, *Front Microbiol*, 2019). The overarching objective of this study is to evaluate the performance of the 11-gene mRNA COR signature to identify prevalent TB disease and predict incident TB disease in HIV infected individuals.

AIMS

Primary aim

Test whether COR status differentiates HIV infected persons with cumulative prevalent or incident TB disease from those without TB disease.

Secondary aims

1. Estimate whether COR status differentiates HIV infected persons with prevalent TB disease from those without prevalent TB disease
2. Estimate whether COR status differentiates HIV infected persons at high risk for incident TB disease from those at low risk for incident TB disease
3. Compare predictive performance of the COR for incident TB disease with Interferon-gamma release assay (IGRA) in HIV infected persons.

Exploratory aims

(Not addressed in this statistical analysis plan)

1. Assess and model the impact of a COR screen & treat strategy on reducing the rate of incident TB disease and TB mortality among HIV infected persons in South Africa.
2. Re-parameterize the COR assay for prevalent and incident TB disease in HIV infected persons.
3. Test the performance of additional validated COR signatures in distinguishing HIV infected persons with cumulative prevalent or incident TB disease from those without TB disease.
4. Compare predictive performance of the COR for incident TB disease in HIV infected persons before and 3 months after starting IPT and/or ART.

2.1 STUDY DESIGN

CORTIS-HR is an observational study of COR diagnostic and predictive performance in HIV infected persons. Following sample collection for the COR assay at Visit 1 (Day 0), all participants will be assessed for prevalent TB with TB investigations. All eligible participants without prevalent TB will be referred for Isoniazid preventive therapy (IPT). All HIV infected persons eligible for ART, as per SA national guidelines, will be referred in writing to the clinic for ART. Thereafter, all participants will be followed for 15 months for incident TB disease (3 telephone contacts and 4 site visits). Symptoms consistent with TB disease will be solicited at study visits and presence of one or more symptoms will trigger TB investigation. TB investigations will also be performed on all participants at the end of study follow up (month 15). The performance of the COR will be evaluated by comparing the cumulative incidence of endpoint-defined TB disease over 15 months in COR+ versus COR- participants (RR_{COR}).

2.2 STUDY POPULATION

The study population will include 860 HIV infected adults residing in TB hyperendemic communities at five or more study sites in South Africa. Primarily, persons screened for the CORTIS-01 trial who are ineligible on the grounds of HIV infection will be approached by study staff to participate in CORTIS-HR. In addition, persons with documented HIV infection may be approached through local ART clinics and Voluntary Counselling & Testing (VCT) services affiliated with or run directly by the study sites. Although recruitment efforts will be focused on persons with documented HIV infection status, in order to maintain confidentiality and avoid risk of stigmatization, community volunteers with unknown HIV status would also be eligible for screening.

3 STATISTICAL CONSIDERATIONS

3.1 STATISTICAL SOFTWARE

Statistical analysis will be performed using statistical software including Prism (GraphPad Software Inc.) and R. Where significance is reported, the 5% level of significance will be used with correction for false discovery and multiple comparisons.

3.2 RESPONSIBILITY

Management of the clinical and laboratory data as outlined in the protocol will be the responsibility of the designated laboratory technologists and doctoral scientist under the supervision of the Deputy Director of Immunology, SATVI and the study PI and Director SATVI.

3.3 BLINDING

The researcher assigned to study oversight and statistical analysis will be blinded to participant COR scores, COR status, and QuantiFERON (QFT) results until this statistical analysis plan has been approved and signed by study sponsor. However, access to clinical and demographic data (include TB disease status) will available to allow data cleaning and preparation of analysis script prior to database lock.

3.4 DATABASE LOCK AND STORAGE

After completion of study follow up, acquisition of final sputum culture results, and completion of database cleaning by Triclinium Clinical Development (TCD), database will be locked by agreement of the study PI, sponsor, and analysis team. All files containing the final data will be password-protected and backed-up to the SATVI server under a "CORTIS-HR" folder. Data will be saved as a .csv file and compiled into one master database using R. All analysis scripts and outputs will also be backed up onto the SATVI server upon completion of the study report.

3.5 PROJECTED ENROLMENT, CASE ACCRUAL AND POWER

We estimated the expected number of prevalent and incident TB cases in HIV infected participants in CORTIS-HR, based on data from previous and ongoing studies in South Africa. In order to estimate the expected number of TB cases and a probable range, we used stochastic simulations of the CORTIS-HR study under a range of assumptions. The simulation assumed a 10% HIV prevalence rate, ranging between 5% - 25%, which would allow 860 HIV infected participants to be enrolled from approximately 10,000 persons screened for the CORTIS trial and the CORTIS-HR study.

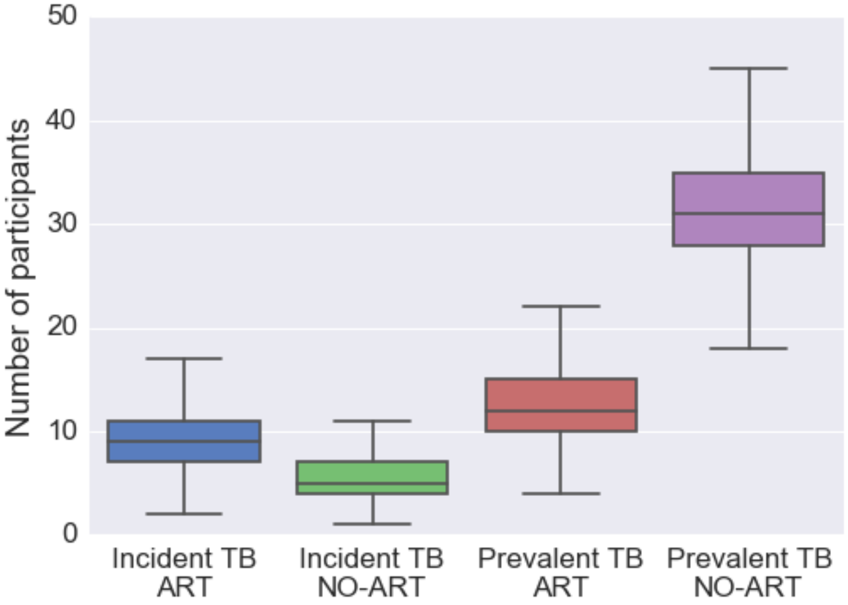

Figure 1: Expected number of incident and prevalent TB endpoints among HIV infected participants. Estimates are based on stochastic simulations.

We estimate that 75% of these participants will be receiving chronic ART and the remainder will begin ART. Based on studies conducted in South Africa, the rate of prevalent TB will be considerably higher among participants newly starting ART (15%), compared to those receiving chronic ART (2%). We also assume that 15% of participants on chronic ART will also be on IPT, thus further reducing the expected level of prevalent TB by 40%. Similarly, we expect the annual rate of incident TB will be higher among participants newly starting ART (4%), than among those already established on chronic ART (2%). In both these groups, we expect up to 70% to begin IPT upon enrolment, which will reduce expected levels of incident TB by 40%.

Based on these pre-specified parameter distributions, we expect that of the 215 newly diagnosed HIV infected participants yet to start ART, we will identify 31 prevalent TB cases (95% CI 22, 42) at screening; and an additional 5 (95% CI 2, 10) participants who will develop active TB disease over 15 months of follow-up. We expect that of the 645 HIV infected participants already on chronic ART, we will identify 12 prevalent TB cases (95% CI 6, 20) at screening; and an additional 9 (95% CI 4, 15) incident TB cases over 15 months of follow-up. Thus, we expect to measure the COR in a total of 58 (95% CI 44, 73) cumulative endpoint TB cases (43 prevalent and 14 incident TB cases) and 802 (95% CI 787, 816) controls.

3.6 COR PREDICTIVE PERFORMANCE ANALYSIS

In the CORTIS-01 study, a pre-defined COR score threshold of 60% was used to differentiate correlate positive (COR+) from correlate negative (COR-) individuals. There was no pre-defined COR threshold for HIV infected individuals, thus we plan to test multiple COR thresholds (See **Table 4.6**). For Secondary Aim 3, we will also assess multiple thresholds for the IGRA (See **Table 4.6**). To assess COR predictive performance for the prediction of incident TB disease we will evaluate the relative-risk of endpoint-defined TB over 15 months, $RR_{COR}(15)$, in COR+ versus COR- participants using a cumulative incidence-based approach. The primary analysis will evaluate $RR_{COR}(15)$ on endpoints using the two-sample endpoint definition and according to the formula:

$$RR_{COR}(15) = \frac{H_A}{H_B}$$

where H_X is the cumulative incidence estimated for each group using the Product-Limit estimator (Aalen, *The Annals of Statistics*, 1978). A point-estimate for $RR_{COR}(15)$ will be presented with 95% confidence intervals and a p-value for the null-hypothesis H_0: $RR_{COR}(15) \leq 1$. In addition we will present time-dependent estimates of sensitivity, specificity, positive predictive value (PPV) and number needed to treat (NNT) using the methods of Heagerty (*Biometrics*, 2000) as these will offer important insights into the performance and application of the COR in future strategies.

3.7 TB DISEASE ENDPOINT ADJUDICATION ALGORITHM

The algorithm is needed to classify each sputum sample as positive or negative and subsequently, each participant as TB-negative, two-sample positive (primary TB disease endpoint) or one-sample positive (secondary TB disease endpoint). It also establishes the study visit and timepoint at which TB-positive participants will be considered TB-positive.

The algorithm makes explicit two basic rules of adjudication: (1) Thirty-day episode window, and (2) “worst, first” case definition. The 30-day episode window implements the concept that assay results from multiple samples should be considered related and possibly combined to indicate a two-sample positive endpoint if they occur within 30-days of the first positive sample within the episode; samples collected more than 30 days apart should be considered as independent episodes for endpoint adjudication. The “first, worst” rule refers to the concept that a participant should be classified as a two-sample TB-positive endpoint if at any point during the study there is a two-sample positive episode, even if it follows a previous one-sample positive episode.

A window of 14 days will be applied to the end-of-study month 15 visit (i.e. a sputum collected ≥ month 15 + 14 days will be excluded).

RAW ENDPOINT DATA

Each participant in the study provides a number of samples and associated assay results. The visit number and collection dates are indicated for each sample by the BARC_MICRO.visit and BARC_MICRO.coll_dt, respectively. We consider two assays and their associated positive results below. GeneXpert MTB/RIF and Xpert Ultra "Trace" results are excluded from primary analysis.

Assay	Value of BARC_MICRO.proc	Value of BARC_MICRO.prompt	Value of BARC_MICRO.res indicating positive result
**Mycobacteria Growth Indicator Tube (MGIT)**	MGIT 960 Mycobacterial Culture	M tuberculosis :	Positive
	MGIT 960 Mycobact Re-Culture	M tuberculosis :	Positive
**GeneXpert MTB/RIF**	MTB PCR GENE EXPERT	Organism 1	Mycobact tuberculosis complex.
	MTB PCR GENE EXPERT ULTRA	Organism 1	Mycobact tuberculosis complex.
	MTB PCR GENE EXPERT ULTRA	Organism 1	Mycobact tuberculosis complexu

Apply the following steps to each participant's set of assay results, up until the time of the analysis:

Step 1. If there are no positive assay results the participant is classified as TB negative for all Study Visits. Proceed to adjudicate the next participant.

Step 2. Begin with the first Study Visit with an associated sample that is positive for either assay. This becomes the Episode Start Visit and starting date.

Step 3. If there are at least two samples collected at the Episode Start Visit that are positive for TB by either assay, then classify the participant as two-sample TB-positive at that visit. Proceed to adjudicate the next participant.

Step 4. If there is only one positive sample from the Episode Start Visit, examine all samples within a 30-day period. If there are any subsequent samples that are TB positive by either assay, then classify the participant as two-sample TB-positive at the Episode Start Visit. Proceed to adjudicate the next participant.

Step 5. If the participant has no subsequent TB-positive results, then classify the participant as one-sample TB-positive at the Episode Start Visit. Proceed to adjudicate the next participant.

Step 6. If the participant has TB-positive samples remaining, establish a new Episode Start Visit at the next positive sample (this should correspond to the next planned Study Visit). Continue with Step 3 to consider this new Episode.

STUDY ENDPOINTS, CENSORING AND COHORT DEFINITIONS

A participant classified as a one-sample or two-sample positive TB endpoint at the Enrolment Visit (Visit 1) will be classified as a prevalent TB case. Participants classified as a one-sample or two-sample positive TB endpoint at a subsequent visit will be defined as incident TB disease cases with right-censoring at the date of the positive sputum sample collection. Individuals who remain TB negative until study discontinuation, or end of follow up at the month 15 (day 449) end of study visit are classified as TB negative controls.

The primary and secondary aims will be addressed through time dependent and binary analyses of the intention to treat (ITT) cohort and modified ITT (mITT) cohorts:

The ITT cohort will include all participants, with censoring at discontinuation visit (e.g. unscheduled visit for pregnancy) or at last real visit (a real visit is one where a TB symptom screen was conducted or BARC microbiological data is available). For two-sample (primary endpoint), only consider two-sample endpoints as endpoints. One-sample endpoints will be censored at their last real visit. The ITT cohort will be used for addressing the primary aim (COR performance in differentiating cumulative prevalent and incident cases from TB negative controls) and secondary aim 1 (COR diagnostic performance in differentiating prevalent cases from combined TB negative controls and incident TB cases).

The mITT cohort excludes prevalent TB cases and will be used for assessing the performance of COR and QFT in predicting progression to TB disease (secondary aims 2 and 3).

Primary Analysis	Cohort	Cases	Controls
**Primary aim: Cumulative prevalent and incident cases (time-dependent endpoints)**	ITT	Incident and prevalent TB	TB negative
**Secondary aim 1: Diagnostic performance (binary endpoints)**	ITT	Prevalent TB	Incident TB and TB negative
**Secondary aims 2 and 3: Predictive performance (time-dependent endpoints)**	mITT (excluding prevalent cases)	Incident TB	TB negative

An exploratory analysis using binary endpoints will also be conducted and will only include participants who met a TB endpoint (prevalent or incident) or attended the month 15 end of study (EOS) visit without early discontinuation or left censoring.

Exploratory Analysis	Cohort	Cases	Controls
**Diagnostic performance (binary endpoints)**	Exclude early discontinuation and incident TB from ITT	Prevalent TB	TB negative
**Predictive performance (binary endpoints)**	Exclude early discontinuation and prevalent TB from ITT	Incident TB	TB negative

3.8 STATISTICAL METHODS

3.8.1 METHODS FOR PRIMARY, SECONDARY, AND EXPLORATORY AIMS

Endpoint*	Statistic	Relative-risk (COR+ vs. COR-, IGRA+ vs. IGRA-)			ROC statistics (COR, IGRA)			
		Cumulative ITT	Predictive (incident) mITT (exclude V1 TB)	Diagnostic ITT	Sensitivity	Specificity	AUC	Others (e.g. NNS, PPV, NPV)
Primary analysis: Time-dependent right censored	Statistic	CIR		-	*survAM.estimate* function, *survAccuracyMeasures* package (Zheng, 2016) estimation.method= IPW (non parametric, inverse-probability weighted)			
	95% CI	Wald-based		-	Bootstrapping, 10,000 replicates, non-stratified, non-parametric resampling			
	Test (p-value)	Wald-based<br>H_0: $CIR_{COR}(15) = 1$		-	-	-	-	-
Exploratory analysis: Binary[#]	Statistic	RR			pROC package (Robin, *BMC Bioinformatics*, 2011)			
	95% CI	Likelihood-score based (Koopman, *Biometrics*, 1984; Nam, *Biometrical Journal*, 1995)<br>PropCIs package (Scherer, 2018)			Percentile method (Carpenter, *Statistics in Medicine*, 2000),<br>10,000 bootstrap replicates with non-stratified, non-parametric resampling			
	Test (p-value)	Chi squared,<br>H_0: $RR_{COR}(15) = 1$			Comparing sensitivity/specificity of two tests:<br>(Pepe, *Stata J*, 2009), *roc.test* function, *pROC* package,<br>H_0: $Sensitivity_{COR} = Sensitivity_{IGRA}$<br>$H_0$: $Specificity_{COR} = Specificity_{IGRA}$		Single AUC: Mann-Whitney U (wilcox.test),<br>H_0: AUC < 0.5<br><br>Comparing AUCs: *roc.test* function (DeLong, *Biometrics*, 1988), *pROC* package,<br>H_0: $AUC_{COR} = AUC_{IGRA}$	-

*All analyses will be conducted twice: (I) Two-sample endpoints, (II) One and two-sample endpoints combined.

[#] In circumstances when there are 0 cases in one or more critical pieces of a binary endpoint evaluation (i.e. true positives or false negatives), 1 person will be added to each cell of the 2x2 table to help stabilise the estimate. This is particularly pertinent to the exploratory sub-group analyses: 1) ART-naïve versus ART experienced at baseline, 2) CD4 cell count at baseline <500, versus ≥ 500, 3) viral load at baseline lower than the detectable limit (LDL, <100), versus ≥ 100, 4) prior TB episode versus no prior TB episode, and 5) those who received IPT during study, versus no IPT during study. Other exploratory subgroups analyses will include age (<35, ≥35), sex (male/female), ethnicity (Black African, Cape Mixed Ancestry), study site, smoking history (yes/no), BMI (<18.5, ≥18.5), baseline IPT status (yes/no), and presence of TB symptoms (yes/no). For the RISK11 (COR) signature, an additional sub-group analysis of baseline QFT level will be performed (<0.35, ≥0.35; and <4, ≥4). These analyses may have limited power due to small sample size and limited numbers of active TB cases.

3.8.1 CLASSIFICATION OF PARTICIPANTS BY ENDPOINT/CENSORING STATUS

No. of samples	Endpoint?	V2 ENDP?	$T_{endpoint}/T_{LRV}$?	Assign T_{event}	Assign *endpoint*	Label
One (OS) or Two (TS) sample	Endpoint	≥V2	$T_{endpoint} \geq 15.5$*	T_{LRV-1} (LRV‡ prior to endpoint**)**	0	OS_ENDP_>V2_T>15.5
			$14.5 \geq T_{endpoint} < 15.5$	15	1	OS_ENDP_>V2_T15
			$T_{endpoint} < 14.5$	$T_{endpoint}$	1	OS_ENDP_>V2_T<14.5
		V1	-			OS_ENDP_V1
	Non-endpoint	-	$T_{LRV} \geq 14.5$	15	0	OS_NON_FULL
			$T_{LRV} < 14.5$	T_{LRV}	0	OS_NON
Two sample only	Same classification#, but only consider Two-sample endpoints as endpoints. One-sample endpoints will be censored at their LRV.					TS_ENDP_>V2_T>15.5
						TS_ENDP_>V2_T15
						TS_ENDP_>V2_T<14.5
						TS_ENDP_V1
						TS_NON_FULL
						TS_NON

‡LRV = last real visit, may include EOSM15 only if participant COMPLETED the study and was present at the EOSM15 visit, evidenced by presence of vital signs, TB screening, or BARC microbiological data

$T_{endpoint}$ = date determined from the endpoint adjudication process, specific to the endpoint (i.e. one- or two-sample)

T_{event} = elapsed time to endpoint or censor, for use in downstream analyses

*T > 15.5: a two-week visit window will be enforced for final visits that occur more than two weeks past 15 months. Also LRVs/endpoints occurring within two weeks before or after 15 months will be rounded to precisely 15 months to ensure a stable risk pool at the primary analysis timepoint

#Classification is valid if all ITT participants can be classified using one category for OS+TS endpoint and one category for TS endpoint

3.9 TABLES AND FIGURES

3.9.1 DESCRIPTIVE ANALYSES

Total number of subjects consented, screened, passing inclusion criteria, tested for COR, and enrolled as well as not enrolled into the trial will be summarized by site and COR status. Trial completion, trial withdrawals, exclusions and protocol non-compliances will also be summarized (**Table 4.1**).

Primary and secondary incident and prevalent endpoint accrual will be summarized by site and by COR status for subjects in the ITT cohort (**Table 4.2**).

Endpoint accrual based on two- and one-sample detection will also be summarized by ART and IPT treatment, and COR status (**Table 4.3**). ART and IPT status is determined by the concomitant medication recorded during follow up (**Appendix 6.2**).

Enrolled participant demographic, clinical and laboratory characteristics will be summarized by site (**Table 4.4**). Pearson's Chi-squared test (categorical data) or Kruskal-Wallis (continuous data) will be used to test for any statistically significant differences in demographic or baseline data between sites. A p value of <0.05 will be considered statistically significant.

Enrolled participant demographic, clinical and laboratory characteristics will also be summarized by case status (prevalent, incident and cumulative TB cases, and non-TB-endpoint controls) in the ITT cohort (**Tables 4.5**). Chi-squared test (categorical data) or logistic regression (continuous data) will be used to test for any statistically significant differences in demographic or baseline data between cases and controls. A p value of <0.05 will be considered statistically significant.

3.9.2 COR AND QFT PERFORMANCE ANALYSES

The performance of the COR and QFT (**primary and secondary aims**), measured at screening, will be evaluated on their ability to diagnose prevalent TB disease at baseline/enrolment and predict incident TB disease over 15 month follow up using the following performance measures: area under the receiver operating characteristic curve (AUC), relative-risk of COR+ vs. COR- (RR_{COR}), sensitivity, specificity, positive predictive value (PPV), negative predictive value (NPV), and the number needed to screen to detect one case (NNS).

COR performance will be compared with symptom screening, QFT, and urinary lipoarabinomannan (LAM) lateral flow assays also measured at screening. The estimates and confidence intervals will be provided in **Tables 4.6.1-6** alongside the minimum and optimal Target Product Profiles (TPP), provided by the WHO, for a community-based triage or referral test to identify people suspected of having TB (World

Health Organization, 2014, 2017). The WHO diagnostic, triage, and predictive TB test performance target product profiles (TPP) are included as **Appendix 6.1**.

Assessment of cumulative (primary aim), diagnostic (secondary aim 1), and predictive (secondary aim 2) performance of COR and QFT (secondary aim 3) will be based on only two-sample detection endpoints as well as both two- and one-sample detection endpoints (**Tables 4.6.1-6**). Different COR and QFT thresholds will be evaluated. ROC curves (**Figure 5.1.1**), sensitivity/specificity versus test score threshold plots (**Figure 5.1.2**), and violin/box-and-scatter plots (**Figure 5.1.3**) will be presented to describe COR and QFT performance at different cut-offs and to demonstrate score distributions.

Longitudinal performance of the COR and QFT assays (secondary aims 2 and 3) for identification of TB disease over a 15-month period based on only two-sample detection endpoints as well as both two- and one-sample detection endpoints, will be stratified by the time interval to disease (**Table 4.7.1-4** and **Figure 5.1.4**). The time dependent longitudinal analysis will allow estimation of AUC versus time, as well as sensitivity, specificity, NPV, and PPV (at a-priori and post-hoc thresholds) versus time, over 15-month follow-up. The optimal threshold will be set based on 75% test specificity (minimum WHO TPP for a predictive test for progression to TB disease).

Pre-specified exploratory diagnostic and predictive performance analyses (**Tables 4.6.1-6**, **Tables 4.7.1-4** and **Figures 5.1.1-5.1.4**) will also be performed by the following sub-groups: 1) ART-naïve versus ART experienced at baseline, 2) CD4 cell count at baseline <500, versus ≥ 500, 3) viral load at baseline lower than the detectable limit (LDL, <100), versus ≥ 100, 4) prior TB episode versus no prior TB episode, and 5) those who received IPT during study, versus no IPT during study. Other exploratory subgroups analyses will include age (<35, ≥35), sex (male/female), ethnicity (Black African, Cape Mixed Ancestry), study site, smoking history (yes/no), BMI (<18.5, ≥18.5), baseline IPT status (yes/no), and presence of TB symptoms (yes/no). For the RISK11 (COR) signature, an additional sub-group analysis of baseline QFT level will be performed (<0.35, ≥0.35; and <4, ≥4). These analyses may have limited power due to small sample size and limited numbers of active TB cases.

4 TABLES

4.1 PARTICIPANT DISPOSITION (CONSORT DIAGRAM)

Category	Subcategory		AIKlerks	AIRusten	CAPRISA	SATVI	SUN	Total
**Consented**			x	x	x	x	x	x
**Not screened**	*Out of enrolment window or did not arrive for enrolment visit*		x	x	x	x	x	x
**Screened**		N	x	x	x	x	x	x
**Excluded**		n (%)	x (%)	x (%)	x (%)	x (%)	x (%)	x (%)
	Aged <18 or ≥60	n (%)	x (%)	x (%)	x (%)	x (%)	x (%)	x (%)
	HIV negative	n (%)	x (%)	x (%)	x (%)	x (%)	x (%)	x (%)
	Pregnant or lactating	n (%)	x (%)	x (%)	x (%)	x (%)	x (%)	x (%)
	Unlikely to remain in follow up and adhere to protocol requirements	n (%)	x (%)	x (%)	x (%)	x (%)	x (%)	x (%)
	Diagnosed with TB disease within last 3 years	n (%)	x (%)	x (%)	x (%)	x (%)	x (%)	x (%)
	Household exposure to known MDR-TB patient within last 3 years	n (%)	x (%)	x (%)	x (%)	x (%)	x (%)	x (%)
	Any medical, surgical, or other condition likely to interfere with mRNA signature performance, safety or efficacy of ART and/or IPT, or adherence to study protocol	n (%)	x (%)	x (%)	x (%)	x (%)	x (%)	x (%)
	Missing inclusion data	n (%)	x (%)	x (%)	x (%)	x (%)	x (%)	x (%)
**Enrolled**		N	x	x	x	x	x	x
	COR+	n (%)	x (%)	x (%)	x (%)	x (%)	x (%)	x (%)
	COR-	n (%)	x (%)	x (%)	x (%)	x (%)	x (%)	x (%)
	COR indeterminate	n (%)	x (%)	x (%)	x (%)	x (%)	x (%)	x (%)
	COR missing	n (%)	x (%)	x (%)	x (%)	x (%)	x (%)	x (%)
**End of study**	All scheduled visits attended	n (%)	x (%)	x (%)	x (%)	x (%)	x (%)	x (%)
	Missed visits but no early termination	n (%)	x (%)	x (%)	x (%)	x (%)	x (%)	x (%)
	**Total early discontinuation / termination**	n (%)	x (%)	x (%)	x (%)	x (%)	x (%)	x (%)
	Prevalent TB case (diagnosed in study)	n (%)	x (%)	x (%)	x (%)	x (%)	x (%)	x (%)
	Incident TB case (diagnosed in study)	n (%)	x (%)	x (%)	x (%)	x (%)	x (%)	x (%)
	TB case (diagnosed and treated for TB externally)	n (%)	x (%)	x (%)	x (%)	x (%)	x (%)	x (%)
	Consent withdrawal	n (%)	x (%)	x (%)	x (%)	x (%)	x (%)	x (%)
	Withdrawal by investigator	n (%)	x (%)	x (%)	x (%)	x (%)	x (%)	x (%)
	LTFU	n (%)	x (%)	x (%)	x (%)	x (%)	x (%)	x (%)
	Death	n (%)	x (%)	x (%)	x (%)	x (%)	x (%)	x (%)
	Pregnancy	n (%)	x (%)	x (%)	x (%)	x (%)	x (%)	x (%)
	Protocol deviation/violation	n (%)	x (%)	x (%)	x (%)	x (%)	x (%)	x (%)
	Other	n (%)	x (%)	x (%)	x (%)	x (%)	x (%)	x (%)
	Missing end of study reason	n (%)	x (%)	x (%)	x (%)	x (%)	x (%)	x (%)

Note: Percentages for Category column are computed using total screened or total enrolled as denominator. Percentages for Subcategory column are computed using total in the particular Category column as denominator.

4.2 ENDPOINT ACCRUAL

		AlKlerks		AlRusten		CAPRISA		SATVI		SUN		Total	
		COR+	COR-	COR+	COR-	COR+	COR-	COR+	COR-	COR+	COR-	COR+	COR-
**Total subjects enrolled**	N	x	x	x	x	x	x	x	x	x	x	x	x
**Prevalent TB: Subjects diagnosed with TB at enrolment/visit 1***	n (%)	x (%)	x (%)	x (%)	x (%)	x (%)	x (%)	x (%)	x (%)	x (%)	x (%)	x (%)	x (%)
**1. Primary endpoint: based on two-sample detection**	n (%)	x (%)	x (%)	x (%)	x (%)	x (%)	x (%)	x (%)	x (%)	x (%)	x (%)	x (%)	x (%)
Positive Xpert MTB/RIF	n (%)	x (%)	x (%)	x (%)	x (%)	x (%)	x (%)	x (%)	x (%)	x (%)	x (%)	x (%)	x (%)
Positive MGIT culture	n (%)	x (%)	x (%)	x (%)	x (%)	x (%)	x (%)	x (%)	x (%)	x (%)	x (%)	x (%)	x (%)
Positive Xpert MTB/RIF & MGIT culture	n (%)	x (%)	x (%)	x (%)	x (%)	x (%)	x (%)	x (%)	x (%)	x (%)	x (%)	x (%)	x (%)
**2. Secondary endpoint: based on one-sample detection**	n (%)	x (%)	x (%)	x (%)	x (%)	x (%)	x (%)	x (%)	x (%)	x (%)	x (%)	x (%)	x (%)
Positive Xpert MTB/RIF	n (%)	x (%)	x (%)	x (%)	x (%)	x (%)	x (%)	x (%)	x (%)	x (%)	x (%)	x (%)	x (%)
Positive MGIT culture	n (%)	x (%)	x (%)	x (%)	x (%)	x (%)	x (%)	x (%)	x (%)	x (%)	x (%)	x (%)	x (%)
Positive Xpert MTB/RIF & MGIT culture	n (%)	x (%)	x (%)	x (%)	x (%)	x (%)	x (%)	x (%)	x (%)	x (%)	x (%)	x (%)	x (%)
**Incident TB: Subjects diagnosed with TB at Visits 2-8***	n (%)	x (%)	x (%)	x (%)	x (%)	x (%)	x (%)	x (%)	x (%)	x (%)	x (%)	x (%)	x (%)
**1. Primary endpoint: based on two-sample detection**	n (%)	x (%)	x (%)	x (%)	x (%)	x (%)	x (%)	x (%)	x (%)	x (%)	x (%)	x (%)	x (%)
Positive Xpert MTB/RIF	n (%)	x (%)	x (%)	x (%)	x (%)	x (%)	x (%)	x (%)	x (%)	x (%)	x (%)	x (%)	x (%)
Positive MGIT culture	n (%)	x (%)	x (%)	x (%)	x (%)	x (%)	x (%)	x (%)	x (%)	x (%)	x (%)	x (%)	x (%)
Positive Xpert MTB/RIF & MGIT culture	n (%)	x (%)	x (%)	x (%)	x (%)	x (%)	x (%)	x (%)	x (%)	x (%)	x (%)	x (%)	x (%)
**2. Secondary endpoint: based on one-sample detection**	n (%)	x (%)	x (%)	x (%)	x (%)	x (%)	x (%)	x (%)	x (%)	x (%)	x (%)	x (%)	x (%)
Positive Xpert MTB/RIF	n (%)	x (%)	x (%)	x (%)	x (%)	x (%)	x (%)	x (%)	x (%)	x (%)	x (%)	x (%)	x (%)
Positive MGIT culture	n (%)	x (%)	x (%)	x (%)	x (%)	x (%)	x (%)	x (%)	x (%)	x (%)	x (%)	x (%)	x (%)
Positive Xpert MTB/RIF & MGIT culture	n (%)	x (%)	x (%)	x (%)	x (%)	x (%)	x (%)	x (%)	x (%)	x (%)	x (%)	x (%)	x (%)
**Enrolled participants with TB diagnosed on clinical grounds, or not having a one- or two-sputum sample positive endpoint (excluded from diagnostic analysis)**	n (%)	x (%)	x (%)	x (%)	x (%)	x (%)	x (%)	x (%)	x (%)	x (%)	x (%)	x (%)	x (%)

Note: * Percentages in this group are computed using total subjects enrolled (Row 1) as denominator. All subjects with one-sample detection had more than one specimen tested.

4.3 ENROLMENT AND ENDPOINT ACCRUAL BY COR, ART AND IPT STATUS

		ART+			ART-			Total		
		IPT+	IPT-	Total	IPT+	IPT-	Total	IPT+	IPT-	Total
**Total subjects enrolled**	N	x	x	x	x	x	x	x	x	x
COR+	n (%)	x (%)	x (%)	x (%)	x (%)	x (%)	x (%)	x (%)	x (%)	x (%)
COR-	n (%)	x (%)	x (%)	x (%)	x (%)	x (%)	x (%)	x (%)	x (%)	x (%)
COR indeterminate*	n (%)	x (%)	x (%)	x (%)	x (%)	x (%)	x (%)	x (%)	x (%)	x (%)
**Subjects diagnosed with prevalent TB at enrolment/visit 1**	n (%)	x (%)	x (%)	x (%)	x (%)	x (%)	x (%)	x (%)	x (%)	x (%)
Based on two-sample detection	n (%)	x (%)	x (%)	x (%)	x (%)	x (%)	x (%)	x (%)	x (%)	x (%)
Based on one-sample detection	n (%)	x (%)	x (%)	x (%)	x (%)	x (%)	x (%)	x (%)	x (%)	x (%)
**Subjects diagnosed with incident TB during follow-up**	n (%)	x (%)	x (%)	x (%)	x (%)	x (%)	x (%)	x (%)	x (%)	x (%)
Based on two-sample detection	n (%)	x (%)	x (%)	x (%)	x (%)	x (%)	x (%)	x (%)	x (%)	x (%)
Based on one-sample detection	n (%)	x (%)	x (%)	x (%)	x (%)	x (%)	x (%)	x (%)	x (%)	x (%)
**COR+**	n (%)	x (%)	x (%)	x (%)	x (%)	x (%)	x (%)	x (%)	x (%)	x (%)
Subjects diagnosed with prevalent TB at enrolment/visit 1	n (%)	x (%)	x (%)	x (%)	x (%)	x (%)	x (%)	x (%)	x (%)	x (%)
Based on two-sample detection	n (%)	x (%)	x (%)	x (%)	x (%)	x (%)	x (%)	x (%)	x (%)	x (%)
Based on one-sample detection	n (%)	x (%)	x (%)	x (%)	x (%)	x (%)	x (%)	x (%)	x (%)	x (%)
Subjects diagnosed with incident TB during follow-up	n (%)	x (%)	x (%)	x (%)	x (%)	x (%)	x (%)	x (%)	x (%)	x (%)
Based on two-sample detection	n (%)	x (%)	x (%)	x (%)	x (%)	x (%)	x (%)	x (%)	x (%)	x (%)
Based on one-sample detection	n (%)	x (%)	x (%)	x (%)	x (%)	x (%)	x (%)	x (%)	x (%)	x (%)
**COR-**	n (%)	x (%)	x (%)	x (%)	x (%)	x (%)	x (%)	x (%)	x (%)	x (%)
Subjects diagnosed with prevalent TB at enrolment/visit 1	n (%)	x (%)	x (%)	x (%)	x (%)	x (%)	x (%)	x (%)	x (%)	x (%)
Based on two-sample detection	n (%)	x (%)	x (%)	x (%)	x (%)	x (%)	x (%)	x (%)	x (%)	x (%)
Based on one-sample detection	n (%)	x (%)	x (%)	x (%)	x (%)	x (%)	x (%)	x (%)	x (%)	x (%)
Subjects diagnosed with incident TB during follow-up	n (%)	x (%)	x (%)	x (%)	x (%)	x (%)	x (%)	x (%)	x (%)	x (%)
Based on two-sample detection	n (%)	x (%)	x (%)	x (%)	x (%)	x (%)	x (%)	x (%)	x (%)	x (%)
Based on one-sample detection	n (%)	x (%)	x (%)	x (%)	x (%)	x (%)	x (%)	x (%)	x (%)	x (%)
**COR indeterminate**	n (%)	x (%)	x (%)	x (%)	x (%)	x (%)	x (%)	x (%)	x (%)	x (%)
Subjects diagnosed with prevalent TB at enrolment/visit 1	n (%)	x (%)	x (%)	x (%)	x (%)	x (%)	x (%)	x (%)	x (%)	x (%)
Based on two-sample detection	n (%)	x (%)	x (%)	x (%)	x (%)	x (%)	x (%)	x (%)	x (%)	x (%)
Based on one-sample detection	n (%)	x (%)	x (%)	x (%)	x (%)	x (%)	x (%)	x (%)	x (%)	x (%)
Subjects diagnosed with incident TB during follow-up	n (%)	x (%)	x (%)	x (%)	x (%)	x (%)	x (%)	x (%)	x (%)	x (%)
Based on two-sample detection	n (%)	x (%)	x (%)	x (%)	x (%)	x (%)	x (%)	x (%)	x (%)	x (%)
Based on one-sample detection	n (%)	x (%)	x (%)	x (%)	x (%)	x (%)	x (%)	x (%)	x (%)	x (%)

Note: Percentages are computed using total number in enrolled cohort as denominator.

4.4 ENROLLED PARTICIPANT DEMOGRAPHICS AND CLINICAL CHARACTERISTICS, BY SITE

Variable	Levels		Total	AlKlerks	AlRusten	CAPRISA	SATVI	SUN	p value
Total enrolled		N	x	x	x	x	x	x	
**Demographics**									
Gender	Female	n (%)	x (%)	x (%)	x (%)	x (%)	x (%)	x (%)	p
	Male	n (%)	x (%)	x (%)	x (%)	x (%)	x (%)	x (%)	
Age		median (IQR)	x (x–x)	x (x–x)	x (x–x)	x (x–x)	x (x–x)	x (x–x)	p
Ethnicity	Black	n (%)	x (%)	x (%)	x (%)	x (%)	x (%)	x (%)	p
	Mixed Race	n (%)	x (%)	x (%)	x (%)	x (%)	x (%)	x (%)	
Highest education level	Primary School or Lower	n (%)	x (%)	x (%)	x (%)	x (%)	x (%)	x (%)	p
	Secondary School	n (%)	x (%)	x (%)	x (%)	x (%)	x (%)	x (%)	
	Tertiary	n (%)	x (%)	x (%)	x (%)	x (%)	x (%)	x (%)	
Employment status	Employed	n (%)	x (%)	x (%)	x (%)	x (%)	x (%)	x (%)	p
	Unemployed	n (%)	x (%)	x (%)	x (%)	x (%)	x (%)	x (%)	
Occupants per household		median (IQR)	x (x–x)	x (x–x)	x (x–x)	x (x–x)	x (x–x)	x (x–x)	p
Occupants per room		median (IQR)	x (x–x)	x (x–x)	x (x–x)	x (x–x)	x (x–x)	x (x–x)	p
Smoking history	Never	n (%)	x (%)	x (%)	x (%)	x (%)	x (%)	x (%)	p
	Current	n (%)	x (%)	x (%)	x (%)	x (%)	x (%)	x (%)	p
	Past	n (%)	x (%)	x (%)	x (%)	x (%)	x (%)	x (%)	p
History of prior TB	No	n (%)	x (%)	x (%)	x (%)	x (%)	x (%)	x (%)	p
	Yes	n (%)	x (%)	x (%)	x (%)	x (%)	x (%)	x (%)	p
TB household contacts	No	n (%)	x (%)	x (%)	x (%)	x (%)	x (%)	x (%)	p
	Yes	n (%)	x (%)	x (%)	x (%)	x (%)	x (%)	x (%)	p
Season of enrolment	Summer	n (%)	x (%)	x (%)	x (%)	x (%)	x (%)	x (%)	p
	Autumn	n (%)	x (%)	x (%)	x (%)	x (%)	x (%)	x (%)	
	Winter	n (%)	x (%)	x (%)	x (%)	x (%)	x (%)	x (%)	
	Spring	n (%)	x (%)	x (%)	x (%)	x (%)	x (%)	x (%)	

**Clinical variables**								
Weight (kg), baseline		median (IQR)	x (x–x)	x (x–x)	x (x–x)	x (x–x)	x (x–x)	p
	Missing data	n (%)	x (%)	x (%)	x (%)	x (%)	x (%)	p
Weight (kg), month 3		median (IQR)	x (x–x)	x (x–x)	x (x–x)	x (x–x)	x (x–x)	p
	Missing data	n (%)	x (%)	x (%)	x (%)	x (%)	x (%)	p
Body-mass index, baseline		median (IQR)	x (x–x)	x (x–x)	x (x–x)	x (x–x)	x (x–x)	p
	Missing data	n (%)	x (%)	x (%)	x (%)	x (%)	x (%)	p
	<18.5	n (%)	x (%)	x (%)	x (%)	x (%)	x (%)	p
	18.5-24.9	n (%)	x (%)	x (%)	x (%)	x (%)	x (%)	
	>25	n (%)	x (%)	x (%)	x (%)	x (%)	x (%)	
Weight change: M3-D0		median (IQR)	x (x–x)	x (x–x)	x (x–x)	x (x–x)	x (x–x)	p
Weight change: EOS-D0		median (IQR)	x (x–x)	x (x–x)	x (x–x)	x (x–x)	x (x–x)	p
Antiretroviral therapy (ART) at baseline	Started After Enrolment	n (%)	x (%)	x (%)	x (%)	x (%)	x (%)	p
	<6 Months	n (%)	x (%)	x (%)	x (%)	x (%)	x (%)	
	6-12 Months	n (%)	x (%)	x (%)	x (%)	x (%)	x (%)	
	>12 Months	n (%)	x (%)	x (%)	x (%)	x (%)	x (%)	
	No ART Recorded	n (%)	x (%)	x (%)	x (%)	x (%)	x (%)	
Isoniazid Preventive Therapy (IPT) at baseline	Started After Enrolment	n (%)	x (%)	x (%)	x (%)	x (%)	x (%)	p
	<6 Months	n (%)	x (%)	x (%)	x (%)	x (%)	x (%)	
	6-12 Months	n (%)	x (%)	x (%)	x (%)	x (%)	x (%)	
	>12 Months	n (%)	x (%)	x (%)	x (%)	x (%)	x (%)	
	No IPT Recorded	n (%)	x (%)	x (%)	x (%)	x (%)	x (%)	
**Laboratory results**								
QuantiFERON, baseline		median (IQR)	x (x–x)	x (x–x)	x (x–x)	x (x–x)	x (x–x)	p
	QuantiFERON not done/missing	n (%)	x (%)	x (%)	x (%)	x (%)	x (%)	p
	QuantiFERON Indeterminate	n (%)	x (%)	x (%)	x (%)	x (%)	x (%)	p
	Negative (<0.35)	n (%)	x (%)	x (%)	x (%)	x (%)	x (%)	p
	Low positive (0.35 to<1.0)	n (%)	x (%)	x (%)	x (%)	x (%)	x (%)	
	Medium positive (1.0 to<4.0)	n (%)	x (%)	x (%)	x (%)	x (%)	x (%)	
	High positive (≥4.0)	n (%)	x (%)	x (%)	x (%)	x (%)	x (%)	

QuantiFERON, baseline (binary)	Negative (<0.35)	n (%)	x (%)	x (%)	x (%)	x (%)	x (%)	p
	Positive (≥0.35)	n (%)	n (%)	n (%)	n (%)	n (%)	n (%)	
CD4 cell count, baseline		median (IQR)	x (x–x)	x (x–x)	x (x–x)	x (x–x)	x (x–x)	p
	CD4 cell count not done	n (%)	x (%)	x (%)	x (%)	x (%)	x (%)	p
	<100	n (%)	x (%)	x (%)	x (%)	x (%)	x (%)	p
	100-349	n (%)	x (%)	x (%)	x (%)	x (%)	x (%)	
	350-499	n (%)	x (%)	x (%)	x (%)	x (%)	x (%)	
	≥500	n (%)	x (%)	x (%)	x (%)	x (%)	x (%)	
CD4 cell count, baseline (binary)	<500	n (%)	x (%)	x (%)	x (%)	x (%)	x (%)	p
	≥500	n (%)	x (%)	x (%)	x (%)	x (%)	x (%)	
CD4 cell count, month 3		median (IQR)	x (x–x)	x (x–x)	x (x–x)	x (x–x)	x (x–x)	p
	CD4 not done/missing	n (%)	x (%)	x (%)	x (%)	x (%)	x (%)	p
	<100	n (%)	x (%)	x (%)	x (%)	x (%)	x (%)	p
	100-349	n (%)	x (%)	x (%)	x (%)	x (%)	x (%)	
	350-499	n (%)	x (%)	x (%)	x (%)	x (%)	x (%)	
	≥500	n (%)	x (%)	x (%)	x (%)	x (%)	x (%)	
CD4 cell count, month 3 (binary)	<500	n (%)	x (%)	x (%)	x (%)	x (%)	x (%)	p
	≥500	n (%)	x (%)	x (%)	x (%)	x (%)	x (%)	
Viral load, baseline	<100	n (%)	x (%)	x (%)	x (%)	x (%)	x (%)	p
	100-999	n (%)	x (%)	x (%)	x (%)	x (%)	x (%)	
	≥1000	n (%)	x (%)	x (%)	x (%)	x (%)	x (%)	
	Viral load not done/missing	n (%)	x (%)	x (%)	x (%)	x (%)	x (%)	p
Viral load, month 3	<100	n (%)	x (%)	x (%)	x (%)	x (%)	x (%)	p
	100-999	n (%)	x (%)	x (%)	x (%)	x (%)	x (%)	
	≥1000	n (%)	x (%)	x (%)	x (%)	x (%)	x (%)	
	Viral load not done/missing	n (%)	x (%)	x (%)	x (%)	x (%)	x (%)	p
COR score, baseline		median (IQR)	x (x–x)	x (x–x)	x (x–x)	x (x–x)	x (x–x)	p
	Indeterminate	n (%)	x (%)	x (%)	x (%)	x (%)	x (%)	p
	Not done/missing	n (%)	x (%)	x (%)	x (%)	x (%)	x (%)	p
COR score, month 3		median (IQR)	x (x–x)	x (x–x)	x (x–x)	x (x–x)	x (x–x)	p
	Indeterminate	n (%)	x (%)	x (%)	x (%)	x (%)	x (%)	p

	Not done/missing	n (%)	x (%)	x (%)	x (%)	x (%)	x (%)	p
Urinary LAM, baseline	Negative	n (%)	x (%)	x (%)	x (%)	x (%)	x (%)	p
	Positive	n (%)	x (%)	x (%)	x (%)	x (%)	x (%)	
	Not done/missing	n (%)	x (%)	x (%)	x (%)	x (%)	x (%)	p
Urinary LAM, month 3	Negative	n (%)	x (%)	x (%)	x (%)	x (%)	x (%)	p
	Positive	n (%)	x (%)	x (%)	x (%)	x (%)	x (%)	
	Not done/missing	n (%)	x (%)	x (%)	x (%)	x (%)	x (%)	p
**TB Symptoms**								
Cough > 2 Weeks	No	n (%)	x (%)	x (%)	x (%)	x (%)	x (%)	p
	Yes	n (%)	x (%)	x (%)	x (%)	x (%)	x (%)	
Haemoptysis in past 2 weeks	No	n (%)	x (%)	x (%)	x (%)	x (%)	x (%)	p
	Yes	n (%)	x (%)	x (%)	x (%)	x (%)	x (%)	
Weight loss > 2 weeks	No	n (%)	x (%)	x (%)	x (%)	x (%)	x (%)	p
	Yes	n (%)	x (%)	x (%)	x (%)	x (%)	x (%)	
Fever > 2 weeks	No	n (%)	x (%)	x (%)	x (%)	x (%)	x (%)	p
	Yes	n (%)	x (%)	x (%)	x (%)	x (%)	x (%)	
Night sweats > 2 weeks	No	n (%)	x (%)	x (%)	x (%)	x (%)	x (%)	p
	Yes	n (%)	x (%)	x (%)	x (%)	x (%)	x (%)	
Chest pain > 2 weeks	No	n (%)	x (%)	x (%)	x (%)	x (%)	x (%)	p
	Yes	n (%)	x (%)	x (%)	x (%)	x (%)	x (%)	
Flu-like symptoms in past 2 weeks	No	n (%)	x (%)	x (%)	x (%)	x (%)	x (%)	p
	Yes	n (%)	x (%)	x (%)	x (%)	x (%)	x (%)	
	Not recorded	n (%)	x (%)	x (%)	x (%)	x (%)	x (%)	p
WHO symptom screen	Negative	n (%)	x (%)	x (%)	x (%)	x (%)	x (%)	p
	Positive	n (%)	x (%)	x (%)	x (%)	x (%)	x (%)	
Number of TB symptoms	0	n (%)	x (%)	x (%)	x (%)	x (%)	x (%)	p
	1	n (%)	x (%)	x (%)	x (%)	x (%)	x (%)	
	2	n (%)	x (%)	x (%)	x (%)	x (%)	x (%)	
	3	n (%)	x (%)	x (%)	x (%)	x (%)	x (%)	
	4	n (%)	x (%)	x (%)	x (%)	x (%)	x (%)	

4.5 ENROLLED PARTICIPANT DEMOGRAPHICS AND CLINICAL CHARACTERISTICS BY CASE STATUS WITH CRUDE ODDS RATIO

**Variable**	**Levels**		**Healthy**	**Cumulative TB cases**	**OR (95% CI)**	**p value**	**Prevalent TB cases**	**OR (95% CI)**	**p value**	**Incident TB cases**	**OR (95% CI)**	**p value**
Total enrolled		N	x	x			x			x		
**Demographics**												
Gender	Female	n (%)	x (%)	x (%)	1		x (%)	1		x (%)	1	
	Male	n (%)	x (%)	x (%)	x (x–x)	p	x (%)	x (x–x)	p	x (%)	x (x–x)	p
Age		median (IQR)	x (x–x)	x (x–x)	x (x–x) per 10 years	p	x (x–x)	x (x–x) per 10 years	p	x (x–x)	x (x–x) per 10 years	p
Ethnicity	Black	n (%)	x (%)	x (%)	1		x (%)	1		x (%)	1	
	Mixed Race	n (%)	x (%)	x (%)	x (x–x)	p	x (%)	x (x–x)	p	x (%)	x (x–x)	p
City/Site	Worcester, Western Cape	n (%)	x (%)	x (%)	1		x (%)	1		x (%)	1	
	Durban, KwaZulu Natal	n (%)	x (%)	x (%)	x (x–x)	p	x (%)	x (x–x)	p	x (%)	x (x–x)	p
	Klerksdorp, North West	n (%)	x (%)	x (%)	x (x–x)	p	x (%)	x (x–x)	p	x (%)	x (x–x)	p
	Rustenburg, North West	n (%)	x (%)	x (%)	x (x–x)	p	x (%)	x (x–x)	p	x (%)	x (x–x)	p
	Stellenbosch, Western Cape	n (%)	x (%)	x (%)	x (x–x)	p	x (%)	x (x–x)	p	x (%)	x (x–x)	p
Highest education level	Primary School or Lower	n (%)	x (%)	x (%)	1		x (%)	1		x (%)	1	
	Secondary School	n (%)	x (%)	x (%)	x (x–x)	p	x (%)	x (x–x)	p	x (%)	x (x–x)	p
	Tertiary	n (%)	x (%)	x (%)	x (x–x)	p	x (%)	x (x–x)	p	x (%)	x (x–x)	p
Employment status	Employed	n (%)	x (%)	x (%)	1		x (%)	1		x (%)	1	
	Unemployed	n (%)	x (%)	x (%)	x (x–x)	p	x (%)	x (x–x)	p	x (%)	x (x–x)	p
Occupants per household		median (IQR)	x (x–x)	x (x–x)	x (x–x) per occupant	p	x (x–x)	x (x–x) per occupant	p	x (x–x)	x (x–x) per occupant	p
Occupants per room		median (IQR)	x (x–x)	x (x–x)	x (x–x) per occupant	p	x (x–x)	x (x–x) per occupant	p	x (x–x)	x (x–x) per occupant	p
Smoking history	Never	n (%)	x (%)	x (%)	1		x (%)	1		x (%)	1	
	Current	n (%)	x (%)	x (%)	x (x–x)	p	x (%)	x (x–x)	p	x (%)	x (x–x)	p
	Past	n (%)	x (%)	x (%)	x (x–x)	p	x (%)	x (x–x)	p	x (%)	x (x–x)	p
History of prior TB	No	n (%)	x (%)	x (%)	1		x (%)	1		x (%)	1	
	Yes	n (%)	x (%)	x (%)	x (x–x)	p	x (%)	x (x–x)	p	x (%)	x (x–x)	p
TB household contacts	No	n (%)	x (%)	x (%)	1		x (%)	1		x (%)	1	

	Yes	n (%)	x (%)	x (%)	x (x–x)	p	x (%)	x (x–x)	p	x (%)	x (x–x)	p
Season of enrolment	Summer	n (%)	x (%)	x (%)	1		x (%)	1		x (%)	1	
	Autumn	n (%)	x (%)	x (%)	x (x–x)	p	x (%)	x (x–x)	p	x (%)	x (x–x)	p
	Winter	n (%)	x (%)	x (%)	x (x–x)	p	x (%)	x (x–x)	p	x (%)	x (x–x)	p
	Spring	n (%)	x (%)	x (%)	x (x–x)	p	x (%)	x (x–x)	p	x (%)	x (x–x)	p
**Clinical variables**												
Weight (kg), baseline		median (IQR)	x (x–x)	x (x–x)	x (x–x) per 10kg	p	x (x–x)	x (x–x) per 10kg	p	x (x–x)	x (x–x) per 10kg	p
	Missing data	n (%)	x (%)	x (%)			x (%)			x (%)		
Weight (kg), month 3		median (IQR)	x (x–x)	x (x–x)	x (x–x) per 10kg	p	x (x–x)	x (x–x) per 10kg	p	x (x–x)	x (x–x) per 10kg	p
	Missing data	n (%)	x (%)	x (%)			x (%)			n (%)		
Body-mass index, baseline		median (IQR)	x (x–x)	x (x–x)	x (x–x) per unit	p	x (x–x)	x (x–x) per unit	p	x (x–x)	x (x–x) per unit	p
	Missing data	n (%)	x (%)	x (%)			x (%)			x (%)		
	<18.5	n (%)	x (%)	x (%)	1		x (%)	1		x (%)	1	
	18.5-24.9	n (%)	x (%)	x (%)	x (x–x)	p	x (%)	x (x–x)	p	x (%)	x (x–x)	p
	>25	n (%)	x (%)	x (%)	x (x–x)	p	x (%)	x (x–x)	p	x (%)	x (x–x)	p
Weight change: M3-D0		median (IQR)	x (x–x)	x (x–x)	x (x–x) per kg	p	x (x–x)	x (x–x) per kg	p	x (x–x)	x (x–x) per kg	p
Weight change: EOS-D0		median (IQR)	x (x–x)	x (x–x)	x (x–x) per kg	p	x (x–x)	x (x–x) per kg	p	x (x–x)	x (x–x) per kg	p
Antiretroviral therapy (ART) at baseline	Started After Enrolment	n (%)	x (%)	x (%)	1		x (%)	1		x (%)	1	
	<6 Months	n (%)	x (%)	x (%)	x (x–x)	p	x (%)	x (x–x)	p	x (%)	x (x–x)	p
	6-12 Months	n (%)	x (%)	x (%)	x (x–x)	p	x (%)	x (x–x)	p	x (%)	x (x–x)	p
	>12 Months	n (%)	x (%)	x (%)	x (x–x)	p	x (%)	x (x–x)	p	x (%)	x (x–x)	p
	No ART Recorded	n (%)	x (%)	x (%)	x (x–x)	p	x (%)	x (x–x)	p	x (%)	x (x–x)	p
Isoniazid Preventive Therapy (IPT) at baseline	Started After Enrolment	n (%)	x (%)	x (%)	1		x (%)	1		x (%)	1	
	<6 Months	n (%)	x (%)	x (%)	x (x–x)	p	x (%)	x (x–x)	p	x (%)	x (x–x)	p
	6-12 Months	n (%)	x (%)	x (%)	x (x–x)	p	x (%)	x (x–x)	p	x (%)	x (x–x)	p
	>12 Months	n (%)	x (%)	x (%)	x (x–x)	p	x (%)	x (x–x)	p	x (%)	x (x–x)	p
	No IPT Recorded	n (%)	x (%)	x (%)	x (x–x)	p	x (%)	x (x–x)	p	x (%)	x (x–x)	p

**Laboratory results**												
QuantiFERON, baseline		median (IQR)	x (x–x)	x (x–x)	x (x–x) per 50 cells	p	x (x–x)	x (x–x) per 50 cells	p	x (x–x)	x (x–x) per 50 cells	p
	QuantiFERON done/missing	n (%)	x (%)	x (%)			x (%)			x (%)		
	QuantiFERON indeterminate	n (%)	x (%)	x (%)			x (%)			x (%)		
	Negative (<0.35)	n (%)	x (%)	x (%)	1		x (%)	1		x (%)	1	
	Low positive (0.35 to<1.0)	n (%)	x (%)	x (%)	x (x–x)	p	x (%)	x (x–x)	p	x (%)	x (x–x)	p
	Medium positive (1.0 to<4.0)	n (%)	x (%)	x (%)	x (x–x)	p	x (%)	x (x–x)	p	x (%)	x (x–x)	p
	High positive (≥4.0)	n (%)	x (%)	x (%)	x (x–x)	p	x (%)	x (x–x)	p	x (%)	x (x–x)	p
QuantiFERON, baseline (binary)	Negative (<0.35)	n (%)	x (%)	x (%)	1		x (%)	1		x (%)	1	
	Positive (≥0.35)	n (%)	n (%)	n (%)	x (x–x)	p	n (%)	x (x–x)	p	n (%)	x (x–x)	p
CD4 cell count, baseline		median (IQR)	x (x–x)	x (x–x)	x (x–x) per 50 cells	p	x (x–x)	x (x–x) per 50 cells	p	x (x–x)	x (x–x) per 50 cells	p
	CD4 not done/missing	n (%)	x (%)	x (%)			x (%)			x (%)		
	<100	n (%)	x (%)	x (%)	1		x (%)	1		x (%)	1	
	100-349	n (%)	x (%)	x (%)	x (x–x)	p	x (%)	x (x–x)	p	x (%)	x (x–x)	p
	350-499	n (%)	x (%)	x (%)	x (x–x)	p	x (%)	x (x–x)	p	x (%)	x (x–x)	p
	≥500	n (%)	x (%)	x (%)	x (x–x)	p	x (%)	x (x–x)	p	x (%)	x (x–x)	p
CD4 cell count, baseline (binary)	<500	n (%)	x (%)	x (%)	1		x (%)	1		x (%)	1	
	≥500	n (%)	x (%)	x (%)	x (x–x)	p	x (%)	x (x–x)	p	x (%)	x (x–x)	p
CD4 cell count, month 3		median (IQR)	x (x–x)	x (x–x)	x (x–x) per 50 cells	p	x (x–x)	x (x–x) per 50 cells	p	x (x–x)	x (x–x) per 50 cells	p
	CD4 not done/missing	n (%)	x (%)	x (%)			x (%)			x (%)		
	<100	n (%)	x (%)	x (%)	1		x (%)	1		x (%)	1	
	100-349	n (%)	x (%)	x (%)	x (x–x)	p	x (%)	x (x–x)	p	x (%)	x (x–x)	p
	350-499	n (%)	x (%)	x (%)	x (x–x)	p	x (%)	x (x–x)	p	x (%)	x (x–x)	p
	≥500	n (%)	x (%)	x (%)	x (x–x)	p	x (%)	x (x–x)	p	x (%)	x (x–x)	p
CD4 cell count, month 3 (binary)	<500	n (%)	x (%)	x (%)	1		x (%)	1		x (%)	1	
	≥500	n (%)	x (%)	x (%)	x (x–x)	p	x (%)	x (x–x)	p	x (%)	x (x–x)	p
Viral load, baseline	<100	n (%)	x (%)	x (%)	1		x (%)	1		x (%)	1	
	100-999	n (%)	x (%)	x (%)	x (x–x)	p	x (%)	x (x–x)	p	x (%)	x (x–x)	p
	≥1000	n (%)	x (%)	x (%)	x (x–x)	p	x (%)	x (x–x)	p	x (%)	x (x–x)	p

	Viral load not done/missing	n (%)	x (%)	x (%)			x (%)			x (%)		
Viral load, month 3	<100	n (%)	x (%)	x (%)	1		x (%)	1		x (%)	1	
	100-999	n (%)	x (%)	x (%)	x (x–x)	p	x (%)	x (x–x)	p	x (%)	x (x–x)	p
	≥1000	n (%)	x (%)	x (%)	x (x–x)	p	x (%)	x (x–x)	p	x (%)	x (x–x)	p
	Viral load not done/missing	n (%)	x (%)	x (%)			x (%)			x (%)		
COR score, baseline		median (IQR)	x (x–x)	x (x–x)	x (x–x) per 10%	p	x (x–x)	x (x–x) per 10%	p	x (x–x)	x (x–x) per 10%	p
	Indeterminate	n (%)	x (%)	x (%)			x (%)			x (%)		
	Not done/missing	n (%)	x (%)	x (%)			x (%)			x (%)		
COR score, month 3		median (IQR)	x (x–x)	x (x–x)	x (x–x) per 10%	p	x (x–x)	x (x–x) per 10%	p	x (x–x)	x (x–x) per 10%	p
	Indeterminate	n (%)	x (%)	x (%)			x (%)			x (%)		
	Not done/missing	n (%)	x (%)	x (%)			x (%)			x (%)		
Urinary LAM, baseline	Negative	n (%)	x (%)	x (%)	1		x (%)	1		x (%)	1	
	Positive	n (%)	x (%)	x (%)	x (x–x)	p	x (%)	x (x–x)	p	x (%)	x (x–x)	p
	Not done/missing	n (%)	x (%)	x (%)			x (%)			x (%)		
Urinary LAM, month 3	Negative	n (%)	x (%)	x (%)	1		x (%)	1		x (%)	1	
	Positive	n (%)	x (%)	x (%)	x (x–x)	p	x (%)	x (x–x)	p	x (%)	x (x–x)	p
	Not done/missing	n (%)	x (%)	x (%)			x (%)			x (%)		
**Symptom screening**												
Cough > 2 Weeks	No	n (%)	x (%)	x (%)	1		x (%)	1		x (%)	1	
	Yes	n (%)	x (%)	x (%)	x (x–x)	p	x (%)	x (x–x)	p	x (%)	x (x–x)	p
Haemoptysis in past 2 weeks	No	n (%)	x (%)	x (%)	1		x (%)	1		x (%)	1	
	Yes	n (%)	x (%)	x (%)	x (x–x)	p	x (%)	x (x–x)	p	x (%)	x (x–x)	p
Weight loss > 2 weeks	No	n (%)	x (%)	x (%)	1		x (%)	1		x (%)	1	
	Yes	n (%)	x (%)	x (%)	x (x–x)	p	x (%)	x (x–x)	p	x (%)	x (x–x)	p
Fever > 2 weeks	No	n (%)	x (%)	x (%)	1		x (%)	1		x (%)	1	
	Yes	n (%)	x (%)	x (%)	x (x–x)	p	x (%)	x (x–x)	p	x (%)	x (x–x)	p
Night sweats > 2 weeks	No	n (%)	x (%)	x (%)	1		x (%)	1		x (%)	1	
	Yes	n (%)	x (%)	x (%)	x (x–x)	p	x (%)	x (x–x)	p	x (%)	x (x–x)	p
Chest pain > 2 weeks	No	n (%)	x (%)	x (%)	1		x (%)	1		x (%)	1	
	Yes	n (%)	x (%)	x (%)	x (x–x)	p	x (%)	x (x–x)	p	x (%)	x (x–x)	p

Flu-like symptoms in past 2 weeks	No	n (%)	x (%)	x (%)	1		x (%)	1		x (%)	1	
	Yes	n (%)	x (%)	x (%)	x (x–x)	p	x (%)	x (x–x)	p	x (%)	x (x–x)	p
	Not recorded	n (%)	x (%)	x (%)			x (%)			x (%)		
WHO symptom screen	Negative	n (%)	x (%)	x (%)	1		x (%)	1		x (%)	1	
	Positive	n (%)	x (%)	x (%)	x (x–x)	p	x (%)	x (x–x)	p	x (%)	x (x–x)	p
Number of TB symptoms	0	n (%)	x (%)	x (%)	1		x (%)	1		x (%)	1	
	1	n (%)	x (%)	x (%)	x (x–x)	p	x (%)	x (x–x)	p	x (%)	x (x–x)	p
	2	n (%)	x (%)	x (%)	x (x–x)	p	x (%)	x (x–x)	p	x (%)	x (x–x)	p
	3	n (%)	x (%)	x (%)	x (x–x)	p	x (%)	x (x–x)	p	x (%)	x (x–x)	p
	4	n (%)	x (%)	x (%)	x (x–x)	p	x (%)	x (x–x)	p	x (%)	x (x–x)	p

OR = Odds ratio

4.6 PRIMARY AND SECONDARY OUTCOMES TABLES (BINARY ANALYSIS)

4.6.1 CUMULATIVE TEST PERFORMANCE FOR PRIMARY END-POINT (TWO-SAMPLE) PREVALENT AND INCIDENT TB CASES DURING FOLLOW-UP (PRIMARY AIM)

4.6.2 CUMULATIVE TEST PERFORMANCE FOR COMBINED PRIMARY AND SECONDARY END-POINT (ONE- OR TWO-SAMPLE) PREVALENT AND INCIDENT TB CASES DURING FOLLOW-UP (PRIMARY AIM)

4.6.3 SCREENING/TRIAGE TEST PERFORMANCE FOR PRIMARY END-POINT (TWO-SAMPLE) PREVALENT TB CASES AT ENROLMENT (SECONDARY AIM 1)

4.6.4 SCREENING/TRIAGE TEST PERFORMANCE FOR COMBINED PRIMARY AND SECONDARY END-POINT (ONE- OR TWO-SAMPLE) PREVALENT TB CASES AT ENROLMENT (SECONDARY AIM 1)

4.6.5 PREDICTIVE TEST PERFORMANCE FOR PRIMARY END-POINT (TWO-SAMPLE) INCIDENT TB CASES DURING FOLLOW-UP

4.6.6 PREDICTIVE TEST PERFORMANCE FOR COMBINED PRIMARY AND SECONDARY END-POINT (ONE- OR TWO-SAMPLE) INCIDENT TB CASES DURING FOLLOW-UP

	Missing or indeterminate result	TP	FN	TN	FP	AUC, % (95% CI)	RR or CIR, n (95% CI)	Sensitivity‡ % (95% CI)	Specificity‡ % (95% CI)	PPV, % (95% CI)	NPV, % (95% CI)	NNS, n (95% CI)
**Symptom-based screening***												
1 or more WHO symptoms positive	x	x	x	x	x		x (x–x)	x% (x–x)	x% (x–x)	x% (x–x)	x% (x–x)	x (x–x)
2 or more WHO symptoms positive	x	x	x	x	x		x (x–x)	x% (x–x)	x% (x–x)	x% (x–x)	x% (x–x)	x (x–x)
3 or more WHO symptoms positive	x	x	x	x	x		x (x–x)	x% (x–x)	x% (x–x)	x% (x–x)	x% (x–x)	x (x–x)
4 or more WHO symptoms positive	x	x	x	x	x		x (x–x)	x% (x–x)	x% (x–x)	x% (x–x)	x% (x–x)	x (x–x)
Cough for ≥2 weeks (with or without presence of other symptoms)	x	x	x	x	x		x (x–x)	x% (x–x)	x% (x–x)	x% (x–x)	x% (x–x)	x (x–x)
Cough for ≥2 weeks and at least one other symptom	x	x	x	x	x		x (x–x)	x% (x–x)	x% (x–x)	x% (x–x)	x% (x–x)	x (x–x)
Any symptom (including chest pain and flu-like symptoms)	x	x	x	x	x		x (x–x)	x% (x–x)	x% (x–x)	x% (x–x)	x% (x–x)	x (x–x)
**QuantiFERON-TB Gold In-tube assay (QFT)**												
Optimal QFT threshold (≥xx IU/ml) ‡	x	x	x	x	x	x% (x–x)	x (x–x)	x% (x–x)	x% (x–x)	x% (x–x)	x% (x–x)	x (x–x)
A-priori QFT threshold, as per manufacturer (≥0.35 IU/ml)	x	x	x	x	x		x (x–x)	x% (x–x)	x% (x–x)	x% (x–x)	x% (x–x)	x (x–x)
QFT threshold >0.7 IU/ml+	x	x	x	x	x		x (x–x)	x% (x–x)	x% (x–x)	x% (x–x)	x% (x–x)	x (x–x)
QFT <0.2 IU/ml NEGATIVE+ QFT 0.2–0.7 IU/ml INDETERMINATE+ QFT >0.7 IU/ml POSITIVE+	x	x	x	x	x		x (x–x)	x% (x–x)	x% (x–x)	x% (x–x)	x% (x–x)	x (x–x)
QFT threshold ≥1 IU/ml§	x	x	x	x	x		x (x–x)	x% (x–x)	x% (x–x)	x% (x–x)	x% (x–x)	x (x–x)
QFT threshold ≥4 IU/ml§	x	x	x	x	x		x (x–x)	x% (x–x)	x% (x–x)	x% (x–x)	x% (x–x)	x (x–x)
**Urine Alere LAM**	x	x	x	x	x		x (x–x)	x% (x–x)	x% (x–x)	x% (x–x)	x% (x–x)	x (x–x)
**COR (RISK11) †**												
Optimal COR threshold (≥xx) ‡	x	x	x	x	x	x% (x–x)	x (x–x)	x% (x–x)	x% (x–x)	x% (x–x)	x% (x–x)	x (x–x)
COR threshold ≥90	x	x	x	x	x		x (x–x)	x% (x–x)	x% (x–x)	x% (x–x)	x% (x–x)	x (x–x)
COR threshold ≥80	x	x	x	x	x		x (x–x)	x% (x–x)	x% (x–x)	x% (x–x)	x% (x–x)	x (x–x)
COR threshold ≥70	x	x	x	x	x		x (x–x)	x% (x–x)	x% (x–x)	x% (x–x)	x% (x–x)	x (x–x)
COR threshold ≥60	x	x	x	x	x		x (x–x)	x% (x–x)	x% (x–x)	x% (x–x)	x% (x–x)	x (x–x)
COR threshold ≥50	x	x	x	x	x		x (x–x)	x% (x–x)	x% (x–x)	x% (x–x)	x% (x–x)	x (x–x)

COR threshold ≥40	x	x	x	x	x		x (x–x)	x% (x–x)	x% (x–x)	x% (x–x)	x% (x–x)	x (x–x)
COR threshold ≥30	x	x	x	x	x		x (x–x)	x% (x–x)	x% (x–x)	x% (x–x)	x% (x–x)	x (x–x)
COR threshold ≥20	x	x	x	x	x		x (x–x)	x% (x–x)	x% (x–x)	x% (x–x)	x% (x–x)	x (x–x)
COR threshold ≥10	x	x	x	x	x		x (x–x)	x% (x–x)	x% (x–x)	x% (x–x)	x% (x–x)	x (x–x)
**COR (RISK11): indeterminate results included**												
Optimal COR threshold (≥xx) ‡	x	x	x	x	x	x% (x–x)	x (x–x)	x% (x–x)	x% (x–x)	x% (x–x)	x% (x–x)	x (x–x)
COR threshold ≥90	x	x	x	x	x		x (x–x)	x% (x–x)	x% (x–x)	x% (x–x)	x% (x–x)	x (x–x)
COR threshold ≥80	x	x	x	x	x		x (x–x)	x% (x–x)	x% (x–x)	x% (x–x)	x% (x–x)	x (x–x)
COR threshold ≥70	x	x	x	x	x		x (x–x)	x% (x–x)	x% (x–x)	x% (x–x)	x% (x–x)	x (x–x)
COR threshold ≥60	x	x	x	x	x		x (x–x)	x% (x–x)	x% (x–x)	x% (x–x)	x% (x–x)	x (x–x)
COR threshold ≥50	x	x	x	x	x		x (x–x)	x% (x–x)	x% (x–x)	x% (x–x)	x% (x–x)	x (x–x)
COR threshold ≥40	x	x	x	x	x		x (x–x)	x% (x–x)	x% (x–x)	x% (x–x)	x% (x–x)	x (x–x)
COR threshold ≥30	x	x	x	x	x		x (x–x)	x% (x–x)	x% (x–x)	x% (x–x)	x% (x–x)	x (x–x)
COR threshold ≥20	x	x	x	x	x		x (x–x)	x% (x–x)	x% (x–x)	x% (x–x)	x% (x–x)	x (x–x)
COR threshold ≥10	x	x	x	x	x		x (x–x)	x% (x–x)	x% (x–x)	x% (x–x)	x% (x–x)	x (x–x)

TP = True positive; FN = False negative; TN = True negative; FP = False positive; AUC = Area under the receiver operating characteristics (ROC) curve; CIR = Cumulative incidence ratio; RR = Relative risk; PPV = Positive predictive value; NPV = Negative predictive value; NNS = Number needed to screen to detect 1 case of incident TB; COR = Correlate of risk.

Note: Participants who were unable to produce satisfactory sputum samples at enrolment or at end-of-study visit were assumed to be sputum negative at those time-point.

* TB symptoms include loss of weight, persistent unexplained cough, fever, or night sweats for longer than two weeks; or any haemoptysis.

+ Nemes E, Rozot V, Geldenhuys H, Bilek N, Mabwe S, Abrahams D, et al. Optimization and Interpretation of Serial QuantiFERON Testing to Measure Acquisition of Mycobacterium tuberculosis Infection. American Journal of Respiratory and Critical Care Medicine. 2017;196(5):638-48. doi: 10.1164/rccm.201704-0817OC.

§ Winje BA, White R, Syre H, Skutlaberg DH, Oftung F, Mengshoel AT, et al. Stratification by interferon-gamma release assay level predicts risk of incident TB. Thorax. 2018. doi: 10.1136/thoraxjnl-2017-211147.

† Indeterminate results excluded.

‡ Threshold with specificity benchmarked at 70% for screening/triage and 75% for prediction of incident TB cases.

4.7 PERFORMANCE OF THE COR (RISK11) AND QFT FOR IDENTIFICATION OF TB DISEASE OVER A 15-MONTH PERIOD, STRATIFIED BY THE TIME INTERVAL TO DISEASE (TIME-DEPENDENT ANALYSIS)

4.7.1 CUMULATIVE TEST PERFORMANCE FOR PRIMARY END-POINT (TWO-SAMPLE) PREVALENT AND INCIDENT TB CASES DURING FOLLOW-UP (PRIMARY AIM)

4.7.2 CUMULATIVE TEST PERFORMANCE FOR COMBINED PRIMARY AND SECONDARY END-POINT (ONE- OR TWO-SAMPLE) PREVALENT AND INCIDENT TB CASES DURING FOLLOW-UP (PRIMARY AIM)

4.7.3 PREDICTIVE TEST PERFORMANCE FOR PRIMARY END-POINT (TWO-SAMPLE) INCIDENT TB CASES DURING FOLLOW-UP (SECONDARY AIMS 2 AND 3)

4.7.4 PREDICTIVE TEST PERFORMANCE FOR COMBINED PRIMARY AND SECONDARY END-POINT (ONE- OR TWO-SAMPLE) INCIDENT TB CASES DURING FOLLOW-UP (SECONDARY AIMS 2 AND 3)

Test and time interval to TB disease	Cuminc COR+, % (95% CI)	Cuminc COR-, % (95% CI)	CIR, n (95% CI)	p value	AUC, % (95% CI)	Sensitivity‡ % (95% CI)	Specificity‡ % (95% CI)	PPV, % (95% CI)	NPV, % (95% CI)	NNS, n (95% CI)
**QuantiFERON-TB Gold In-tube assay (QFT)** †										
0 to 3 months	x (x–x)	x (x–x)	x (x–x)	x	x% (x–x)	x% (x–x)	x% (x–x)	x% (x–x)	x% (x–x)	x (x–x)
0 to 6 months	x (x–x)	x (x–x)	x (x–x)	x	x% (x–x)	x% (x–x)	x% (x–x)	x% (x–x)	x% (x–x)	x (x–x)
0 to 12 months	x (x–x)	x (x–x)	x (x–x)	x	x% (x–x)	x% (x–x)	x% (x–x)	x% (x–x)	x% (x–x)	x (x–x)
0 to 15 months	x (x–x)	x (x–x)	x (x–x)	x	x% (x–x)	x% (x–x)	x% (x–x)	x% (x–x)	x% (x–x)	x (x–x)
**COR (RISK11)** †										
0 to 3 months	x (x–x)	x (x–x)	x (x–x)	x	x% (x–x)	x% (x–x)	x% (x–x)	x% (x–x)	x% (x–x)	x (x–x)
0 to 6 months	x (x–x)	x (x–x)	x (x–x)	x	x% (x–x)	x% (x–x)	x% (x–x)	x% (x–x)	x% (x–x)	x (x–x)
0 to 12 months	x (x–x)	x (x–x)	x (x–x)	x	x% (x–x)	x% (x–x)	x% (x–x)	x% (x–x)	x% (x–x)	x (x–x)
0 to 15 months	x (x–x)	x (x–x)	x (x–x)	x	x% (x–x)	x% (x–x)	x% (x–x)	x% (x–x)	x% (x–x)	x (x–x)

Cuminc = Cumulative incidence; CIR = Cumulative incidence ratio; AUC = Area under the receiver operating characteristics (ROC) curve; PPV = Positive predictive value; NPV = Negative predictive value; NNS = Number needed to screen to detect 1 case of incident TB; COR = Correlate of risk.

† Indeterminate results excluded.

‡ Threshold with specificity benchmarked at 75% for prediction of incident TB cases.

5 FIGURES

5.1 TYPES OF FIGURES

5.1.1 Test performance: ROC curves (sensitivity versus 100-specificity)

5.1.2 Test accuracy: sensitivity/specificity versus test score threshold plots (x-axis: score threshold, y-axis: sensitivity or specificity)

5.1.3 Test score distribution: violin/box-and-scatter plots (Prevalent and incident TB cases versus TB negative controls)

5.1.4 Time dependent analysis: AUC versus time. CIR versus time. Sensitivity, specificity, NPV and PPV (at different thresholds) versus time, over 15-month follow-up

5.2 ENDPOINTS FOR FIGURES

5.2.1 Cumulative test performance for primary endpoint (two-sample) prevalent and incident TB cases during follow-up (Primary aim)

5.2.2 Cumulative test performance for combined primary and secondary endpoint (one- or two-sample) prevalent and incident TB cases during follow-up (Primary aim)

5.2.3 Screening/triage test performance for primary endpoint (two-sample) prevalent TB cases at enrolment (Secondary aim 1)

5.2.4 Screening/triage test performance for combined primary and secondary endpoint (one- or two-sample) prevalent TB cases at enrolment (Secondary aim 1)

5.2.5 Predictive test performance for primary endpoint (two-sample) incident TB cases during follow-up (Secondary aim 2)

5.2.6 Predictive test performance for combined primary and secondary endpoint (one- or two-sample) incident TB cases during follow-up (Secondary aim 2)

5.3 TESTS

5.3.1 COR (RISK11)

5.3.2 QuantiFERON

6 APPENDIX

6.1 WHO DIAGNOSTIC, TRIAGE, AND PREDICTIVE TB TEST PERFORMANCE TARGET PRODUCT PROFILE

1. Rapid biomarker-based non-sputum-based test for detecting (diagnosing) PTB (World Health Organization, 2014)

	**Minimum diagnostic**	**Optimal diagnostic**
**Sensitivity in adult PTB (overall pooled sensitivity in culture-positive cases)**	≥65% (among both smear-positive & -negative cases)	≥68% (among smear-negative cases only)
**Sensitivity in adult PTB (among smear-positive culture-positive cases only)**	>98%	≥98%
**Specificity**	≥98%	Not specified

2. Community-based triage or referral test to identify people suspected of having TB (World Health Organization, 2014)

	**Minimum screening**	**Optimal screening**
**Sensitivity in adult PTB (compared with confirmatory testing)**	>90%	>95%
**Specificity in adult PTB (compared with confirmatory testing)**	>70%	>80%

3. Test predicting progression from tuberculosis infection to active disease (WHO, 2017) (World Health Organization, 2017)

	**Minimum predictive**	**Optimal predictive**
**Predictive sensitivity**	≥75%	≥90%
**Predictive specificity**	≥75%	≥90%

6.2 CONCOMITANT MEDICATION CODING

MEDICATION NAME	ART	IPT	VIT B6	TB RX	OTHER
(INHALER) ASTHAVENT					Y
2.5 SELSUN					Y
3TC+AZT	Y				
ABACAVIR	Y				
ABACAVIR / LAMIVUDINE	Y				
ABACAVIR LAMIVUDINE	Y				
ABC/3TC	Y				
ACC 200					Y
ACRIPTAZ	Y				
ADCO EFAVIRENZ	Y				
ADCO-EMTERVIR	Y				
ADCO-LAMIVUDINE/ ZIDOVUDINE	Y				
ADCODOL					Y
ADRENALINE					Y
AEVS FIXED DOSE COMBINATION	Y				
ALCOPHYLLEX					Y
ALLERGEX					Y
ALLUVIA	Y				
ALTROIZA	Y				
ALTROIZA (TDF/FTC\EFC)	Y				
ALUVIA	Y				
ALUVIN	Y				
AMITRIPTILLIEN					Y
AMITRIPTILLINE					Y
AMITRIPTYLINE					Y
AMLODIPINE					Y
AMOXICILLIN					Y
AMOXICYCLIN					Y
AMOXICYLLIN					Y
AMOXIL					Y
AMOXYCILIN					Y
AMOXYCILLIN					Y
AMOXYCILLIN CLAVULINIC ACID					Y
AMOXYCLLIN					Y
AMOXYLLIN					Y
AMPCILLIN					Y
AMTAS					Y
ANOXICILLIN					Y
ANTI HYPER TENSIVE ENALAPRIL					Y
ANTIBIOTIC (UNKOWN)					Y
ANTRIPLA	Y				
ANUSOL					Y
AQEOUS CREAM					Y
AQUEOUS CREAM					Y
ART (FDC)	Y				
ART FDC	Y				
ARTROIZA	Y				
ARV	Y				
ARV (FDC)	Y				
ARV'S (FDC)	Y				
ARV'S FDC	Y				
ARV(FDC)	Y				
ARVS (FDC)	Y				
ASHTAVENT					Y
ASTHAVENT					Y
ATANEF	Y				
ATAZA	Y				
ATAZANAVIR	Y				
ATAZOR	Y				
ATENEF	Y				
ATENEF (FDC)	Y				
ATENET	Y				
ATIZAVIRE	Y				
ATOIZA	Y				
ATRAZA	Y				
ATRIPLA	Y				
ATRIZA	Y				
ATROIZA	Y				
ATROIZA (FDC)	Y				
ATROIZA FDC	Y				
ATROIZIA	Y				
ATROZIA	Y				
AUGMENTIN					Y
AUGMNTIN					Y

AUGUMENTIN					Y
AUSTELL PARACETAMOL					Y
AZITHRO					Y
AZITHROMYCIN					Y
AZITHROMYSN					Y
AZT/3TC	Y				
AZT\3TC	Y				
BABALAS REMEDY					Y
BACTRIM					Y
BECLATE					Y
BETADINE					Y
BETAGESIC					Y
BRUFEN					Y
BUDEFLAM					Y
BUDESONIDE					Y
BURFEN					Y
CA GLUCOMAL					Y
CALCIUM GLUCENT					Y
CALGITROL					Y
CALIUM CHLORIED IN 200ML NACL					Y
CALIUM CHLORIED IN 200ML NACL					Y
CAMBIVIR	Y				
CARBAMAZEPINE					Y
CARBAMAZEPINE (TEGRETOL)					Y
CARBIMAZOLE					Y
CEFTRIAXONE					Y
CHLOPHENIRAMINE MALEATE					Y
CHLOROPHERAMINE					Y
CHLORPHENARIMINE MALEATE					Y
CHLORPHENIRAMINE					Y
CHLORPHENIRAMINE MALEATE					Y
CHLORPHERAMINE					Y
CHRORPHENIRAMINE MALEATE					Y
CIFRAN					Y
CIPROBAY					Y
CIPROFLOX					Y
CITALOPRAM					Y
CLAXACILLIN					Y
CLEXANE					Y
CLOFAZIMINE				Y	
CLOMTRIMAZOLE					Y
CLOTRIMAZOLE					Y

CO AMOXY CLAN					Y
CO TRIMOXAZOLE					Y
CO-TRIMOXAZOLE					Y
CORTIMOXAZOLE					Y
COTRANAZOLE					Y
COTRIMOXAZOLE					Y
COTROMOXAZOLE					Y
COZOLE					Y
CR500 EPILIM					Y
DEPO PROVERA					Y
DEPO-PROVERA					Y
DEPO. PROVERA					Y
DEPOT PROVERA					Y
DEPRO PROVERA					Y
DIAZEPAM					Y
DIPHENHYDRAMINE					Y
DIPHENHYDRAMINE HYDROCHLORIDE					Y
DISPRIN					Y
DOXYCYCLINE					Y
DUMIVA	Y				
DUROBAC					Y
DYNASPOR					Y
EFARAVINS	Y				
EFAVARENCE	Y				
EFAVARENZ	Y				
EFAVARENZE	Y				
EFAVERENZ	Y				
EFAVIRENCE	Y				
EFAVIRENE	Y				
EFAVIRENZ	Y				
EFAVIRENZ/ EMTRICITABINE/ TENOFOVIR	Y				
EFAVIRENZE	Y				
EFAVIREZ	Y				
EFEVARENZ	Y				
EFIVARENS	Y				
EFIVARENZ	Y				
EFV	Y				
ELTROXIN					Y
EMCTRITABINE	Y				
EMICITRICITABINE	Y				
EMITIRICATIBANE	Y				

**EMITRACITABINE**	Y				
**EMITRICIBALE**	Y				
**EMITRICIBANE**	Y				
**EMITRICIBATE**	Y				
**EMITRICIBIN/TDF/EFV**	Y				
**EMITRICITABANE**	Y				
**EMITRICITABATE**	Y				
**EMITRICITABIN**	Y				
**EMITRICITABINE**	Y				
**EMITRICITIBINE**	Y				
**EMTRICIBATINE**	Y				
**EMTRICITABIEN**	Y				
**EMTRICITABINE**	Y				
**EMTRICITATABINE**	Y				
**ENALAPRIL**					Y
**ENUTRICUTABINE**	Y				
**ENVAS**					Y
**ENVASLO**					Y
**EPILIM**					Y
**EPILIUM CR**					Y
**ERTAPENEM**					Y
**ERYTHROMYCIN**					Y
**ESONIAZID**		Y			
**ETC**	Y				
**ETENOGEREL**					Y
**ETHAMBUTOL**				Y	
**ETHIONAMIDE**				Y	
**ETONOGESTREL**					Y
**FDC**	Y				
**FDC (TRNOFIVIR, EFAVERENZ, EMTRICITABINE)**	Y				
**FDC (ARVS)**	Y				
**FDC (TDF/EMC/EFV)**	Y				
**FDC ART**	Y				
**FDC ARV**	Y				
**FDC ARV (TRIBUSS)**	Y				
**FDC ARV TRIBUSS**	Y				
**FDC ARVS**	Y				
**FDC ATROIZA**	Y				
**FDC TESOFIEL**	Y				
**FDC TRIBUSS**	Y				
**FE 504**					Y
**FERROUS SULPHATE**					Y

**FIXED DOSE (TDF, FTC,EFV)**	Y				
**FIXED DOSE ANTI RETROVIRAL THERAPY**	Y				
**FIXED DOSE ARV'S**	Y				
**FIXED DOSE COMBINATION ARVS**	Y				
**FIXED DOSE COMBINATION (TDF, EMB, EFV)**	Y				
**FIXED DOSE COMBINATION ART**	Y				
**FIXED DOSE COMBINATION ARV**	Y				
**FLAGYL**					Y
**FLUCLOXICALLIN**					Y
**FLUCONAZOLE**					Y
**FLUOXETINE**					Y
**FLUPAYNE**					Y
**FLUTEX**					Y
**FOLIC ACID**					Y
**FTC**	Y				
**GENTAMICIN**					Y
**GRAND-PA**					Y
**HCTZ**					Y
**HYDROCHLOROTHIAZIDE**					Y
**HYDROCHLOROTHYAZIDE**					Y
**HYDROCHROTHIAZIDE**					Y
**HYDROCURNICHE**					Y
**HYPACE**					Y
**IBUNATE**					Y
**IBUPROFEN**					Y
**ILVITRIM**					Y
**IMPLANON**					Y
**IMPLANT**					Y
**INDO AMOXYCILLIN**					Y
**INH**		Y			
**INSONIAZIDE**		Y			
**IONIAZID**		Y			
**IRON SUPPLEMENTATION**					Y
**ISINIAZID**		Y			
**ISIONAZID**		Y			
**ISIONIAZID**		Y			
**ISONAIZED**		Y			
**ISONAIZID**		Y			
**ISONAZIAD**		Y			
**ISONIAID**		Y			
**ISONIAZED**		Y			

ISONIAZIAD		Y			
ISONIAZID		Y			
ISONIAZIDE		Y			
ISONIZAZID		Y			
IWH		Y			
IZONAZAID		Y			
IZONIAZIDE		Y			
KANAMYCIN				Y	
KAVIMUN	Y				
KIVEXA	Y				
LACSON					Y
LACTULOSE					Y
LAMIVADINE, ZIDOVIDINE	Y				
LAMIVIDUNE	Y				
LAMIVUDENE / IDOVUDINE	Y				
LAMIVUDINE	Y				
LAMIVUDINE / ZIDOVUDINE	Y				
LAMIVUDINE AND ZIDOVUDINE	Y				
LAMSOPRAZOLE					Y
LAMUVIDINE	Y				
LAMZID EFAVIRENZ	Y				
LANSELOC					Y
LANSOLOC					Y
LAPINAVIR	Y				
LASIX					Y
LEROFLXICIN				Y	
LIPONAVIR	Y				
LIQUID PARAFIN					Y
LOPERAMIDE					Y
LOPINAVIR	Y				
LOPINAVIR / RITONAVIR	Y				
LOPINAVIR/ RITONAVIR	Y				
LOPINAVIR/ RITORAVIR	Y				
LOPINOVIR	Y				
LORAZEPAM					Y
LOSEC					Y
LUMIVUDINE	Y				
LUSIX					Y
MAXALON					Y
MED LEMON					Y
MEDROXY PROGESTERONE ACETATE					Y
MEDROXYPROGE STERONE ACETATE					Y

MEDROXYPROGESTERONE					Y
MEDROXYPROGESTERONE ACETATE					Y
MEDROXYPROGESTRONE ACETATE					Y
METOCLOPRAMIDE					Y
METOCLOPROMIDE					Y
METROCLOPRAMIDE					Y
METRONIDAZOLE					Y
MGS04 IN 200ML NACL					Y
MIST POT CHLOR					Y
MORPHINE					Y
MOXIFLAXACIN					Y
MOXIFLOXACIN				Y	
MOXYMAX					Y
MULTIVIT					Y
MULTIVITAMIN					Y
MULTIVITS (VIT B6)					Y
MVT					Y
NAPAMOL					Y
NEVARAPINE	Y				
NEVIRAPIN	Y				
NEVIRAPINE	Y				
NORDETTE					Y
NORETHISTERONE ENANTHATE					Y
NORETHISTHERONE ENANTHATE					Y
NORGESTREL/ ETHINYL OESTRADIOL					Y
NORMAL SALINE					Y
NOZER OMEPRAZOLE					Y
NU ISTERATE					Y
NU-ISTERATE					Y
NUCOTRIM					Y
NUR ISTERATE					Y
NUR ISTRATE					Y
NUR-ISTERATE					Y
NUR-ISTRATE					Y
NURISTERATE					Y
NURISTRATE					Y
NYSTATIN					Y
ODIMUNE	Y				
OMEPRAZOLE					Y
OMEPROZOLE					Y
ORALCON					Y
OVARAL					Y

OVRAL					Y
OXYMETALOLINE					Y
OXYMETAZOLINE HYDRACHLORIDE					Y
PAINBLOCK					Y
PAINBLOK					Y
PANADO					Y
PARACETAMOL					Y
PARACETEMOL					Y
PERTOGEN					Y
PETEGEN					Y
PETOGEN					Y
PHARMAPRESS					Y
PHENERGAN					Y
PHOLCODINE					Y
PIRIDOXINE			Y		
POTASSIUM CHLORIED					Y
PREDISONE					Y
PREDNISONE					Y
PROPAN					Y
PURBAC TABS					Y
PYIRIDOXINE			Y		
PYNDOXINE			Y		
PYRAZANIMIDE				Y	
PYRAZINAMIDE				Y	
PYREDOXIN			Y		
PYRID0XINE			Y		
PYRIDIXINE			Y		
PYRIDOXIDE			Y		
PYRIDOXIN			Y		
PYRIDOXINE			Y		
PYRIDOXINE (VITAMIN B)			Y		
PYRIDOXIXE			Y		
PYRIODOXINE			Y		
PYRIXODINE			Y		
PYRODOXCIN			Y		
PYRODOXINE			Y		
PYRYDOXINE			Y		
RANITIDINE					Y
REFAFOUR E-275				Y	
REPIVATE					Y
RESPIRADONE					Y
RETONOVIR	Y				

RIDA2					Y
RIDAQ					Y
RIFAFOUR				Y	
RIFAFOUR E-275				Y	
RIFINAH				Y	
RIMACTANE		Y			
RINGERS LACTATE					Y
RISPERIDONE					Y
RITENOVIR	Y				
RITONAVIR	Y				
RITONOVIR	Y				
RIZENE	Y				
ROCEPHIN					Y
SALBUTAMOL (ASTHAVENT)					Y
SALTERPYN					Y
SELSYN 2-5/.					Y
SIMVASTATIN					Y
SIRTURO					Y
SONKE EFAVIRENZ	Y				
STAVUDINE	Y				
STILPAYNE					Y
SULFAMETHOXAZOLE					Y
SULPHA TRIMETH					Y
TDF	Y				
TDF \ EFV\ ETC	Y				
TDF, FTC, EFV	Y				
TDF,EMT,EFV	Y				
TDF/ EFV/ FTC	Y				
TDF/ EMT/EFV	Y				
TDF/ FCT/EFV	Y				
TDF/DTC/EFV	Y				
TDF/EFV/ETC	Y				
TDF/EFV/FTC	Y				
TDF/EMT/EFV	Y				
TDF/EMT/EFV (ART)	Y				
TDF/ETB/EFV	Y				
TDF/ETD/EFV	Y				
TDF/FTC	Y				
TDF/FTC/EFC	Y				
TDF/FTC/EFV	Y				
TDF\EMI\EFV	Y				
TDF\EMT\EFV	Y				

**TDF\ETB\EFV**	Y				
**TDF\ETC\EFV**	Y				
**TDF\EVNT\EFV**	Y				
**TDF\FTC\EFV**	Y				
**TENEMINE**	Y				
**TENO/EMTR/EFA**	Y				
**TENOFEVIR**	Y				
**TENOFIVER**	Y				
**TENOFIVIR**	Y				
**TENOFORVIR**	Y				
**TENOFOUIR**	Y				
**TENOFOVIR**	Y				
**TENOFOVIR/ EMTRICITABINE/EFAVIRENZ**	Y				
**TENOVIR**	Y				
**TENOVOFIR**	Y				
**TERIZIDONE**				Y	
**THEOPHLLINE**					Y
**THIAMINE**					Y
**THYROXINE**					Y
**TRAMADOL**					Y
**TRAMADUL**					Y
**TRAPHASIL**					Y
**TRIBUS**	Y				
**TRIBUS (TDF/EFT/EFV)**	Y				
**TRIBUS (TDF, FTC. EFV)**	Y				
**TRIBUS (TDF/EMT/EFV)**	Y				
**TRIBUS (TDF/FTC/EFN)**	Y				
**TRIBUS (TDF/FTC/EFV)**	Y				
**TRIBUS (TDF\FTC\EFN)**	Y				
**TRIBUS (TDF\FTC\EFV)**	Y				
**TRIBUS. (TDF/FTC/EFV**	Y				
**TRIBUS(TDF\FTC\EFV)**	Y				
**TRIBUS(TDF|EMT|EFV)**	Y				
**TRIBUS(TDF|FTC|EFV)**	Y				
**TRIBUSS**	Y				
**TRIBUSS (TDF,3TC,ETC)**	Y				
**TRIBUSS (TDF/EDC/EFV)**	Y				

**TRIBUSS (TDF/EMT/EFV)**	Y				
**TRIBUSS (TDF/FTC/EFV)**	Y				
**TRIBUSS (TDF\3TC/EFV)**	Y				
**TRIPHASIL**					Y
**TRISUS (TDF\FTC\EFV)**	Y				
**TRIVENZ**	Y				
**TRIXAZOLE**					Y
**TRIZANOLE**					Y
**TRUVADA**	Y				
**UNKNOWN ART**	Y				
**UNKNOWN ARV**	Y				
**UNKNOWN PAIN KILLERS**					Y
**UNKNOWN SINGLE DOSE ART REGIMENT**	Y				
**UNKNOWN SINGLE DOSE. ARV TABLETS**	Y				
**VASTOR**					Y
**VENTEZE**					Y
**VIIT C**					Y
**VIT B**					Y
**VIT B6**					Y
**VIT BCO**					Y
**VIT C**					Y
**VITAMIN B COMPLEX**					Y
**VITAMIN B-CO**					Y
**VITAMIN B6**					Y
**VITAMIN BCO**					Y
**VITAMIN C**					Y
**VITB**					Y
**VITB 6**			Y		
**VOLTAREN**					Y
**WINTROP ISONIAZIDE**		Y			
**ZETENOL**					Y
**ZIDOMAT**	Y				
**ZIDOVUDINE**	Y				
**ZIDOVUDINE/LAMIVUDINE**	Y				
**ZIOLOVUDINE**	Y				
**ZIVOLAM**	Y				
**ZOVILAM**	Y				

7 REFERENCES

Aalen O. 1978. Nonparametric inference for a family of counting processes. *The Annals of Statistics.*701-726.

Auguste P, Tsertsvadze A, Pink J, Court R, McCarthy N, Sutcliffe P, and Clarke A. 2017. Comparing interferon-gamma release assays with tuberculin skin test for identifying latent tuberculosis infection that progresses to active tuberculosis: systematic review and meta-analysis. *BMC Infect Dis.* 17 (1):200. doi: 10.1186/s12879-017-2301-4.

Carpenter J, and Bithell J. 2000. Bootstrap confidence intervals: when, which, what? A practical guide for medical statisticians. *Statistics in Medicine.* 19 (9):1141-1164. doi: 10.1002/(sici)1097-0258(20000515)19:9<1141::Aid-sim479>3.0.Co;2-f.

Darboe F, Mbandi SK, Thompson EG, Fisher M, Rodo M, van Rooyen M, Filander E, Bilek N, Mabwe S, Hatherill M*, et al.* 2018. Diagnostic performance of an optimized transcriptomic signature of risk of tuberculosis in cryopreserved peripheral blood mononuclear cells. *Tuberculosis.* 108:124-126. doi: 10.1016/j.tube.2017.11.001.

Darboe F, Mbandi SK, Naidoo K, Yende-Zuma N, Lewis L, Thompson EG, Duffy FJ, Fisher M, Filander E, van Rooyen M*, et al.* 2019. Detection of Tuberculosis Recurrence, Diagnosis and Treatment Response by a Blood Transcriptomic Risk Signature in HIV-Infected Persons on Antiretroviral Therapy. *Front Microbiol.* 10:1441. doi: 10.3389/fmicb.2019.01441.

DeLong ER, DeLong DM, and Clarke-Pearson DL. 1988. Comparing the Areas under Two or More Correlated Receiver Operating Characteristic Curves: A Nonparametric Approach. *Biometrics.* 44 (3). doi: 10.2307/2531595.

Heagerty PJ, Lumley T, and Pepe MS. 2000. Time-dependent ROC curves for censored survival data and a diagnostic marker. *Biometrics.* 56 (2):337-44.

Koopman P. 1984. Confidence intervals for the ratio of two binomial proportions. *Biometrics.*513-517.

Nam JM. 1995. Confidence limits for the ratio of two binomial proportions based on likelihood scores: non-iterative method. *Biometrical Journal.* 37 (3):375-379.

Penn-Nicholson A, Scriba TJ, Hatherill M, White RG, and Sumner T. 2016. A novel blood test for tuberculosis prevention and treatment. *S Afr Med J.* 107 (1):4-5. doi: 10.7196/SAMJ.2016.v107.i1.12230.

Pepe M, Longton G, and Janes H. 2009. Estimation and Comparison of Receiver Operating Characteristic Curves. *Stata J.* 9 (1):1.

Robin X, Turck N, Hainard A, Tiberti N, Lisacek F, Sanchez JC, and Muller M. 2011. pROC: an open-source package for R and S+ to analyze and compare ROC curves. *BMC Bioinformatics.* 12:77. doi: 10.1186/1471-2105-12-77.

World Health Organization. 2014. High-priority target product profiles for new tuberculosis diagnostics: report of a consensus meeting. Geneva: WHO. www.who.int/tb/publications/tpp_report/en/ (Accessed 12 August 2017).

World Health Organization. 2015. The End TB Strategy: Global strategy and targets for tuberculosis prevention, care and control after 2015. Geneva: WHO. https://www.who.int/tb/strategy/End_TB_Strategy.pdf?ua=1 (Accessed September 6, 2019).

World Health Organization. 2017. Consensus Meeting Report: Development of a Target Product Profile (TPP) and a framework for evaluation for a test for predicting progression from tuberculosis infection to active disease. Geneva: WHO. http://apps.who.int/iris/handle/10665/259176 (Accessed 1 October 2017).

Zak DE, Penn-Nicholson A, Scriba TJ, Thompson E, Suliman S, Amon LM, Mahomed H, Erasmus M, Whatney W, Hussey GD*, et al.* 2016. A blood RNA signature for tuberculosis disease risk: a prospective cohort study. *Lancet.* 387 (10035):2312-2322. doi: 10.1016/S0140-6736(15)01316-1.

STARD 2015: an updated list of essential items for reporting diagnostic accuracy studies

Section & Topic	No	Item	Reported on page
**TITLE OR ABSTRACT**			
	1	Identification as a study of diagnostic accuracy using at least one measure of accuracy (such as sensitivity, specificity, predictive values, or AUC)	**p1-2**
**ABSTRACT**			
	2	Structured summary of study design, methods, results, and conclusions (for specific guidance, see STARD for Abstracts)	**p2**
**INTRODUCTION**			
	3	Scientific and clinical background, including the intended use and clinical role of the index test	**p5-6**
	4	Study objectives and hypotheses	**p6**
**METHODS**			
Study design	5	Whether data collection was planned before the index test and reference standard were performed (prospective study) or after (retrospective study)	**p6**
Participants	6	Eligibility criteria	**p7**
	7	On what basis potentially eligible participants were identified (such as symptoms, results from previous tests, inclusion in registry)	**p7**
	8	Where and when potentially eligible participants were identified (setting, location and dates)	**p7**
	9	Whether participants formed a consecutive, random or convenience series	**p7**
Test methods	10a	Index test, in sufficient detail to allow replication	**Supp. p5**
	10b	Reference standard, in sufficient detail to allow replication	**p8**
	11	Rationale for choosing the reference standard (if alternatives exist)	**N/A**
	12a	Definition of and rationale for test positivity cut-offs or result categories of the index test, distinguishing pre-specified from exploratory	**p8**
	12b	Definition of and rationale for test positivity cut-offs or result categories of the reference standard, distinguishing pre-specified from exploratory	**N/A**
	13a	Whether clinical information and reference standard results were available to the performers/readers of the index test	**p7**
	13b	Whether clinical information and index test results were available to the assessors of the reference standard	**p7**
Analysis	14	Methods for estimating or comparing measures of diagnostic accuracy	**Supp. p5-6**
	15	How indeterminate index test or reference standard results were handled	**Supp. p5-6**
	16	How missing data on the index test and reference standard were handled	**Supp. p5-6**
	17	Any analyses of variability in diagnostic accuracy, distinguishing pre-specified from exploratory	**N/A**
	18	Intended sample size and how it was determined	**Study protocol**
**RESULTS**			
Participants	19	Flow of participants, using a diagram	**Figure 1**
	20	Baseline demographic and clinical characteristics of participants	**Table 1**
	21a	Distribution of severity of disease in those with the target condition	**N/A**
	21b	Distribution of alternative diagnoses in those without the target condition	**p14-15**
	22	Time interval and any clinical interventions between index test and reference standard	**p7-8**
Test results	23	Cross tabulation of the index test results (or their distribution) by the results of the reference standard	**Table 1 Figure 1**
	24	Estimates of diagnostic accuracy and their precision (such as 95% confidence intervals)	**Tables 2-3**
	25	Any adverse events from performing the index test or the reference standard	**N/A**
**DISCUSSION**			
	26	Study limitations, including sources of potential bias, statistical uncertainty, and generalisability	**p15**
	27	Implications for practice, including the intended use and clinical role of the index test	**p12-15**
**OTHER INFORMATION**			
	28	Registration number and name of registry	**N/A**
	29	Where the full study protocol can be accessed	**p7**
	30	Sources of funding and other support; role of funders	**p9**

Source: Bossuyt PM, Reitsma JB, Bruns DE, Gatsonis CA, Glasziou PP, Irwig L, et al. STARD 2015: an updated list of essential items for reporting diagnostic accuracy studies. BMJ (Clinical Research Ed). 2015;351:h5527. doi: 10.1136/bmj.h5527.
